# Evaluation of 4‐(4‐Fluorobenzyl)piperazin‐1‐yl]‐Based Compounds as Competitive Tyrosinase Inhibitors Endowed with Antimelanogenic Effects

**DOI:** 10.1002/cmdc.202100396

**Published:** 2021-07-26

**Authors:** Salvatore Mirabile, Serena Vittorio, Maria Paola Germanò, Ilenia Adornato, Laura Ielo, Antonio Rapisarda, Rosaria Gitto, Francesca Pintus, Antonella Fais, Laura De Luca

**Affiliations:** ^1^ Department of Chemical Biological, Pharmaceutical and Environmental Sciences University of Messina Viale Palatucci 13 98168 Messina Italy; ^2^ Department of Life and Environment Sciences University of Cagliari 09042 Monserrato Cagliari Italy; ^3^ Department of Chemistry University of Turin Via P. Giuria 7 10125 Turin Italy

**Keywords:** Synthesis, Tyrosinase inhibitors, Kinetic mechanism, Docking studies, Homology model

## Abstract

There is a considerable attention for the development of inhibitors of tyrosinase (TYR) as therapeutic strategy for the treatment of hyperpigmentation disorders in humans. Continuing in our efforts to identify TYR inhibitors, we describe the design, synthesis and pharmacophore exploration of new small molecules structurally characterized by the presence of the 4‐fluorobenzylpiperazine moiety as key pharmacophoric feature for the inhibition of TYR from *Agaricus bisporus* (AbTYR). Our investigations resulted in the discovery of the competitive inhibitor [4‐(4‐fluorobenzyl)piperazin‐1‐yl]‐(3‐chloro‐2‐nitro‐phenyl)methanone **26** (IC_50_=0.18 μM) that proved to be ∼100‐fold more active than reference compound kojic acid (IC_50_=17.76 μM). Notably, compound **26** exerted antimelanogenic effect on B16F10 cells in absence of cytotoxicity. Docking analysis suggested its binding mode into AbTYR and into modelled human TYR.

## Graphical abstract

**The design, synthesis**, and pharmacophore exploration of new molecules structurally characterized by the 4‐fluorobenzylpiperazine fragment are reported. Their inhibitory effects on *Agaricus bisporus* tyrosinase (AbTYR) were evaluated. Notably, the competitive derivative **26** revealed no cytotoxicity and exerted antimelanogenic effects on B16F10 cells. Docking analysis suggested its binding mode into AbTYR and into modelled human TYR.

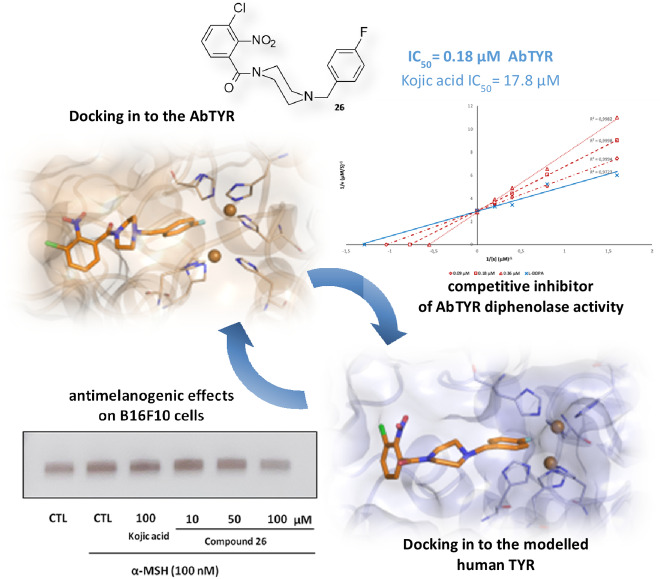

## 1 Introduction

Tyrosinase (TYR, EC 1.14.18.1) is a type 3 copper‐containing enzyme distributed in bacteria, fungi, plants and animals. TYR is the rate‐limiting enzyme in melanogenesis catalyzing a multistep conversion of mono‐ or di‐phenolic compounds (*e. g*. L‐Tyrosine, L‐DOPA) to give eumelanin and pheomelanin.[^1^, ^2^] Therefore, TYR plays multiple roles in pigmentation, UV radiation and free radicals protection, undesirable browning of fruits and vegetables. Specifically, in humans the abnormal lack of melanin could be responsible of albinism; whereas an excessive melanin accumulation can result in hyperpigmentation related disorders.^[3]^ Indeed, tyrosinase inhibitors (TYRIs) are considered potential skin‐whitening agents, antimelanogenic substances for melanoma treatment. Moreover, several TYRIs might be innovative adjuvants in Parkinson's disease therapies related to neurodegenerative effects of neuromelanin, as well as drugs for bacterial infections.[^4^, ^5^]

TYR structure includes a central domain, a N‐terminal domain and a transmembrane region.^[3]^ In the central domain, there are two copper ions (CuA and CuB) bound to two set of three conserved histidine residues involved in the TYR‐mediated oxidation process.^[6]^ To date few crystal structures of TYR from different organisms are available. Among them, the bacterial enzyme from *Bacillus megaterium* ^[7]^ and mushroom enzyme from *Agaricus bisporus* (AbTYR)^[8]^ are well‐characterized and represent a model for the rational TYRIs design.

Furthermore, the commercial availability at low cost of purified form of the AbTYR offers the opportunity to largely employ it in enzymatic inhibition screening for the preliminary evaluation of TYRIs by using Kojic Acid (KA) as reference compound. Thus, the most TYRIs reported in literature were generally identified on the exclusive basis of inhibitory effects toward AbTYR.

Apart from natural products such as KA[^9^, ^10^] and several flavonoid‐based derivatives, there is a large pool of molecules from synthetic source possessing inhibitory effects against TYRs from various organisms.[^5^, ^11^, ^12^, ^13^]

**Figure 1:**
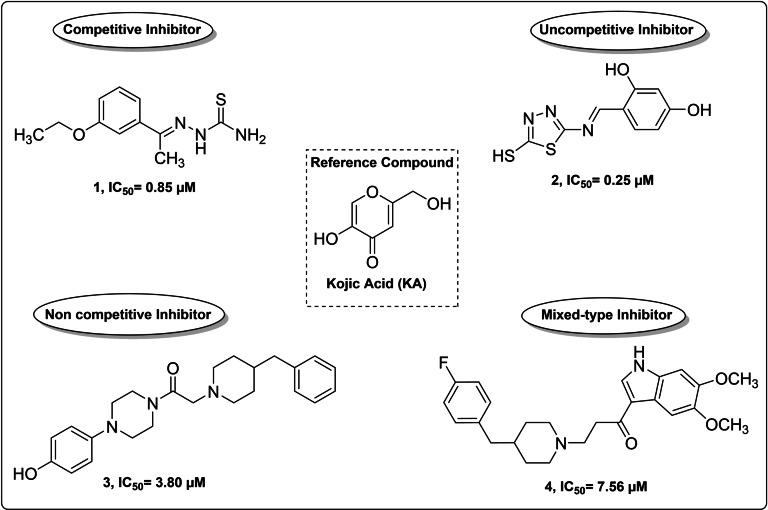
Chemical structures of representative TYRIs displaying efficacy against AbTYR.

On the basis of mechanism of inhibition, TYRIs are generally classified into four categories as competitive, uncompetitive, noncompetitive and mixed‐type inhibitors (Figure 1) as illustrated for prototypical AbTYR inhibitors listed below.

The competitive inhibitor KA as well as 2‐(1‐(3‐ethoxyphenyl)ethylidene)hydrazine‐1carbothioamide (**1**)^[15]^ occupy the active site thus preventing the substrate binding, while the uncompetitive inhibitor 4‐(((5‐mercapto‐1,3,4‐thiadiazol‐2‐yl)imino)methyl)benzene‐1,3‐diol (**2**)^[16]^ is capable to exclusively bind the enzyme−substrate complex. The noncompetitive inhibitor 2‐(4‐benzyl‐1‐piperidyl)‐1‐[4‐(4‐hydroxyphenyl)piperazin‐1‐yl]ethanone (**3**)^[17]^ might bind the enzyme in a different region compared to the true substrates that recognize the catalytic site.

Finally, the binding of the mixed‐type inhibitor 1‐(5,6‐dimethoxy‐1H‐indol‐3‐yl)‐3‐(4‐(4‐fluorobenzyl)piperidin‐1‐yl)propan‐1‐one (**4**) occurs with both free enzyme and enzyme–substrate complex.^[18]^

**Figure 2:**
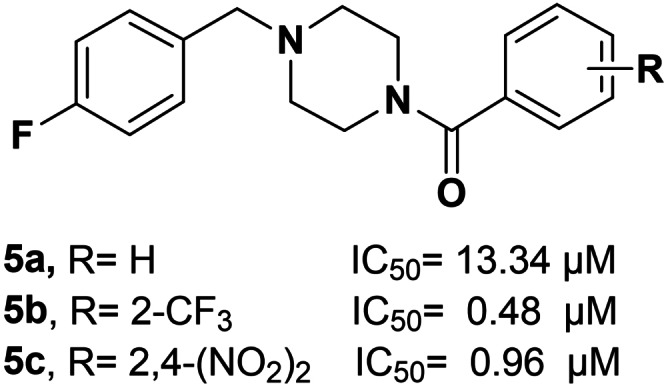
Chemical structures of representative TYRIs bearing 4‐(fluorobenzyl)piperazine moiety.

Considerable amount of our efforts has been addressed to improve the TYR affinity of 4‐(fluorobenzyl)piperazine compounds inspired by prototype **4**. In detail, we have recently reported the two excellent inhibitors [4‐(4‐fluorobenzyl)piperazin‐1‐yl](2‐trifluoromethyl)methanone (**5 b**) and [4‐(4‐fluorobenzyl)piperazin‐1‐yl](2,4‐dinitrophenyl)methanone (**5 c**) that were obtained by optimization of parent compound 4‐(4‐fluorobenzyl)piperazin‐1‐yl]phenylmethanone (**5 a**)^[14]^ (Figure 2) through the introduction of suitable modification on the arylic tail.

Interestingly, kinetic studies on the diphenolase activity of AbTYR have confirmed that the key ligand **5 c** acted as a competitive inhibitor.^[14]^ This mechanism of action was consistent with the theoretical and experimental structural studies; specifically, a combination of X‐ray and docking simulations has confirmed that **5 c** is anchored to TYR catalytic site through the 4‐fluorobenzilic portion that is oriented towards the Cu atoms and the substituted arylic portion is oriented toward the top of the cavity.

Here, we report the design of a new series of twenty‐six analogs in which the 4‐fluorobenzylpiperazine fragment was maintained as primary building block bearing second key feature that was selected to engage ancillary hydrophobic/hydrophilic interactions with the top region of the catalytic site as suggested by X‐ray and docking studies.[^14^, ^17^, ^18^, ^19^, ^20^] All new designed and synthesized compounds were preliminary tested for *in vitro* inhibiting AbTYR thus upgrading our knowledge about structural affinity relationship (SAR) information for this class of compounds. For selected potent inhibitors we also deciphered the mode of inhibition in kinetic assays. To analyze the binding interaction within catalytic cavity of AbTYR we performed docking analysis. Then, we extended our docking study toward the most druggable human TYR (hTYR) and performed a 3D homology model as a surrogate of experimental data currently not available for hTYR. Finally, the safety and the efficacy of the compounds were also tested using B16F10 melanoma cell model.

## 2 Results and Discussion

### 2.1 Chemistry

**Scheme 1:**
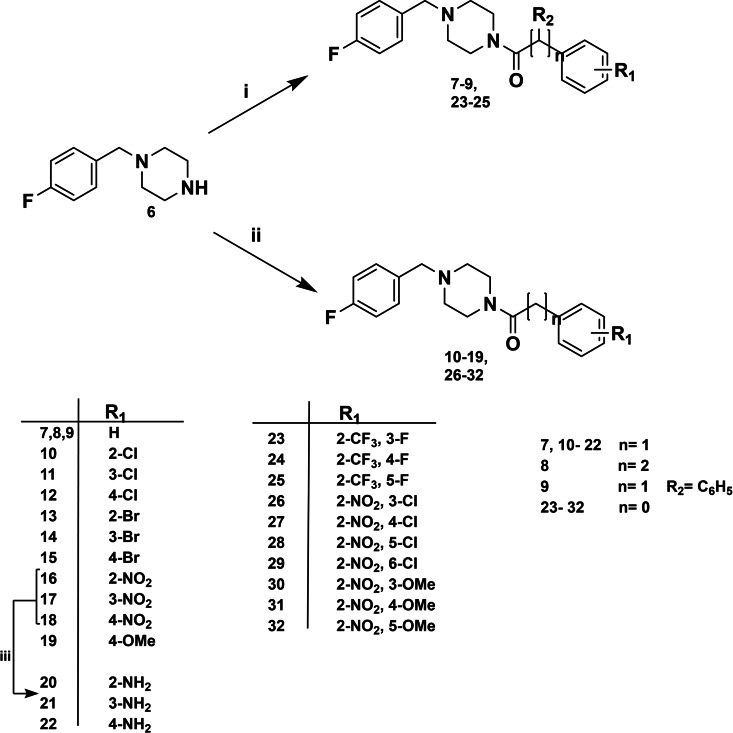
Reagents and conditions: i) acyl chloride derivatives, DIPEA, DCM, 5 h, rt; ii) carboxylic acid derivatives, HBTU, TEA, DMF, MW: 10 min, 50 °C, 200 W; iii) Hydrazine, Pd/C, EtOH, reflux, 2 h.

Scheme 1 depicts the two rounds of structural modifications that were based on the promising achievements of previously reported compounds **5 a**–**c**. The first series of new designed compounds was inspired by **5 a** (R=H, IC_50_=13.34 μM) that had displayed activity comparable to that of KA (IC_50_=17.76 μM) against AbTYR diphenolase activity. Then, the new analogs **7**–**22** were designed to expand our knowledge about SARs for this class of inhibitors. Specifically, we have initially looked for the optimal distance between the centroid of the aromatic ring of the 4‐fluorobenzylpiperazine and the aroyl moiety, by adding carbon atoms as methylenic or ethylenic linker in compound **7**–**8**. Moreover, we explored the steric demand by introducing additional phenyl ring on methylene bridge in compound **9**. Furthermore, we examined the impact of variously decorated aroylic portion bearing a set of substituents as EDGs (Electron Donating Groups) or EWGs (Electron Withdrawing Groups). The second series of compounds **23**–**32** was designed in attempt to establish more favorable contacts into the hydrophobic pocket in the top region of the cavity. Based on the high potency of prototypes **5 b** and **5 c**, we chose the 2‐CF_3_ or 2‐NO_2_ as relevant key features; therefore, we introduced additional fluorine/chlorine atoms or nitro/methoxy groups in different positions of aromatic ring.

As shown in Scheme 1 the twenty‐three compounds **7**–**19** and **23**–**32** were readily synthesized by coupling 4‐(4‐fluorobenzyl)piperazine (**6**) with the suitable and commercial available acyl chloride or carboxylic acid under basic conditions. In addition, for the activation of carboxylic acids, the common coupling agent HBTU was employed. The coupling reaction was carried out at room temperature or under microwave conditions. The three amino‐derivatives **20**–**22** were prepared by nitro‐reduction of parent compounds **16**–**18**. All twenty‐six new synthesized compounds were structurally characterized; the ^1^H‐NMR and ^13^C‐NMR spectra were in full agreement with the proposed structures (for details see Experimental section and Supporting Information).

### 2.2 Assessment of AbTYR inhibitory effects

We evaluated the inhibitory effects of all the synthesized molecules by using AbTYR in the presence of L‐DOPA. The obtained results are reported in Table 1, in which we compared the IC_50_ values of compounds **7**–**32** with **5 a** (R=H), **5 b** (R=2‐CF_3_), **5 c** (R=2,4‐(NO_2_)_2_) and KA as reference compounds.

**Table 1:** AbTYR inhibitory effects **7**–**32**, **5 a**–**c** and kojic acid (KA)

Compd	*n*	R^1^	R^2^	Diphenolase activity IC_50_ [μM]^[a]^
**5 a**	0	H	H	13.34±0.73
**5 b**	0	2‐CF_3_	H	0.48±0.05
**5 c**	0	2,4‐(NO_2_)_2_	H	0.96±0.21
**7**	1	H	H	4.28±0.86
**8**	2	H	H	13.17±2.71
**9**	1	H	Ph	40.43±0.81
**10**	1	2‐Cl	H	8.70±0.86
**11**	1	3‐Cl	H	4.66±0.89
**12**	1	4‐Cl	H	4.71±0.63
**13**	1	2‐Br	H	4.70±0.65
**14**	1	3‐Br	H	15.74±0.69
**15**	1	4‐Br	H	7.66±0.42
**16**	1	2‐NO_2_	H	8.31±1.56
**17**	1	3‐NO_2_	H	8.66±0.78
**18**	1	4‐NO_2_	H	1.71±0.79
**19**	1	4‐OMe	H	19.63±1.58
**20**	1	2‐NH_2_	H	3.74±0.54
**21**	1	3‐NH_2_	H	9.36±0.45
**22**	1	4‐NH_2_	H	16.73±0.57
**23**	0	2‐CF_3_,3−F	H	0.45±0.03
**24**	0	2‐CF_3_,4−F	H	0.87±0.01
**25**	0	2‐CF_3_,5−F	H	0.24±0.03
**26**	0	2‐NO_2_,3−Cl	H	0.18±0.03
**27**	0	2‐NO_2_,4−Cl	H	2.27±0.49
**28**	0	2‐NO_2_,5−Cl	H	2.01±0.23
**29**	0	2‐NO_2_,6−Cl	H	1.17±0.18
**30**	0	2‐NO_2_,3−OMe	H	2.09±0.27
**31**	0	2‐NO_2_,4−OMe	H	1.80±0.13
**32**	0	2‐NO_2_,5−OMe	H	1.11±0.09
**KA**	–	–	–	17.76±0.18

[a] IC_50_: concentration that caused 50 % enzyme activity loss. The mean value and standard deviation were calculated from triplicate experiments.

Analyzing the results in Table 1, we can observe that all the novel 4‐fluorobenzylpiperazine derivatives **7**–**32** were active inhibitors, reaching IC_50_ values at low micromolar concentrations. Only the compound **9** (IC_50_=40.43 μM) deviated from the average, proving to be the least effective inhibitor of the series. Probably the presence of an additional aromatic ring on the methylene bridge caused a molecular restriction and/or steric hindrance that reduced the ability to bind the catalytic pocket.

Regarding the linker size, compound **7** (IC_50_=4.28 μM) was more potent AbTYR inhibitor when compared to its homologue **8** (IC_50_=13.17 μM), suggesting that the inhibitory efficacy decreases as the number of carbon atoms increases. Notably, the introduction of nitro group in *para* position allowed us to obtain the most active inhibitor in the mono‐substituted series (compound **18**, IC_50_=1.71 μM). Among the series of compounds characterized by the presence of EDG substituents, the ortho amino derivative **20** demonstrated an IC_50_ value of 3.74 μM, whereas the *meta* and *para* analogues **21** and **22** as well as the 4‐methoxy derivative **19** showed inhibitory effects comparable to that of KA (IC_50_=17.76 μM).

Concerning the halogen‐substituted derivatives, compounds **10**–**15** displayed less activity than their corresponding previously reported analog compounds without methylene bridge that we have reported in a previous paper.^[14]^ Another attractive SAR consideration concerned all disubstituted derivatives bearing −NO_2_ or −CF_3_ groups in ortho position, they were excellent compounds possessing inhibitory efficacy at low micromolar range. Among them, the most promising and potent AbTYR inhibitors were compounds **23**–**26** that showed a better activity than the previously described 2,4‐disubstituted derivatives^[18]^; actually, compound **26** (IC_50_=0.18 μM) was about one hundred‐fold more active than KA. Therefore, the above‐mentioned aromatic substituents were confirmed as optimal features for the enzymatic binding and successful inhibition.

### 2.3 Kinetic studies

Compounds **23**, **25** and **26** were selected for kinetic studies to obtain more information about the mode of action against TYR; thereby their inhibitory effects on diphenolase activity were measured using increasing concentration of L‐DOPA. (0.6–5 mM). The reaction mixture consisted of four different concentrations of L‐DOPA (0.6–5 mM), the substrate, and AbTYR in acetate buffer (0.05 M, pH 6.8).

**Figure 3:**
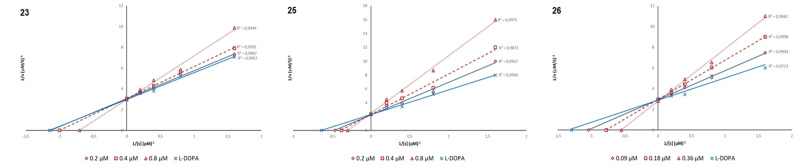
Lineweaver‐Burk plots of compounds **23**, **25** and **26** using increasing concentration of the substrate L‐DOPA. (0.6–5 mM).

Compounds **23** and **25** (0.2, 0.4, and 0.8 μM), **26** (0.09, 0.18, and 0.36 μM) were added to the reaction mixture. The Michaelis‐Menten constant (Km) and maximal velocity (Vmax) of tyrosinase were determined by Lineweaver‐Burk plots. Results are presented using Lineweaver‐Burk double reciprocal plots (see Figure 3) and revealed that the studied compounds were competitive inhibitors of diphenolase activity.

### 2.4 Docking analysis on AbTYR

**Figure 4:**
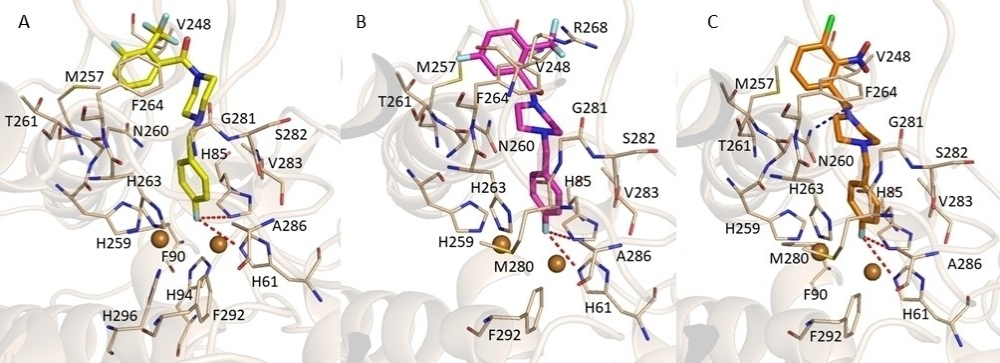
Plausible binding modes for inhibitors **23** (Panel A), **25** (Panel B) and **26** (Panel C) docked into the AbTYR binding site (PDB code 2Y9X). Compound **23** is displayed as yellow sticks, while compounds **25** and **26** are represented as magenta and orange sticks, respectively. The amino acid residues of the binding site are represented by wheat sticks. H‐bonds are displayed as blue dashed lines whereas halogen bonds are highlighted as red dashed lines. The image was created by using PyMOL software (www.pymol.org).

To obtain molecular insights into the binding mode of several selected compounds we performed docking studies by Gold software.^[21]^ In the first step of our computational study we predicted binding interaction to the AbTYR binding pocket (PDB code 2Y9X)^[8]^ (see Figure S43) for compounds **23**, **25** and **26** (Figure 4 A–C). As result we found that they assumed a similar orientation with the 4‐fluorobenzyl portion projected towards the two copper ions (CuA and CuB) engaging π‐stacking interaction with H263 and hydrophobic contacts with V283 and A286. Interestingly, the Discovery Studio Visualizer V20 revealed that the fluorine atom could establish two halogen bonding interactions with the crucial residues H61 and H85 involved in the coordination of copper atom CuA of catalytic site. Interestingly, this finding well matches our previous SAR observations for which a crucial role of the fluorine atom in the inhibition of AbTYR for this class of compounds.^[20]^ Actually, the halogen bonding has been found in many protein‐ligand interactions as favorable feature in drug recognition process. Moreover, the three inhibitors docked into the active site established van der Waals interactions with residues F90, M257, H259, N260, T261, R268, M280, S282 and F292. Finally, hydrophobic contacts with V248 were detected for derivatives **23** and **25**. Compound **23** (Figure 4, Panel A) could also engage aromatic interactions with F264 through the aroyl moiety. Notably, the oxygen of CO of the best inhibitor 26 (Figure 4, Panel C) participates in a H‐bond contact with residue N260, thus anchoring the aroyl moiety in the top region on AbTYR cavity.

### 2.5 Homology modelling and docking studies on hTYR

**Figure 5:**
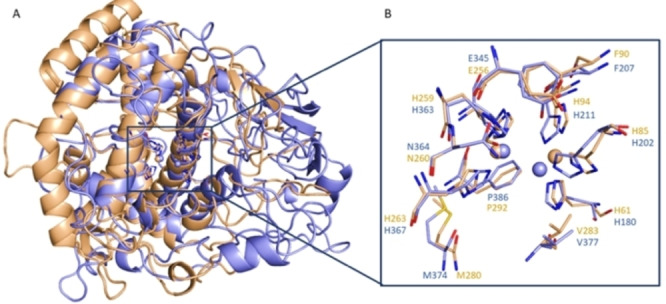
A) Superimposition of hTYR homology model (blue sticks) upon AbTYR (wheat sticks). B) Close view of hTYR and AbTYR active site.

In the second step of our study, we addressed our efforts to collect information about the ability of selected derivatives **23**, **25** and **26** to inhibit the hTYR for potential application in therapy as antimelanogenic agents. To achieve this goal, the data gained through our biological and in silico studies on AbTYR were translated to the human isoform. Considering that no detailed experimental structure of hTYR is currently available, we built a homology model by using the web tool SWISS‐MODEL (https://swissmodel.expasy.org/)^[22]^ and employing as template the crystal structure of human Tyrosinase‐related protein 1 (TYRP‐1) mutant (T391V‐R374S‐Y362F) in complex with KA (PDB code 5 M8Q)^[6]^ for which the sequence alignment is shown in Figure S45 of Supporting Information. In humans the melanin biosynthesis depends on the activity of TYR, TYRP1 and TYRP2 that share 40 % of sequence identity and are characterized by four conserved regions: a N‐terminal signal peptide, an intramelanosomal domain, a single transmembrane α‐helix and a C‐terminal cytoplasmatic portion. The intramelanosomal region possesses a tyrosinase‐like subdomain comprehending a binuclear metal containing active site. Recently, crystallographic structures of TYRP1 in complex with various TYR substrates were solved revealing for the first‐time new insights about a human tyrosinase family member. Differently from TYR, TYRP1 has two zinc ions that make it unlikely to exert a redox activity.^[6]^ Due to the high similarity with hTYR, the crystallographic structure of TYRP1 represents a good template to build a 3D model of hTYR.[^23^, ^24^] As displayed in Figure 5, the two proteins shared a similar catalytic cavity so that the AbTYR binding pocket hosting the 4‐fluorobenzyl moiety is quite conserved in the hTYR (Figure 5B); therefore, we hypothesized that this crucial motif portion might interact with the same region in hTYR. Based on this assumption, the obtained hTYR homology model was used to perform docking studies of the most active inhibitors **23**, **25** and **26** by means of Gold software. The binding site defined for the calculation is displayed in Figure S44 of Supporting Information.

**Figure 6:**
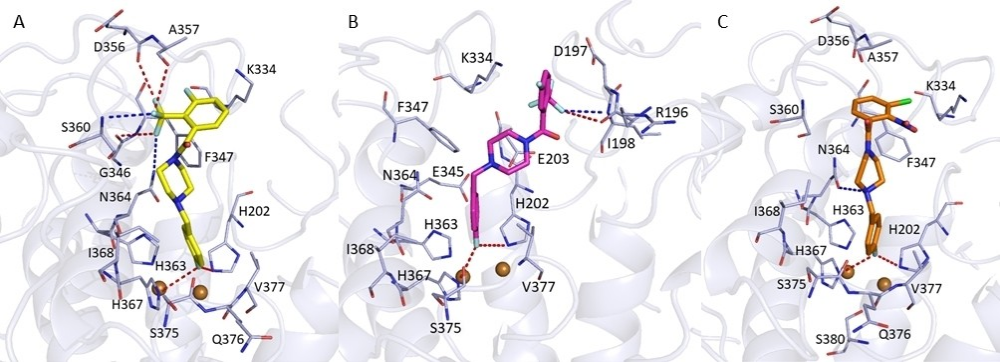
Plausible binding modes for inhibitors **23** (Panel A), **25** (Panel B) and **26** (Panel C) into hTYR binding site. Compound **23** is displayed as yellow sticks, while compounds **25** and **26** are represented as magenta and orange sticks, respectively. The amino acid residues of the binding site are represented by light‐blue sticks. H‐bonds are displayed as blue dashed lines whereas halogen bonds are highlighted as red dashed lines. The image was created by using PyMOL software (www.pymol.org).

Concerning the 4‐flurobenzyl moiety, the docking simulations revealed that all docked compounds might occupy hTYR binding site assuming a similar orientation found for AbTYR (Figure 6). Notably, also in this case the fluorine atom appears capable to establish two halogen bonds with H202 and H367 that correspond to residues H85 and H263 in AbTYR. The 4‐flurobenzyl moiety could engage aromatic interactions with H202 and H367 and additional hydrophobic contacts with residue Val377 (hTYR) that corresponds to Val283 in AbTYR.

Moreover, the docking poses indicated that compound **23** might interact with hTYR active site by several key interactions depicted in Figure 6A: i) two H‐bonds formed by two fluorine atoms of trifluoromethyl group with the backbone NH of S360 and nitrogen atom of residue N364, ii) three halogen bonds formed by two fluorine atoms of trifluoromethyl group and the carbonyl oxygen of residues D356 and A357, iii) aromatic interactions with aromatic ring of residue F347 and benzoyl moiety and iv) hydrophobic contacts with residue A357. Instead, derivative **25** might also establish i) a halogen bond between the trifluoromethyl group and R196, ii) a H‐bond between the trifluoromethyl group and the NH backbone of I198, iii) π‐cation interactions between the benzoyl portion and K334 and iv) a salt bridge between E203 and the amino group of the piperazine ring (Figure 6, Panel B).

Finally, compound **26** could establish additional aromatic interactions with F347 through the benzoyl group and a H‐bond with N364 through the piperazine nitrogen (Figure 6, Panel C). Finally, the three inhibitors might establish van der Waal interactions with K334, F347, A357, H363, I368, S375 and Q376. Overall, the docking outcomes on hTYR highlighted that the 4‐fluorobenzyl moiety is able to occupy the same conserved region present in hTYR, while the rest of the molecules might engage additional interactions with other amino acid residues of the binding site. Based on these findings, we can speculate that this class of TYR inhibitors might bind the catalytic pocket of hTYR inhibiting its enzymatic activity.

### 2.6 Cell viability, effect on cellular tyrosinase activity and melanin production

**Figure 7:**
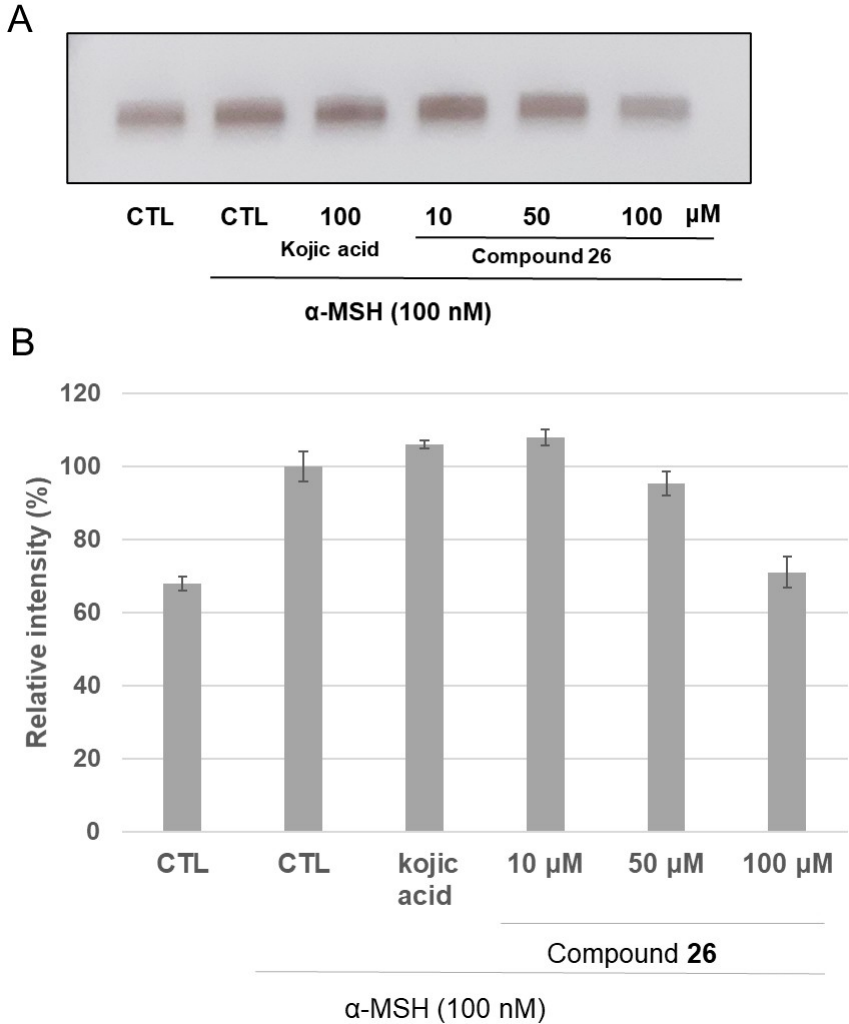
Effect of compound **26** on tyrosinase activity by L‐DOPA staining (A). ImageJ software was used in order to determine the relative intensity of bands (B). The mean value and standard deviation were calculated from triplicate experiments.

MTT assay was used to evaluate the biosafety effectiveness of **23**, **25** and **26**. Cells were treated with different concentration of each compound (0–100 μM), for 48 h, to determine the potential cytotoxic effect on B16F10 cells. At the concentration in which the three compounds inhibited tyrosinase activity no cytotoxic effect in B16F10 melanoma cells was observed (see Supporting Information). It is worth pointing out that at the highest concentration used (100 μM), which is from 200 to 550‐fold higher than the IC_50_ values, the cell viability of the compounds was still greater than 90 %. To achieve a deeper characterization of most promising and potent inhibitor **26**, we further examined its inhibitory effect on the tyrosinase activity in the cellular model. DOPA staining assay was performed using lysates of B16F10 cells untreated or treated with hormone with or without the tested compound **26**. The tyrosinase activity was significantly increased, upon exposure to *α*‐MSH alone, compared to untreated cells. This was evident with the appearance of a darker band compared to that of untreated control (Figure 7). Upon incubation with compound 26, the tyrosinase activity decreased in a dose‐response manner and lighter bands were observed. As result, the inhibitory effects of the compound 26 was also confirmed in the cellular model.

## 3 Conclusion

In summary, we designed and synthesized a series of novel 4‐fluorobenzylpiperazine analogs targeting tyrosinase binding site. All compounds showed *in vitro* inhibition of tyrosinase from *Agaricus Bisporus* (AbTYR) with IC_50_ values in the micromolar range. Among them, derivatives **23**, **25** and **26** demonstrated the best promising activity displaying competitive mechanism and no cytotoxicity. Interestingly, docking studies suggested a direct binding of the best active compounds to the site of modelled human TYR in good accordance with the binding orientation in to AbTYR cavity. Finally, compound **26** displayed antimelanogenic effects on B16F10 cells, thus confirming that it could represent a prospective candidate for further applications as anti‐tyrosinase agent.

## Experimental Section

### Chemistry

Reagents and solvents were bought from commercial suppliers (Sigma‐Aldrich and Alfa Aesar) and were used without further purification. Microwave‐assisted synthetic procedures were carried out in a Focused Microwave^TM^ Synthesis System (Discover CEM, Buckingham, UK). Melting points were determined on a Büchi B‐545 apparatus (BUCHI Labortechnik AG Flawil, Switzerland) and are uncorrected. Combustion analysis (C, H, N) were carried out on a Carlo Erba Model 1106‐Elemental Analyzer; the purity of synthesized compounds was confirmed within ≥95 % values. Flash Chromatography (FC) was performed on a Biotage SP1 EXP (Biotage AB Uppsala, Sweden). ^1^H NMR spectra and ^13^C NMR spectra were measured by a Varian Gemini 500 spectrometer (Varian Inc. Palo Alto, California USA); the chemical shifts were expressed in δ (ppm) and coupling constants (*J*) in hertz (Hz); exchangeable protons were analyzed by addition of deuterium oxide (D_2_O). Mass spectra were recorded with a Bruker maXis 4G instrument (ESI‐TOF, HRMS). TLC plates were used to measure R_*f*_ values by using DCM/MeOH (96 : 4) mixture as eluent.

#### General procedure for the synthesis of 1‐[4‐(4‐fluorobenzyl)piperazin‐1‐yl]‐2‐phenylethan‐1‐one derivatives (7 and 9) and 1‐[4‐(4‐fluorobenzyl)piperazin‐1‐yl]‐3‐phenylpropan‐1‐one (8)

To a solution of 1‐(4‐fluorobenzyl)piperazine (**6**) (200 mg, 1.03 mmol) in dichloromethane (DCM, 2 mL), a mixture of the N,N‐diisopropylethylamine (DIPEA, 268 μL, 1.54 mmol) and suitable acyl chloride (1.13 mmol) was added dropwise. The reaction was stirred at room temperature for 5 hours and quenched with methanol (MeOH, 2 mL). Then, water was added and the mixture was extracted with DCM (3×10 mL). The organic phase was dried with anhydrous Na_2_SO_4_. The solvent was removed under reduced pressure; the final products were purified by flash chromatography (DCM/MeOH 96 : 4). For compounds **7**, **8** and **9** CAS Registry numbers have been already assigned and are available at https://www.cas.org even if are not available the synthetic procedures, chemical properties and structural characterization; therefore, detailed structural information for these three compounds is reported below.

**1‐[4‐(4‐Fluorobenzyl)piperazin‐1‐yl]‐2‐phenylethan‐1‐one (7)**. CAS Number: 423743‐29‐7. Yield 96 %. Oily residue. R_*f*_: 0.51. ^1^H‐NMR (500 MHz, CDCl_3_): (δ) 2.22 (t, *J* **=**4.8 Hz, 2H, CH_2_), 2.39 (t, *J* **=**4.8 Hz, 2H, CH_2_), 3.42 (m, 4H, CH_2_), 3.65 (t, *J* **=**4.6 Hz, 2H, CH_2_), 3.72 (s, 2H, CH_2_), 6.97–7.00 (m, 2H, ArH), 7.23 (m, 5H, ArH), 7.30 (m, 2H, ArH). ^13^C‐NMR (126 MHz, CDCl_3_): (δ) 41.1, 41.9, 46.2, 51.7, 52.9, 62.1, 115.2, 115.3, 126.9, 128.7, 128.8, 130.59, 130.65, 133.4, 135.2, 161.2–163.2 (d, J_C–F_=244 Hz), 169.6. HRMS (ESI), *m/z*: calcd. for C_19_H_22_FN_2_O^+^: 313.1711 [M+H]^+^; found: 313.1720. Anal. Calcd for: (C_19_H_21_FN_2_O) C 73.05, H 6.78, N 8.97. Found: C 73.25, H 6.88, N 8.80.

**1‐[4‐(4‐Fluorobenzyl)piperazin‐1‐yl]‐3‐phenylpropan‐1‐one (8)**. CAS Number: 439848‐20‐1. Yield 100 %. Oily residue. R_*f*_: 0.48. ^1^H‐NMR (500 MHz, CDCl_3_): (δ) 2.28 (t, *J* **=**4.8 Hz, 2H, CH_2_), 2.38 (t, *J* **=**5.0 Hz, 2H, CH_2_), 2.61 (t, *J* **=**7.8 Hz, 2H, CH_2_), 2.96 (t, *J* **=**7.8 Hz, 2H, CH_2_), 3.38 (t, *J* **=**5.0 Hz, 2H, CH_2_), 3.45 (s, 2H, CH_2_), 3.62 (t, *J* **=**4.8 Hz, 2H, CH_2_), 6.98–7.02 (m, 2H, ArH), 7.24 (m, 7H, ArH). ^13^C‐NMR (126 MHz, DMSO‐d_6_): (δ) 30.8, 33.9, 41.1, 44.9, 52.2, 52.6, 60.9, 114.7, 114.9, 125.7, 128.1, 128.3, 128.4, 128.6, 130.5, 130.8, 133.9, 141.4, 160.34–162.27 (d, J_C–F_=243 Hz), 169.8. HRMS (ESI), *m/z*: calcd. for C_20_H_24_FN_2_O^+^: 327.1867 [M+H]^+^; found: 327.1887. Anal. Calcd for (C_20_H_23_FN_2_O): C 73.59, H 7.10, N 8.58. Found: C 73.66, H 7.00, N 8.76.

**1‐[4‐(4‐Fluorobenzyl)piperazin‐1‐yl]‐2,2‐diphenylethan‐1‐one (9)**. CAS Number: 423739‐67‐7. Yield: 72 %. White solid. R_*f*_: 0.65. M.p. 124–126 °C. ^1^H‐NMR (500 MHz, DMSO‐d_6_): (δ) 2.07 (m, 2H), 2.27 (m, 2H), 3.38 (s, 2H, CH2), 3.48 (m, 4H), 5.51 (s, 1H, CH), 7.10–7.14 (m, 2H, ArH), 7.19–7.23 (m, 6H, ArH), 7.27–7.31 (m, 6H, ArH). ^13^C‐NMR (126 MHz, DMSO‐d_6_): (δ) 41.6, 45.5, 52.0, 52.5, 52.6, 60.7, 114.8, 114.9, 126.5, 128.2, 128.9, 130.6, 130.6, 133.9, 140.1, 160.28–162.21 (d, J_C–F_=242 Hz), 169.4. HRMS (ESI), m/z: calcd. for C_25_H_26_FN_2_O^+^: 389.2024 [M+H]^+^; found: 389.2049. Anal. Calcd for (C_25_H_25_FN_2_O): C 77.29, H 6.49, N 7.21. Found: C 77.19, H 6.56, N 7.00.

#### General procedure for the synthesis of 1‐[4‐(4‐Fluorobenzyl)piperazin‐1‐yl]‐2‐phenylethan‐1‐one derivatives (10–19)

A mixture of the amine 6 (200 mg, 1.03 mmol) in triethylamine (TEA, 153 μL, 1.13 mmol) and a solution of the carboxylic acid derivative (1.13 mmol), N,N,N’,N’‐tetramethyl‐O‐(1Hbenzotriazol‐1‐yl)uronium hexafluorophosphate (HBTU, 428 mg, 1.13 mmol) in *N*,*N*‐Dimethylformamide (DMF, 4 mL) were prepared. Then, the amine solution was added dropwise to the second mixture containing HBTU. The reaction was carried out in microwave‐condition for 10 min at 50 °C, and then the mixture was quenched with water (10 mL) and extracted with ethyl acetate (EtOAc, 3x10 mL). The obtained organic phase was washed many times with brine (3x10 mL), dried with Na_2_SO_4_ and, finally, the solvent was removed in vacuo. The crude residues were treated with Et_2_O, Et_2_O/EtOH, hexane (for compound 13) or purified by flash chromatography (DCM/MeOH 96 : 4) thus leading to the final products. For compounds 10, 12–15 and 19 CAS numbers have been already registered. However, due to the lack of details in terms of synthetic procedures, chemical properties and structural characterization, we listed below the above‐mentioned information.

**1‐[4‐(4‐Fluorobenzyl)piperazin‐1‐yl]‐2‐(2‐chlorophenyl)ethan‐1‐one (10)**. CAS Number: 1387739‐82‐3. Yield: 50 %. Beige solid. R_*f*_: 0.56. M.p. 100–102 °C. ^1^H‐NMR (500 MHz, DMSO‐*d*_6_): (δ) 2.34 (m, 4H), 3.50 (m, 6H), 3.79 (s, 2H, CH_2_), 7.15 (d, *J=8.2*, 2H, ArH), 7.25–7.28 (m, 3H, ArH), 7.35 (m, 2H, ArH), 7.41 (m, 1H, ArH). ^13^C‐NMR (126 MHz, DMSO‐d_6_): (δ) 37.3, 41.3, 45.2, 52.2, 52.6, 60.8, 114.7, 115.2, 126.9, 128.3, 128.9, 130.7, 130.8, 131.8, 133.6, 133.9, 134.4, 158.9–163.7 (d, J_C–F_=242 Hz), 167.5. HRMS (ESI), *m/z*: calcd. for C_19_H_21_ClFN_2_O^+^: 347.1321 [M+H]^+^; found: 347.1340. Anal. Calcd for (C_19_H_20_ClFN_2_O): C 65.80, H 5.81, N 8.08. Found: C 65.40, H 5.61, N 7.89.

**1‐[4‐(4‐Fluorobenzyl)piperazin‐1‐yl]‐2‐(3‐chlorophenyl)ethan‐1‐one (11)**. Yield: 48 %. Yellow solid. R_*f*_: 0.53. M.p. 69–71 °C. ^1^H‐NMR (500 MHz, DMSO‐d_6_): (δ) 2.28 (m, 4H), 3.47 (m, 6H), 3.72 (s, 2H, CH2), 7.12–7.18 (m, 3H, ArH), 7.27–7.34 (m, 5H, ArH). ^13^C‐NMR (126 MHz, DMSO‐d_6_): (δ) 38.7, 41.3, 45.3, 52.1, 52.6, 60.8, 114.7, 115.1, 126.3, 127.9, 129.1, 129.9, 130.6, 130.8, 132.7, 133.9, 138.5, 158.9–163.7 (d, J_C–F_=242 Hz), 168.2. HRMS (ESI), m/z: calcd. for C_19_H_21_ClFN_2_O^+^: 347.1321 [M+H]^+^; found: 347.1307. Anal. Calcd for (C_19_H_20_ClFN_2_O): C 65.80, H 5.81, N 8.08. Found: C 65.44, H 5.66, N 7.96.

**1‐[4‐(4‐Fluorobenzyl)piperazin‐1‐yl]‐2‐(4‐chlorophenyl)ethan‐1‐one (12)**. CAS Number: 1329294‐83‐8. Yield: 54 %. White solid. R_*f*_: 0.52. M.p. 120–122 °C. ^1^H‐NMR (500 MHz, CDCl_*3*_): (δ) 2.25 (m, 2H), 2.39 (m, 2H), 3.42 (m, 4H), 3.64 (m, 2H), 3.67 (s, 2H, CH_2_), 6.99 (t, *J* **=**8.7 Hz, 2H, ArH), 7.16 (m, 2H, ArH), 7.24 (m, 2H, ArH), 7.28 (m, 2H, ArH). ^13^C‐NMR (126 MHz, CDCl_*3*_): (δ) 40.3, 41.9, 46.2, 52.7, 52.9, 62.1, 115.2, 115.4, 128.9, 130.2, 130.6, 130.7, 132.8, 133.6, 136.7, 161.3–163.2 (d, J_C–F_=241 Hz), 169.1. HRMS (ESI), *m/z*: calcd. for C_19_H_21_ClFN_2_O^+^: 347.1321 [M+H]^+^; found: 347.1306. Anal. Calcd for (C_19_H_20_ClFN_2_O): C 65.80, H 5.81, N 8.08. Found: C 65.66, H 5.55, N 8.28.

**1‐[4‐(4‐Fluorobenzyl)piperazin‐1‐yl]‐2‐(2‐bromophenyl)ethan‐1‐one (13)**. CAS Number: 1988172‐38‐8. Yield: 47 %. White solid. R_*f*_: 0.55. M.p. 113–115 °C. ^1^H‐NMR (500 MHz, CDCl_*3*_): (δ) 2.31 (m, 2H), 2.40 (m, 2H), 3.43 (m, 4H), 3.65 (m, 2H), 3.79 (s, 2H, CH_2_), 6.98 (m, 2H, ArH), 7.08–7.12 (m, 1H, ArH) 7.25 (m, 4H, ArH), 7.53 (m, 1H, ArH). ^13^C‐NMR (126 MHz, CDCl_*3*_): (δ) 40.7, 41.9, 46.1, 52.7, 52.9, 62.0, 115.0, 115.4, 124.6, 127.7, 128.6, 130.5, 130.7, 132.8, 133.4, 133.5, 135.2, 159.7–164.6 (d, J_C–F_=242 Hz), 168.6. HRMS (ESI), *m/z*: calcd. for C_19_H_21_BrFN_2_O^+^: 391.0816 [M+H]^+^; found: 391.0797. Anal. Calcd for (C_19_H_20_BrFN_2_O): C 58.32, H 5.15, N 7.16. Found: C 58.44, H 5.30, N 7.18

**1‐[4‐(4‐Fluorobenzyl)piperazin‐1‐yl]‐2‐(3‐bromophenyl)ethan‐1‐one (14)**. CAS Number: 1985981‐75‐6. Yield: 52 %. White solid. R_*f*_: 0.54. M.p. 240–242 °C. ^1^H‐NMR (500 MHz, DMSO‐*d_6_*): (δ) 2.93 (m, 2H), 3.14 (bs, 1H), 3.28 (m, 2H), 3.59 (bs, 1H), 3.77 (s, 2H, CH_2_), 4.14 (bs, 1H), 4.34 (m, 3H), 7.25 (m, 4H, ArH), 7.42 (d, 2H, *J=*8.6, ArH), 7.68 (m, 2H, ArH). ^13^C‐NMR (126 MHz, DMSO‐d_6_): (δ) 38.2, 40.8, 41.9, 49.9, 50.3, 57.4, 115.42, 115.8, 121.4, 125.8, 128.5, 129.3, 130.3, 132.2, 133.8, 133.9, 138.3, 160.2–165.1 (d, J_C–F_=245 Hz), 168.8. HRMS (ESI), *m/z*: calcd. for C_19_H_21_BrFN_2_O^+^: 391.0816 [M+H]^+^; found: 391.0807. Anal. Calcd for (C_19_H_20_BrFN_2_O): C 58.32, H 5.15, N 7.16. Found: C 58.56, H 5.40, N 7.31

**1‐[4‐(4‐Fluorobenzyl)piperazin‐1‐yl]‐2‐(4‐bromophenyl)ethan‐1‐one (15)**. CAS Number: 1146917‐07‐8. Yield: 51 %. Light yellow solid. R_*f*_: 0.52. M.p. 120–121 °C. ^1^H‐NMR (500 MHz, DMSO‐*d_6_*): (δ) 2.27 (m, 4H), 3.46 (m, 6H), 3.67 (s, 2H, CH_2_), 7.11–7.17 (m, 4H, ArH), 7.31 (m, 2H, ArH), 7.46–7.49 (m, 2H, ArH). ^13^C‐NMR (126 MHz, DMSO‐d_6_): (δ) 38.7, 41.3, 45.4, 52.2, 52.7, 60.9, 115.1, 119.6, 130.8, 131.1, 131.5, 134.0, 135.5, 160.4–162.4 (d, J_C–F_=248 Hz), 168.5. HRMS (ESI), *m/z*: calcd. for C_19_H_21_BrFN_2_O^+^: 391.0816 [M+H]^+^; found: 391.0804. Anal. Calcd for (C_19_H_20_BrFN_2_O): C 58.32, H 5.15, N 7.16. Found: C 57.99, H 4.95, N 7.26.

**1‐[4‐(4‐Fluorobenzyl)piperazin‐1‐yl]‐2‐(2‐nitrophenyl)ethan‐1‐one (16)**. Yield: 60 %. Yellow solid. M.p. 88–90 °C. R_*f*_: 0.52. ^1^H‐NMR (500 MHz, DMSO‐d_6_): (δ) 2.31 (m, 2H), 2.43 (m, 2H), 3.42 (m, 2H), 3.50 (s, 2H, CH2), 3.57 (m, 2H), 4.12 (s, 2H, CH2), 7.14–7.17 (m, 2H, ArH), 7.36 (m, 2H, ArH), 7.45 (m, 1H, ArH), 7.51–7.54 (m, 1H, ArH), 7.66 (m, 1H, ArH), 8.02 (m, 1H, ArH). ^13^C‐NMR (126 MHz, DMSO‐d_6_): (δ) 37.7, 41.9, 50.4, 50.8, 57.9, 115.7, 116.1, 124.7, 125.7, 128.4, 131.1, 133.5, 133.6, 149.1, 160.4–165.3 (d, J_C–F_=245 Hz), 167.6. HRMS (ESI), m/z: calcd. for C_19_H_21_FN_3_O_3_^+^: 358.1561 [M+H]^+^; found: 358.1546. Anal. Calcd for (C_19_H_20_FN_3_O_3_): C 63.86, H 5.64, N 11.76. Found: C 63.67, H 5.43, N 11.50.

**1‐[4‐(4‐Fluorobenzyl)piperazin‐1‐yl]‐2‐(3‐nitrophenyl)ethan‐1‐one (17)**. Yield: 53 %. Yellow solid. R_*f*_: 0.52. M.p. 83–84 °C. ^1^H‐NMR (500 MHz, DMSO‐*d_6_*): (δ) 2.32 (m, 4H), 3.49 (m, 6H), 3.90 (s, 2H, CH_2_), 7.13–7.17 (m, 2H, ArH), 7.32 (d, *J=5.6*, 2H, ArH), 7.58–7.61 (m, 1H, ArH), 7.66 (d, *J=7.5*, 1H, ArH), 8.10 (s, 2H, ArH). ^13^C‐NMR (126 MHz, DMSO‐d_6_): (δ) 38.4, 41.3, 45.3, 52.1, 52.6, 60.8, 114.9, 115.1, 121.4, 121.7, 124.2, 129.6, 130.9, 134.8, 136.5, 138.5, 147.6, 162.4, 168.2. HRMS (ESI), *m/z*: calcd. for C_19_H_21_FN_3_O_3_^+^: 358.1561 [M+H]^+^; found: 358.1537. Anal. Calcd for (C_19_H_20_FN_3_O_3_): C 63.86, H 5.64, N 11.76. Found: C 63.55, H 5.44, N 11.51.

**1‐[4‐(4‐Fluorobenzyl)piperazin‐1‐yl]‐2‐(4‐nitrophenyl)ethan‐1‐one (18)**. Yield: 49 %. Orange solid. R_*f*_ : 0.52. M.p. 98–100 °C. ^1^H‐NMR (500 MHz, DMSO‐*d_6_*): (δ) 2.31 (m, 4H), 3.50 (m, 6H), 3.88 (s, 2H, CH_2_), 7.14 (m, 2H, ArH), 7.33 (m, 2H, ArH), 7.48 (m, 2H, ArH), 8.16 (m, 2H, ArH). ^13^C‐NMR (126 MHz, DMSO‐d_6_): (δ) 41.3, 45.3, 52.0, 52.6, 60.8, 114.7, 115.1, 123.2, 130.6, 130.7, 130.8, 133.8, 133.9, 144.3, 146.2, 158.9–163.7 (d, J_C‐F_=242 Hz), 167.8. HRMS (ESI), *m/z*: calcd. for C_19_H_21_FN_3_O_3_^+^: 358.1561 [M+H]^+^; found: 358.1501. Anal Calcd for (C_19_H_20_FN_3_O_3_): C 63.86, H 5.64, N 11.76. Found: C 63.99, H 6.00, N 12.00.

**1‐[4‐(4‐Fluorobenzyl)piperazin‐1‐yl]‐2‐(4‐methoxyphenyl)ethan‐1‐one (19)**. CAS Number: 1796840‐99‐7. Yield: 52 %. White solid. R_*f*_: 0.45. M.p. 234–238 °C. ^1^H‐NMR (500 MHz, DMSO‐*d*_6_): (δ) 2.95 (bs, 4H), 3.67 (m, 2H), 3.73 (s, 3H, CH_3_), 4.17 (bs, 2H), 4.4 (m, 4H), 6.85–6.88 (m, 2H, ArH), 7.11–7.14 (m, 2H, ArH),7.33 (m, 2H, ArH), 7.58 (bs, 2H, ArH). ^13^C‐NMR (126 MHz, DMSO‐d_6_): (δ) 40.8, 42.2, 50.3, 50.7, 55.0, 57.9, 113.8, 115.7, 116.1, 125.6, 127.1, 130.1, 133.7, 133.8, 157.9, 160.4–165.3 (d, J_C–F_=246 Hz), 169.5. HRMS (ESI), *m/z*: calcd. for C_20_H_24_FN_2_O_2_^+^: 343.1816 [M+H]^+^; found: 343.1766. Anal. Calcd for (C_20_H_23_FN_2_O_2_): C 70.16, H 6.77, N 8.18. Found: C 70.33, H 6.98, N 8.00.

#### General procedure for the synthesis of aminophenyl derivatives 20–22

A solution of nitro‐derivatives 16–18 (150 mg, 0.42 mmol) in ethanol (EtOH, 15 mL) was prepared; then we added Pd/C as catalyst and hydrazine (134 μL, 4.2 mmol). The resulting mixture was heated at reflux for 2 hours. After cooling, the catalyst was removed by filtration on celite and treated many times with EtOAc. The organic phase was then extracted with EtOAc/H_2_O (3×15 mL), dried with Na_2_SO_4_ and the solvent was removed in vacuo. The final products were purified by flash chromatography (DCM/MeOH 98 : 2).

**1‐[4‐(4‐Fluorobenzyl)piperazin‐1‐yl]‐2‐(2‐aminophenyl)ethan‐1‐one (20)**. Yield: 80 %. Oily residue. R_*f*_: 0.32. ^1^H‐NMR (500 MHz, DMSO‐*d*_6_): (δ) 2.26 (m, 4H), 3.43 (bs, 2H), 3.47 (m, 4H), 3.49 (s, 2H, CH_2_), 5.04 (bs, 2H, NH_2_), 6.50 (m, 1H, ArH), 6.63 (m, 1H, ArH), 6.93 (m, 2H, ArH), 7.13 (m, 2H, ArH) 7.31 (m, 2H, ArH). ^13^C‐NMR (126 MHz, CDCl_3_): (δ) 38.1, 42.1, 46.5, 52.7, 52.9, 62.1, 115.2, 115.4, 116.5, 118.4, 119.7, 128.3, 130.5, 130.6, 130.7, 133.5, 146.6, 161.2–163.2 (J_C–F_=244 Hz), 169.7. HRMS (ESI), *m/z*: calcd. for C_19_H_23_FN_3_O^+^: 328.1820 [M+H]^+^; found: 328.1779. Anal. Calcd for (C_19_H_22_FN_3_O): C 69.70, H 6.77, N 12.83. Found: C 69.88, H 6.72, N 12.71.

**1‐[4‐(4‐Fluorobenzyl)piperazin‐1‐yl]‐2‐(3‐aminophenyl)ethan‐1‐one (21)**. Yield: 72 %. Oily residue. R_*f*_: 0.23. ^1^H‐NMR (500 MHz, DMSO‐*d_6_*): (δ) 2.22 (m, 2H), 2.27 (m, 2H), 3.43 (m, 6H), 3.51 (s, 2H, CH_2_), 5.00 (bs, 2H, NH_2_), 6.32 (d, *J=7.9*, 1H, ArH), 6.40 (m, 2H, ArH), 6.91 (m, 1H, ArH), 7.13 (m, 2H, ArH), 7.31 (m, 2H, ArH). ^13^C‐NMR (126 MHz, CDCl_3_): (δ) 41.2, 41.8, 46.2, 52.7, 52.9, 62.0, 113.7, 115.1, 115.1, 115.3, 118.8, 129.7, 130.6, 130.6, 133.5, 133.5, 136.2, 146.9, 161.2–163.1 (d, J_C–F_=244 Hz), 169.7. HRMS (ESI), *m/z*: calcd. for C_19_H_23_FN_3_O^+^: 328.1820 [M+H]^+^; found: 328.1824. Anal. Calcd for (C_19_H_22_FN_3_O): C 69.70, H 6.77, N 12.83. Found: C 69.77, H 6.91, N 13.00.

**1‐[4‐(4‐Fluorobenzyl)piperazin‐1‐yl]‐2‐(4‐aminophenyl)ethan‐1‐one (22)**. Yield: 92 %. Beige solid. R_*f*_: 0.26. M.p: 116.5–118.5 °C. ^1^H‐NMR (500 MHz, DMSO‐*d_6_*): (δ) 2.20 (m, 2H), 2.25 (m, 2H), 3.42 (m, 6H), 3.47 (s, 2H, CH_2_), 4.90 (bs, 2H, NH_2_), 6.48 (m, 2H, ArH), 6.84 (m, 2H, ArH), 7.10–7.15 (m, 2H, ArH), 7.29–7.33 (m, 2H, ArH). ^13^C‐NMR (126 MHz, DMSO‐d_6_): (δ) 41.1, 45.5, 52.1, 52.6, 60.8, 113.9, 114.7, 115.1, 122.4, 129.1, 130.6, 130.8, 134.0, 146.9, 158.9–163.7 (d, J_C–F_=242 Hz), 169.4. HRMS (ESI), *m/z*: calcd. for C_19_H_23_FN_3_O^+^: 328.1820 [M+H]^+^; found: 328.1819. Anal. Calcd for (C_19_H_22_FN_3_O): C 69.70, H 6.77, N 12.83. Found: C 69.41, H 6.51, N 12.55.

#### General procedure for the synthesis of 1‐[4‐(4‐fluorobenzyl)piperazin‐1‐yl](phenyl)methanone derivatives (23–25)

To a solution of the amine 6 (200 mg, 1.03 mmol) in dichloromethane (DCM, 2 mL), a mixture of the N,N‐diisopropylethylamine (DIPEA, 268μL, 1.54 mmol) and suitable acyl chloride (1.13 mmol) was added dropwise. The reaction was stirred at room temperature for 5 hours and turn off by addition of MeOH (2 mL). Then, we added water (10 mL) and the mixture was extracted with DCM (3x10 mL). The organic phase was dried Na_2_SO_4_; the solvent was removed under reduced pressure; the final products were purified by flash chromatography (DCM/MeOH 96 : 4). The ^13^C‐NMR spectroscopic measurements were registered for the corresponding hydrochlorides that we prepared by treatment of compounds 23–25 with a solution of HCl.

**1‐[4‐(4‐Fluorobenzyl)piperazin‐1‐yl]‐[3‐fluoro‐2‐(trifluoromethyl)phenyl]methanone (23)**. Yield 96 %. Oily residue. R_*f*_: 0.62. ^1^H‐NMR (500 MHz, CDCl_3_): (δ) 2.29–2.39 (m, 2H), 2.47–2.55 (m, 2H), 3.18 (t, J**=**5.1 Hz, 2H), 3.50 (s, 2H, CH_2_), 3.75–3.83 (m, 2H), 6.97–7.03 (m, 2H, ArH), 7.08 (d, *J* **=**7.7 Hz, 1H, ArH), 7.25 (m, 3H, ArH), 7.55–7.59 (m, 1H, ArH). As hydrochloride ^13^C‐NMR (126 MHz, DMSO‐d_6_): (δ) 42.9, 49.7, 50.2, 57.3, 57.9, 115.5, 115.8, 117.9, 118.1, 123.5, 125.6, 133.9, 135.7, 135.8, 135.9, 158.2, 160.7, 161.5, 163.9, 164.9, 165.2. Anal. Calcd for: C_19_H_17_F_5_N_2_O: C 59.38, H 4.46, N 7.29. Found: C 59.29, H 4.49, N 7.01.

**1‐[4‐(4‐Fluorobenzyl)piperazin‐1‐yl]‐[4‐fluoro‐2‐(trifluoromethyl)phenyl]methanone (24)**. Yield 91 %. Oily residue. R_*f*_: 0.64. ^1^H‐NMR (500 MHz, CDCl_3_): (δ) 2.35 (m, 2H), 2.54 (t, J=5.2 Hz, 2H), 3.19 (t, J**=**5.1 Hz, 2H), 3.52 (s, 2H, CH_2_), 3.82 (m, 2H), 6.98–7.02 (m, 2H, ArH), 7.30 (m, 4H, ArH), 7.39–7.42 (m, 1H, ArH). Hydrochloride ^13^C‐NMR (126 MHz, DMSO‐d_6_): (δ) 43.2, 49.7, 57.9, 114.2, 114.7, 115.4, 115.9, 119.8, 120.3, 125.7, 130.2, 130.5, 133.8, 133.9, 159.3, 160.3, 164.3, 165.2, 165.4. Anal. Calcd for: C_19_H_17_F_5_N_2_O: C 59.38, H 4.46, N 7.29. Found: C 59.42, H 4.53, N 7.05.

**1‐[4‐(4‐Fluorobenzyl)piperazin‐1‐yl]‐[5‐fluoro‐2‐(trifluoromethyl)phenyl]methanone (25)**. Yield 94 %. Oily residue. R_*f*_: 0.65. ^1^H‐NMR (500 MHz, CDCl_3_): (δ) 2.28–239 (m, 2H), 2.51 (m, 2H), 3.18 (t, J=5.1 Hz, 2H), 3.49 (s, 2H, CH_2_), 3.80 (m, 2H), 6.98–7.04 (m, 3H, ArH), 7.19 (dddd, J**=**7.9, 3.4, 1.7, 0.8 Hz, 1H, ArH), 7.27 (m, 2H, ArH), 7.70 (m, 1H, ArH). Hydrochloride ^13^C‐NMR (126 MHz, DMSO‐d_6_): (δ) 43.1, 49.7, 58.0, 114.9, 115.4, 115.9, 116.8, 117.2, 120.7, 121.3, 125.72, 129.8, 133.8, 134.0, 136.6, 160.3, 161.4, 164.6, 165.2, 166.4. Anal. Calcd for: C_19_H_17_F_5_N_2_O: C 59.38, H 4.46, N 7.29. Found: C 59.30, H 4.61, N 7.04.

#### General procedure for the synthesis of [4‐(4‐Fluorobenzyl)piperazin‐1‐yl](phenyl)methanone derivatives (26–32)

We prepared a mixture by combining a solution of the amine 6 (200 mg, 1.03 mmol) in triethylamine (TEA, 153 μL, 1.13 mmol) with solution of the suitable carboxylic acid derivative (1.13 mmol), N,N,N’,N’‐tetramethyl‐O‐(1Hbenzotriazol‐1‐yl)uronium hexafluorophosphate (HBTU, 428 mg, 1.13 mmol) in *N*,*N*‐Dimethylformamide (DMF, 4 mL). The reaction was carried out for 10 min at 50 °C in microwave conditions, then the mixture was quenched with water (10 mL) and extracted with ethyl acetate (EtOAc, 3x10 mL). The collected organic phases were treated with brine (3x10 mL), dried with Na_2_SO_4_ and, finally, the solvent was removed in vacuo. The crude residues were purified by treatment with Et_2_O or Et_2_O/EtOH, or by chromatography (DCM/MeOH 96 : 4), thus giving desired molecules. For compounds 27 and 32 CAS numbers have been already registered but due to the lack of details in terms of synthetic procedures, chemical properties and structural characterization, we reported below this information.

**1‐[4‐(4‐Fluorobenzyl)piperazin‐1‐yl]‐(3‐chloro‐2‐nitro‐phenyl)methanone (26)**. Yield: 34 %. Brown solid. R_*f*_: 0.64. M.p. 91–92 °C. ^1^H‐NMR (500 MHz, DMSO‐d_6_): (δ) 2.38 (m, 4H), 3.29 (m, 2H), 3.50 (s, 2H, CH_2_), 3.58 (bs, 2H), 7.14 (m, 2H, ArH), 7.34 (dd, J=8.2, J=5.8, 2H, ArH), 7.56 (d, J=7.6, 1H, ArH), 7.72 (m, 1H, ArH), 7.84 (d, J=7.6, 1H, ArH). ^13^C‐NMR (126 MHz, DMSO‐d_6_): (δ) 41.6, 46.9, 51.7, 52.6, 60.7, 114.7, 114.9, 124.9, 126.5, 126.8, 130.5, 130.9, 132.1, 133.2, 133.8, 147.3, 160.4–162.3 (d, J_C–F_=240 Hz), 163.3. HRMS (ESI), *m/z*: calcd. for C_18_H_18_ClFN_3_O_3_^+^: 378.1015 [M+H]^+^; found: 378.0979. Anal. Calcd for (C_18_H_17_ClFN_3_O_3_): C 57.23, H 4.54, N 11.12. Found: C 57.27, H 4.51, N 11.20.

**1‐[4‐(4‐Fluorobenzyl)piperazin‐1‐yl]‐(4‐chloro‐2‐nitro‐phenyl)methanone (27)**. CAS Number: 2344352‐87‐8. Yield: 37 %. Orange solid. R_*f*_: 0.64. M.p. 141–142 °C. ^1^H‐NMR (500 MHz, DMSO‐d_6_): (δ) 2.31 (bs, 2H), 2.44 (bs, 2H), 3.19 (bs, 2H), 3.49 (s, 2H, CH_2_), 3.63 (bs, 2H), 7.14 (m, 2H, ArH), 7.34 (dd, J=8.3, J=5.8, 2H, ArH), 7.57 (d, J=8.2, 1H, ArH), 7.92 (dd, J=8.2, J=2.0, 1H, ArH), 8.27 (m, 1H, ArH). ^13^C‐NMR (126 MHz, DMSO‐d_6_): (δ) 41.3, 46.5, 51.6, 52.1, 60.8, 114.7, 114.9, 124.7, 129.5, 130.5, 130.8, 131.0, 134.2, 134.5, 134.7, 146.1, 160.3–162.2 (d, J_C–F_=244 Hz), 164.2. HRMS (ESI), *m/z*: calcd. for C_18_H_18_ClFN_3_O_3_^+^: 378.1015 [M+H]^+^; found: 378.0973. Anal. Calcd for (C_18_H_17_ClFN_3_O_3_): C 57.23, H 4.54, N 11.12. Found: C 57.20, H 4.50, N 11.25.

**1‐[4‐(4‐Fluorobenzyl)piperazin‐1‐yl]‐(5‐chloro‐2‐nitro‐phenyl)methanone (28)**. Yield: 47 %. Orange solid. R_*f*_: 0.72. M.p. 130–131 °C. ^1^H‐NMR (500 MHz, CDCl_3_): (δ) 2.39 (m, 2H), 2.58 (m, 2H), 3.23 (m, 2H), 3.52 (s, 2H, CH2), 3.83 (m, 2H), 7.00 (m, 2H, ArH), 7.28 (m, 2H, ArH), 7.37 (d, J=2.26, 1H, ArH), 7.52 (dd, J=8.8, J=2.3, 1H, ArH), 8.15 (d, J=8.8, 1H, ArH). ^13^C‐NMR (126 MHz, DMSO‐d_6_): (δ) 41.3, 46.4, 51.5, 52.0, 60.8, 114.9, 115.1, 126.8, 127.7, 127.9, 130.0, 130.3, 130.5, 130.9, 133.8, 134.1, 139.7, 143.8, 160.3–162.3 (d, J_C–F_=242 Hz), 163.7. HRMS (ESI), m/z: calcd. for C_18_H_18_ClFN_3_O_3_^+^: 378.1015 [M+H]^+^; found: 378.0973. Anal. Calcd for (C_18_H_17_ClFN_3_O_3_): C 57.23, H 4.54, N 11.12. Found: C 57.21, H 4.58, N 11.20.

**1‐[4‐(4‐Fluorobenzyl)piperazin‐1‐yl]‐(2‐chloro‐6‐nitro‐phenyl)methanone (29)**. Yield: 64 %. Orange solid. R_*f*_: 0.71. M.p. 112–113 °C. ^1^H‐NMR (500 MHz, DMSO‐d_6_): (δ) 2.35 (m, 2H), 2.45 (m, 2H), 3.18 (m, 2H), 3.50 (s, 2H, CH2), 3.60 (m, 1H), 3.68 (m, 1H), 7.14 (m, 2H, ArH), 7.35 (m, 2H, ArH), 7.71 (m, 1H, ArH), 7.98 (dd, J=8.1, J=1.0, 1H, ArH), 8.20 (dd, J=8.3, J=1.0, 1H, ArH). ^13^C‐NMR (126 MHz, DMSO‐d_6_): (δ) 41.2, 45.7, 51.4, 52.1, 60.8, 114.9, 115.1, 123.9, 123.9, 130.8, 130.8, 130.9, 131.1, 131.2, 133.9, 135.6, 135.7, 146.2, 160.3–162.2 (d, J_C–F_=241 Hz), 161.9. HRMS (ESI), m/z: calcd. for C_18_H_18_ClFN_3_O_3_^+^: 378.1015 [M+H]^+^; found: 378.0971. Anal. Calcd for (C_18_H_17_Cl_F_N_3_O_3_): C 57.23, H 4.54, N 11.12. Found: C 57.14, H 4.40, N 11.25.

**1‐[4‐(4‐Fluorobenzyl)piperazin‐1‐yl]‐(3‐methoxy‐2‐nitro‐phenyl)methanone (30)**. Yield: 46 %. White solid. R_*f*_: 0.57. M.p. 108–109 °C. ^1^H‐NMR (500 MHz, CDCl_3_): (δ) 2.05 (m, 2H), 2.71 (m, 4H), 3.47 (s, 2H, CH2), 3.75 (m, 2H), 3.94 (s, 3H, OCH3), 6.93 (m, 1H, ArH), 7.04 (m, 2H, ArH) 7.11 (m, 1H, ArH), 7.35 (m, 2H, ArH), 7.51 (m, 1H, ArH). ^13^C‐NMR (126 MHz, DMSO‐d_6_): (δ) 44.9, 46.0, 50.6, 56.8, 57.0, 72.6, 115.2, 115.3, 118.3, 118.6, 125.7, 132.9, 133.2, 137.8, 141.4, 145.9, 151.2, 159.3, 164.4. HRMS (ESI), m/z: calcd. for C_19_H_21_FN_3_O_4_^+^: 374.1511 [M+H]^+^; found: 374.1471. Anal. Calcd for (C_19_H_20_FN_3_O_4_): C 61.12, H 5.40, N 11.25. Found: C 61.06, H 5.33, N 11.00.

**1‐[4‐(4‐Fluorobenzyl)piperazin‐1‐yl]‐(4‐methoxy‐2‐nitro‐phenyl)methanone (31)**. Yield: 45 %. Beige solid. R_*f*_: 0.54. M.p. 133–134 °C. ^1^H‐NMR (500 MHz, DMSO‐*d_6_*): (δ) 2.31 (bs, 2H), 2.43 (bs, 2H), 3.19 (m, 2H), 3.49 (s, 2H, CH_2_), 3.61 (bs, 2H), 3.88 (s, 3H, OCH_3_), 7.14 (m, 2H, ArH), 7.33–7.43 (m, 4H, ArH), 7.65 (s, 1H, ArH). ^13^C‐NMR (126 MHz, DMSO‐d_6_): (δ) 46.5, 51.7, 52.2, 56.1, 56.3, 60.8, 109.5, 114.9, 115.1, 120.4, 124.4, 129.0, 129.4, 130.5, 130.8, 133.9, 146.7, 159.7, 165.2. HRMS (ESI), *m/z*: calcd. for C_19_H_21_FN_3_O_4_^+^: 374.1511 [M+H]^+^; found: 374.1469. Anal. Calcd for (C_19_H_20_FN_3_O_4_): C 61.12, H 5.40, N 11.25. Found: C 61.10, H 5.67, N 11.01.

**1‐[4‐(4‐Fluorobenzyl)piperazin‐1‐yl]‐(5‐methoxy‐2‐nitro‐phenyl)methanone (32)**. CAS Number: 2345032‐69‐9. Yield: 49 %. White solid. R_*f*_: 0.58. M.p. 151–152 °C. ^1^H‐NMR (500 MHz, CDCl_3_): (δ) 2.35 (m, 2H), 2.51 (m, 1H), 2.63 (m, 1H), 3.20 (m, 2H), 3.50 (s, 2H, CH2), 3.76 (m, 2H), 3.91 (s, 3H, OCH3), 6.80 (m, 1H, ArH), 6.95–7.01 (m, 3H, ArH), 7.28 (m, 2H, ArH), 8.20 (d, J=9.2, 1H, ArH). ^13^C‐NMR (126 MHz, DMSO‐d_6_): (δ) 44.6, 46.3, 52.1, 56.4, 56.6, 60.7, 103.9, 114.8, 115.1, 116.3, 124.9, 127.4, 130.8, 130.9, 133.9, 137.8, 159.4, 161.9, 163.9. HRMS (ESI), m/z: calcd. for C_19_H_21_FN_3_O_4_^+^: 374.1511 [M+H]^+^; found: 374.1469. Anal. Calcd for (C_19_H_20_FN_3_O_4_): C 61.12, H 5.40, N 11.25. Found: C 61.25, H 5.44, N 11.30.

### Mushroom tyrosinase inhibition assay

Mushroom tyrosinase (EC 1.14.18.1) was purchased from Sigma Chemical Co. (St. Louis, MO, USA). Tyrosinase inhibition was assayed as previously described [14]. Briefly, aliquots (0.05 mL) of test compound at various concentrations (0.10–50 μM) were firstly mixed with 0.5 mL of L‐DOPA solution (1.25 mM), 0.9 mL of phosphate buffer (0.05 M, pH 6.8). The reaction mixture was preincubated at 25 °C for 10 min. Subsequently, 0.05 mL of an aqueous solution of mushroom tyrosinase (333 U/mL) was added to this mixture. Afterward, the linear increase in absorbance (Abs) was immediately recorded up to 5 minutes at 475 nm. The inhibitory activity was expressed as inhibition percentage respect to control sample. The concentrations leading to 50 % activity loss (IC_50_) were also calculated by interpolation of the dose‐response curves. Kojic acid [5‐hydroxy‐2‐(hydroxymethyl)‐4H‐pyran‐4‐one], a fungal secondary metabolite used as skin whitening agent, was employed as a positive standard (8–32 μM). A spectrophotometer (Shimadzu UV‐1601) was used for absorbance measurements.

### Kinetic analysis of the tyrosinase inhibition

A series of experiments were performed to determine the mode of inhibition of compounds **23**, **25**, **26** on tyrosinase. The reaction mixture consisted of four different concentrations of the substrate L‐DOPA (0.6–5 mM), and mushroom tyrosinase in phosphate buffer (0.05 M, pH 6.8). In addition, three different concentrations of compounds **23**, **25**, **26** (0.2–0.4–0.8 μM) were used for the analysis. Preincubation and measurement time were the same as discussed in mushroom tyrosinase inhibition assay. The Michaelis‐Menten constant (Km) and maximal velocity (Vmax) of tyrosinase were determined by Lineweaver‐Burk plots.

### Computational studies

#### Molecular docking on mushroom tyrosinase

Docking studies were performed by Gold software V 2020.2.0^[21]^ using the crystal structure of AbTYR in complex with the inhibitor tropolone retrieved from the RCSB Protein Data Bank (PDB code 2y9X).^[8]^ Protocols used for docking were described in our previous papers.[^13^, ^14^]

#### Homology modelling of human tyrosinase

The 3D structure of human tyrosinase was built by homology modelling by means of SWISS‐MODEL.^[22]^ The primary sequence of hTYR was retrieved from the UniProt database (UniProt code P14679) and provided as input for the template search. The top‐ranked template corresponding to the chain A of crystal structure of human TYRP‐1 mutant (T391V‐R374S‐Y362F) in complex with kojic acid (PDB code 5 M8Q) and showing a sequence similarity of 0.43 % was chosen to build the model. The TRP‐1 protein structure used as template contains two Zn ions that were replaced with Cu ions in the obtained hTYR homology model. The residues of the resulting protein were protonated at physiological pH by H++ web server.^[25]^ The structure was then energy minimized by Amber18 software^[26]^ as follow. Initially, the system was solvated in a cubic box of TIP3P water model and neutralized by adding Na+ and Cl− ions setting a salt concentration of 0.15 M. The system was then optimized by 500 steps of steepest descent followed by 500 steps of conjugate gradient minimization. During the calculations, copper ions were kept fixed and distance restraints were applied between the copper ions and the nitrogen atoms of the histidine residues involved in metals coordination. The protein was parametrized by employing the ff14SB force field.^[27]^ The water molecules and the counterions were removed to the resulting optimized structure. Ramachandran plot analysis was conducted for the so obtained model by means of Procheck tool V 3.5.^[28]^ As displayed in Figure S1 (see Supporting Information), the generated hTYR homology model presents the 84.5 % of the residues in the most favored regions, the remaining 15,5 % in other allowed regions and no residues were found in disallowed regions.

#### Molecular docking on human tyrosinase

The obtained homology model of hTYR was exploited to perform docking studies by means of the software Gold 2020.2.0. The ligands were docked into hTYR active site by applying the same protocol described above for AbTYR. The side chains of the amino acid residue of the binding site Lys306, Lys334, Phe347, Ser360, Asn364, Ile368 and Val377 were set as flexible. The top scored docking poses were chosen for the analysis and representation. The putative ligand‐protein contacts were analyzed by Discovery Studio Visualizer V20.

### Cell culture

Murine melanoma B16F10 cells (CRL‐6475) were from the American Type Culture Collection (ATCC, Manassas, VA, USA). Cells were cultured as previous reported.^[14]^

### MTT assay for cell viability

The viability of B16F10 cells was detected by the colorimetric 3‐(4,5‐dimethylthiazol‐2‐yl)‐2,5‐diphenyltetrazolium bromide (MTT) assay. A density of 5x103 cells/well, in the absence or presence of compounds (ranging from 1 μM to 100 μM), were seeded in 96‐well plates. The cells were incubated for 48 h at the temperature of 37 °C. MTT solution (0.5 mg/mL) was added at the end of incubation time. The medium was removed after 3 h of incubation at 37 °C. Then, DMSO was added to the precipitates and the absorbance was determined at 560 nm using a microplate reader (Multiskan FC – Thermo Scientific). The cell viability was expressed as the percentage of the amount of living cells in treated samples relative to untreated controls (100 % viability). The mean value and standard deviation (SD) were calculated from triplicate experiments.

### Tyrosinase zymography (L‐DOPA staining)

The tyrosinase activity was performed by DOPA‐staining assay as previously reported but with slight modifications.^[29]^ A day after seeding the B16F10 cells (6X104 cells/mL) the culture medium was changed by the same medium supplemented with either α‐MSH alone or α‐MSH plus compound (0–100 μM)) or kojic acid (100 μM). After incubation for 48 h, cells were washed and lysed using phosphate buffer (50 mM, pH 6.8) containing Triton X‐100 (1 %) and phenylmethyl‐sulfonyl fluoride (0.1 mM). Cellular lysates were centrifugated at 12000 g for 20 min at 4 °C. Bradford method was used to determine the protein content using BSA as standard. Total proteins (5 μg) were mixed with Tris‐HCl buffer (10 mM, pH 7.0), containing SDS (1 %) and resolved by 8 % SDS‐polyacrylamide gel electrophoresis. After running, gel was rinsed in phosphate buffer (0.1 M, pH 6.8) and equilibrated for 30 min twice. Then, staining solution containing phosphate buffer (0.1 M, pH 6.8) and L‐DOPA (5 mM) was added to the gel. After 1 h incubation in the dark at 37 °C, dark melanin‐containing bands were visualized in the gel, as the result of tyrosinase activity.

## Conflict of interest

The authors declare no conflict of interest.

## Supporting information

As a service to our authors and readers, this journal provides supporting information supplied by the authors. Such materials are peer reviewed and may be re‐organized for online delivery, but are not copy‐edited or typeset. Technical support issues arising from supporting information (other than missing files) should be addressed to the authors.

Supporting Information
