# Supplementary material for: Evaluation of 4‐(4‐Fluorobenzyl)piperazin‐1‐yl]‐Based Compounds as Competitive Tyrosinase Inhibitors Endowed with Antimelanogenic Effects
Source: ChemMedChem. 2021 Jul 26;16(19):3083–93. doi: 10.1002/cmdc.202100396 (PMC8518915; doi:10.1002/cmdc.202100396)
Supplement: Supplementary file 1 — Supporting Information [file CMDC-16-3083-s001.pdf]

ChemMedChem

Supporting Information

Evaluation of 4-(4-Fluorobenzyl)piperazin-1-yl]-Based Compounds as Competitive Tyrosinase Inhibitors Endowed with Antimelanogenic Effects

Salvatore Mirabile, Serena Vittorio, Maria Paola Germanò, Ilenia Adornato, Laura Ielo, Antonio Rapisarda, Rosaria Gitto, Francesca Pintus, Antonella Fais, and Laura De Luca*

Supporting Information:

Content:

Figure S1: Ramachandran plot of hTYR homology model……………………………………………….S2

Figure S2-S42: ^{1}H-NMR and selected representative ^{13}C-NMR spectra…………………………………S3

Figure S43: Representation of AbTYR binding site………………….…………….…………………...S24

Figure S44: Representation of hTYR binding site ………..……………………………………………….S25

Figure S45: Sequence alignment of hTYR and (TYRP-1) mutant (T391V-R374S-Y362F)………………S26

Figure S46: B16F10 melanoma cell viability after treatment with compounds **23**, **25** and **26**…………… S27

Table S1: CAS numbers and smiles for compounds **7-32**..…………………………….……..…………S28

Table S2: Selected physicochemical parameters and predicted drug-likeness properties.……..………..…S29

Figure S1. Ramachandran plot of hTYR homology model

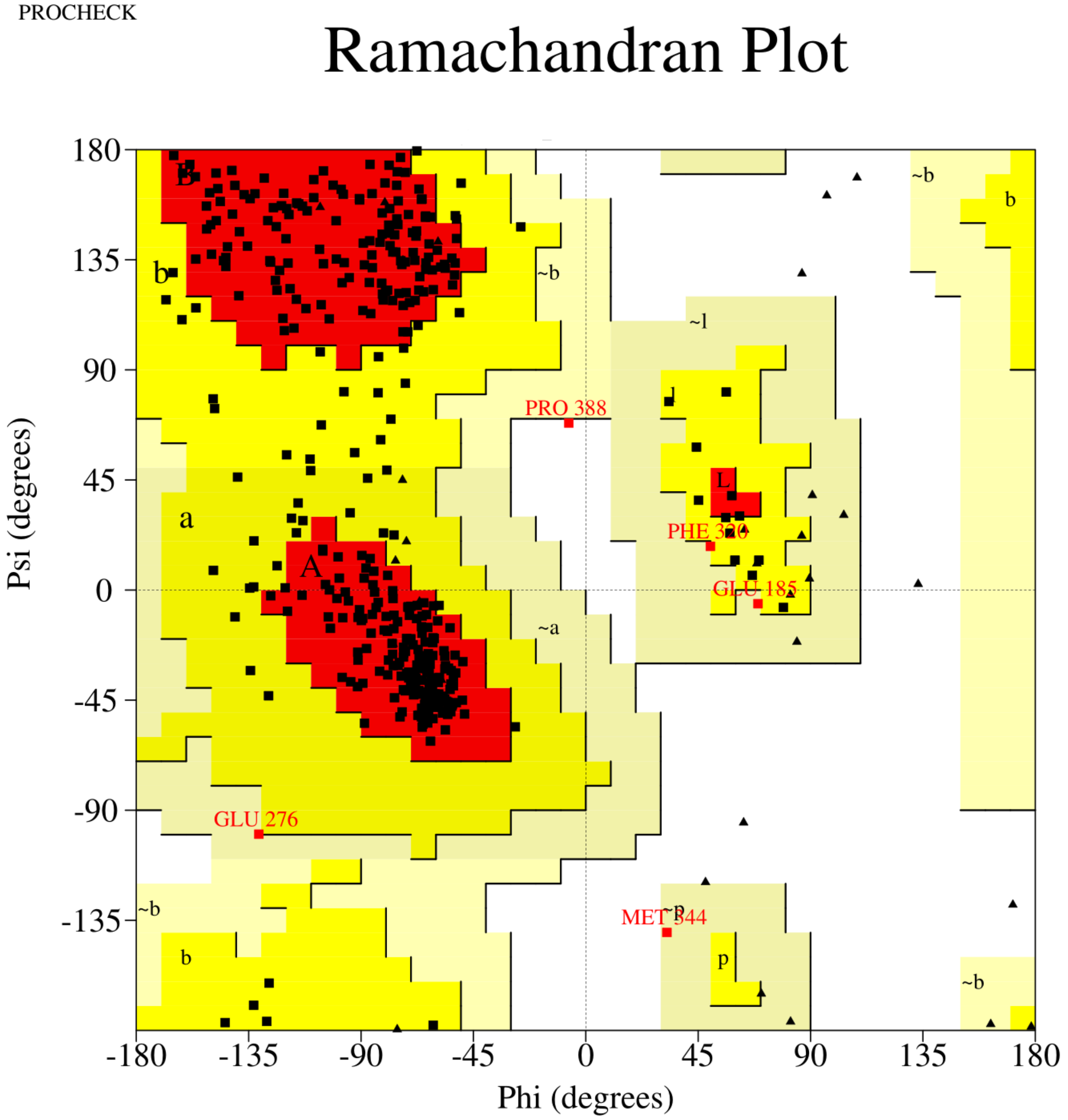

Plot statistics

Residues in most favoured regions [A,B,L]	316	84.5%
Residues in additional allowed regions [a,b,l,p]	54	14.4%
Residues in generously allowed regions [~a,~b,~l,~p]	4	1.1%
Residues in disallowed regions	0	.0%
	----	------
Number of non-glycine and non-proline residues	374	100.0%
Number of end-residues (excl. Gly and Pro)	2	
Number of glycine residues (shown as triangles)	30	
Number of proline residues	30	

Total number of residues	436	

^{1}H-NMR and selected representative ^{13}C-NMR spectra

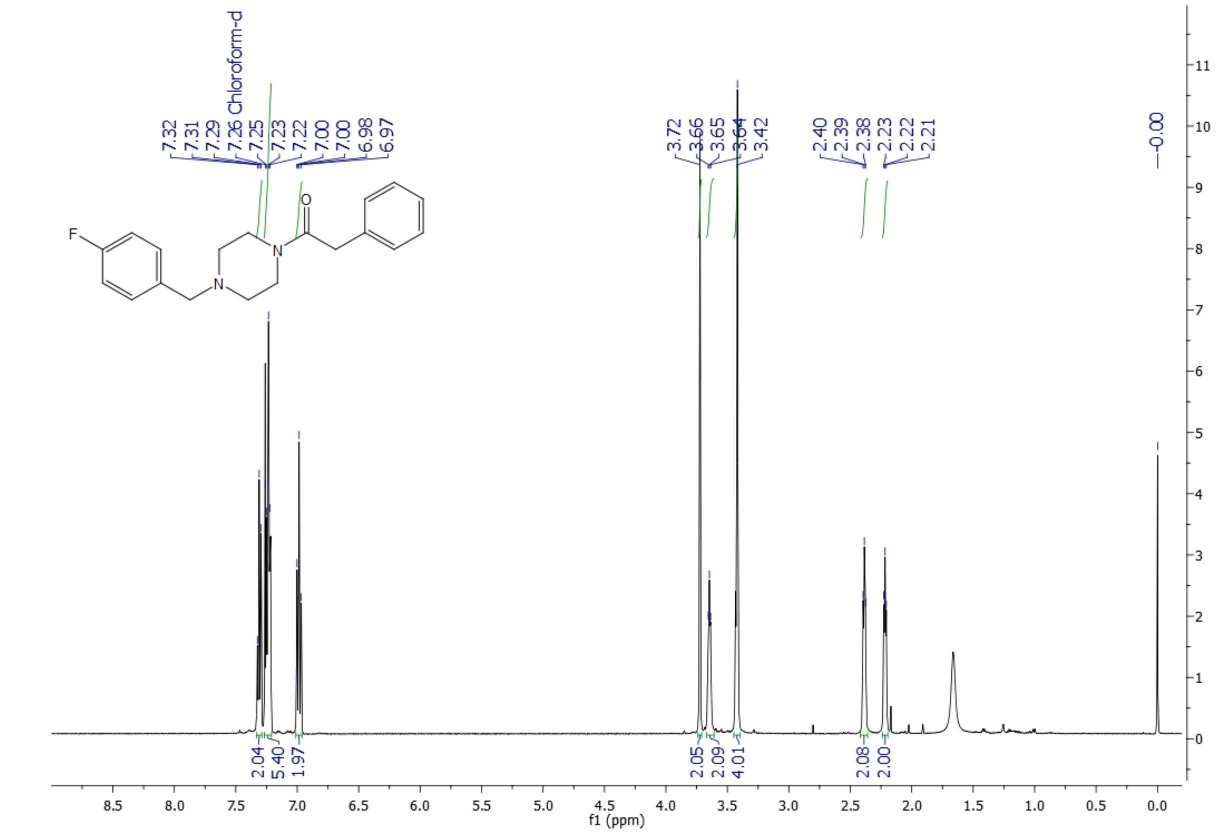

Figure S2: ^{1}H-NMR ($CDCl_3$) spectrum of 1-[4-(4-Fluorobenzyl)piperazin-1-yl]-2-phenylethan-1-one (**7**)

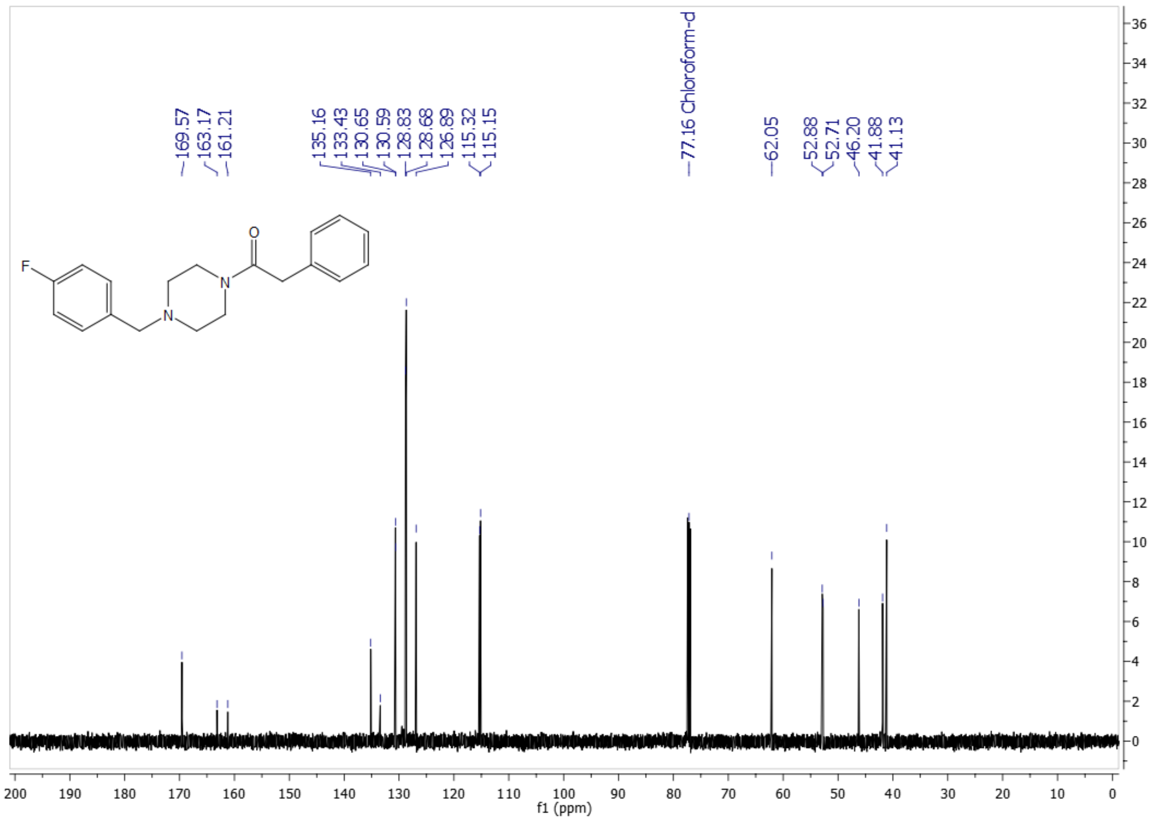

Figure S3: ^{13}C-NMR ($CDCl_3$) spectrum of 1-[4-(4-Fluorobenzyl)piperazin-1-yl]-2-phenylethan-1-one (**7**)

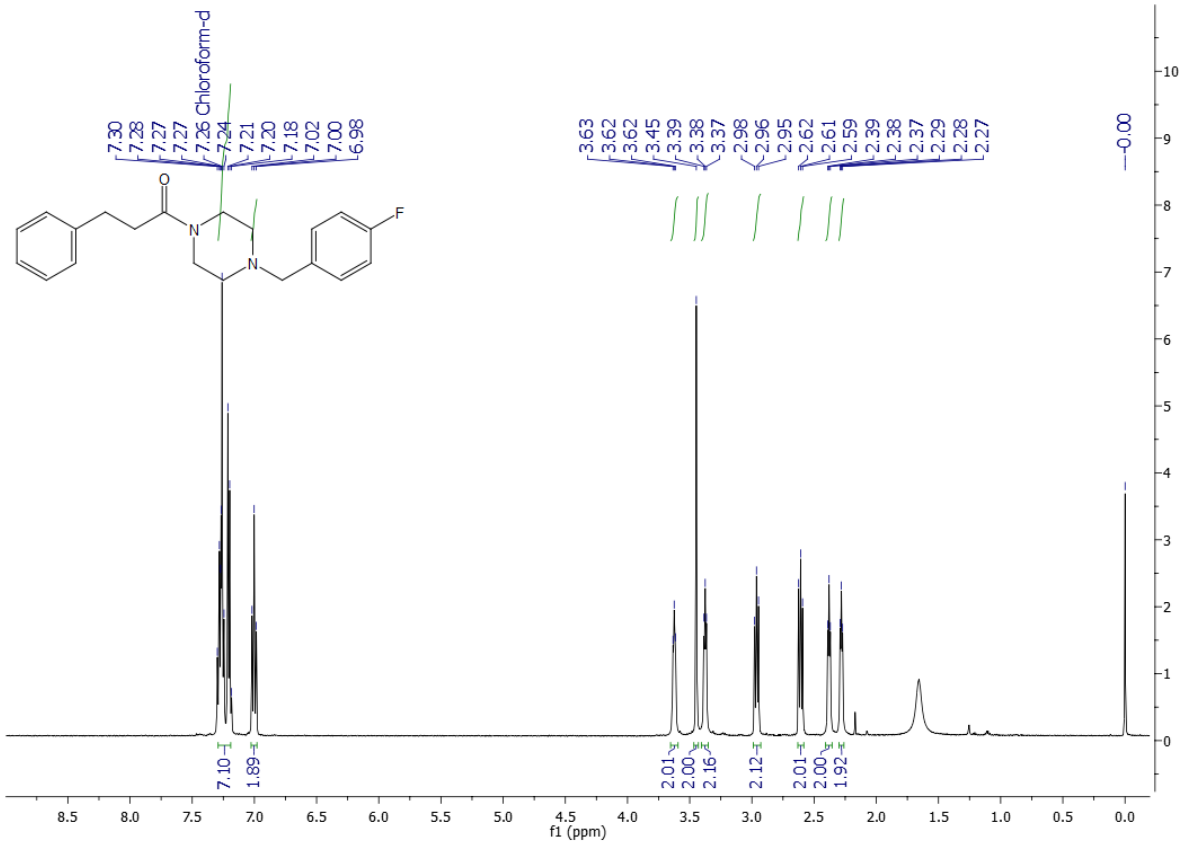

Figure S4: ^{1}H-NMR ($CDCl_3$) spectrum of 1-[4-(4-Fluorobenzyl)piperazin-1-yl]-3-phenylpropan-1-one (**8**)

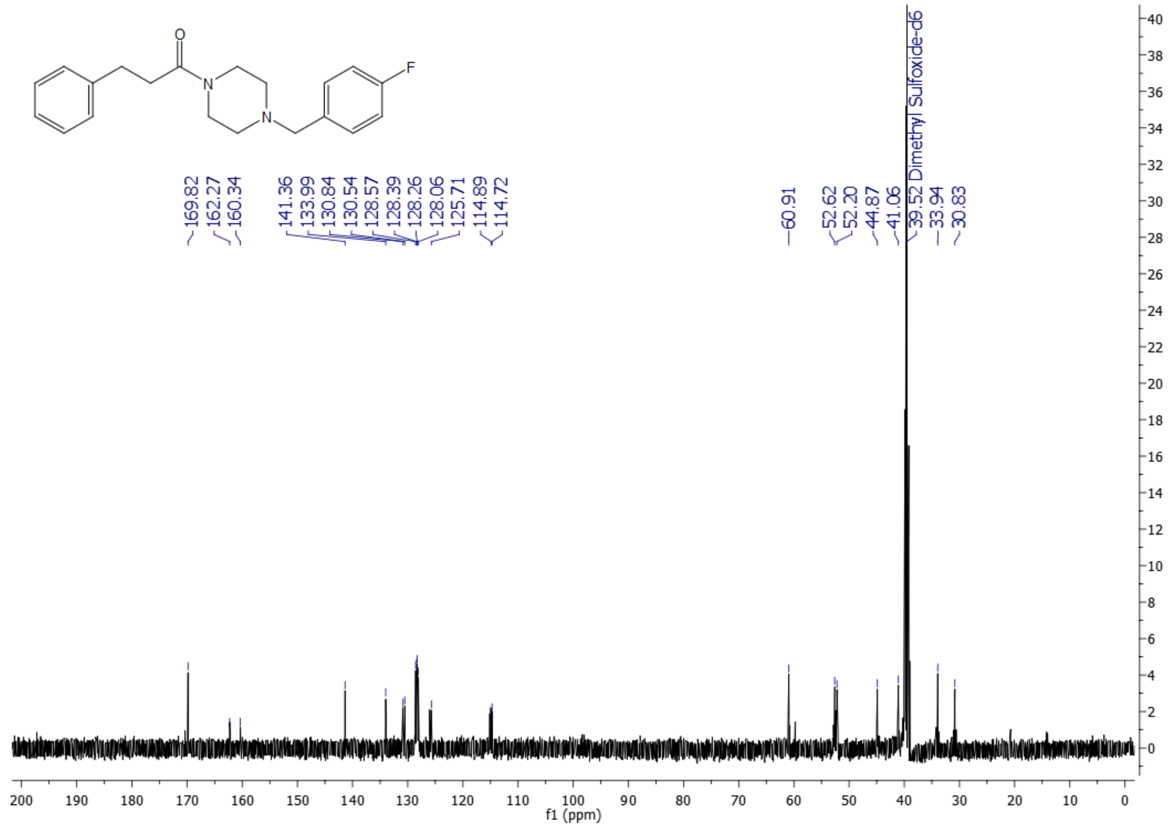

Figure S5: ^{13}C-NMR (DMSO-d6) spectrum of 1-[4-(4-Fluorobenzyl)piperazin-1-yl]-3-phenylpropan-1-one (**8**)

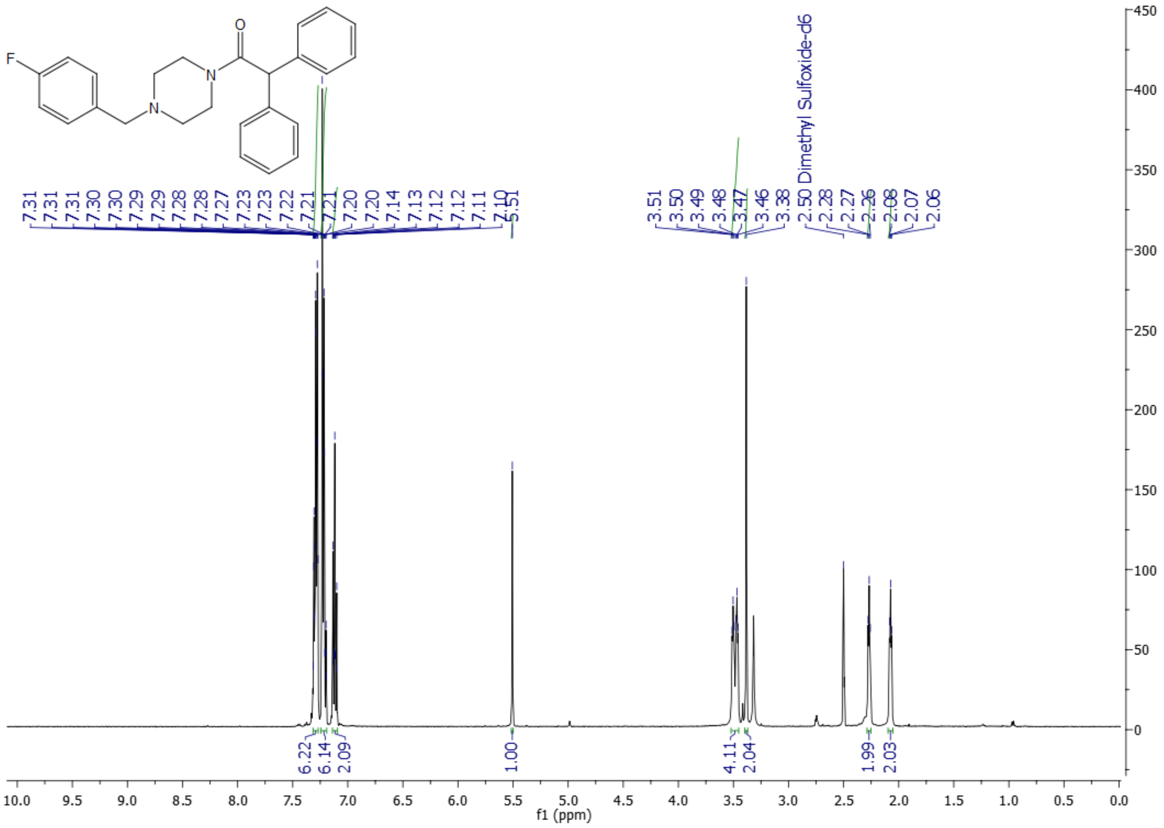

Figure S6: ^{1}H-NMR (DMSO-d6) spectrum of 1-[4-(4-Fluorobenzyl)piperazin-1-yl]-2,2-diphenylethan-1-one (**9**)

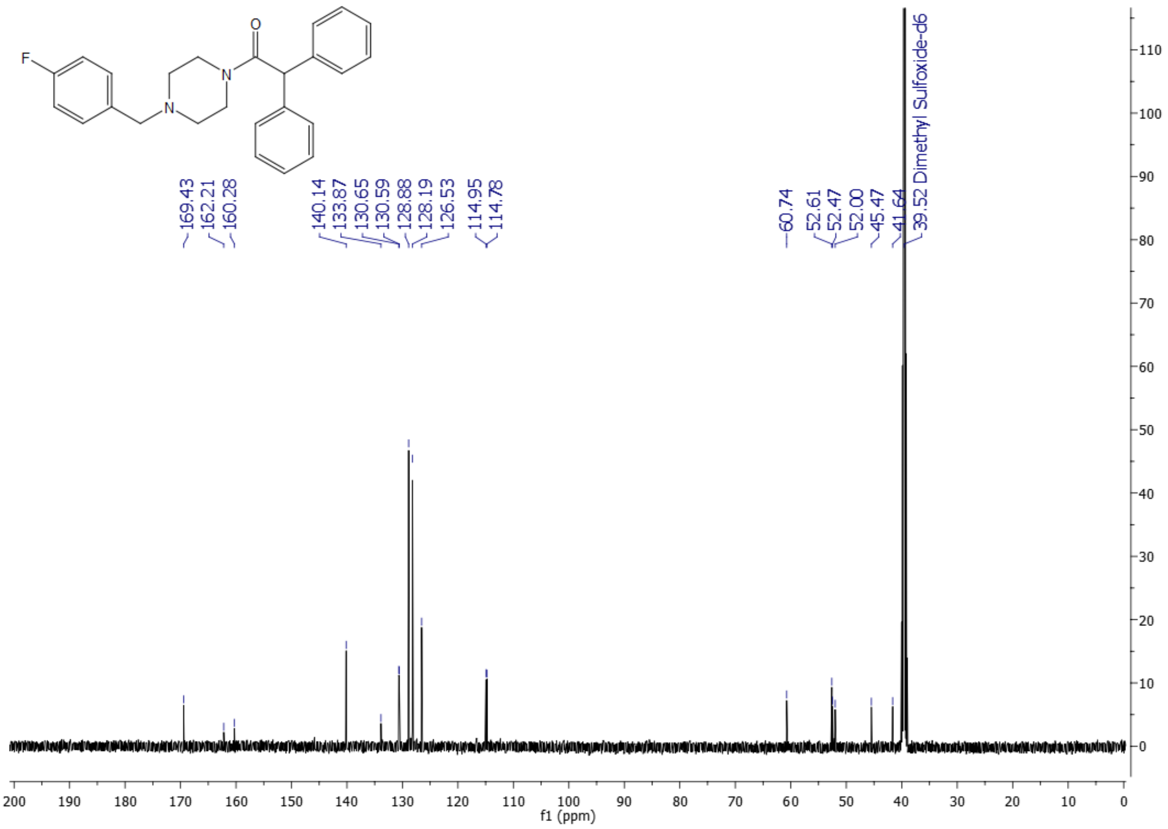

Figure S7: ^{13}C-NMR (DMSO-d6) spectrum of 1-[4-(4-Fluorobenzyl)piperazin-1-yl]-2,2-diphenylethan-1-one (**9**)

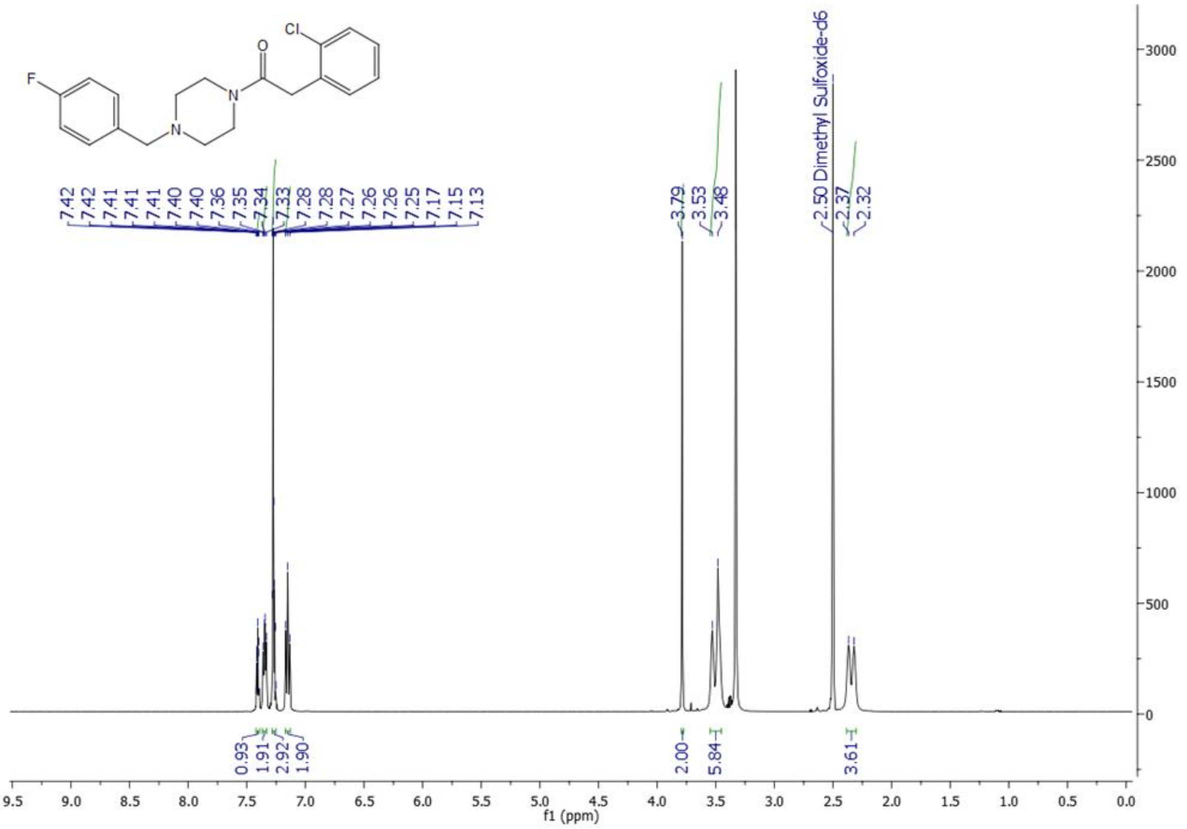

Figure S8: ^{1}H-NMR (DMSO-d6) spectrum of 1-[4-(4-Fluorobenzyl)piperazin-1-yl]-2-(2-chlorophenyl)ethan-1-one (**10**)

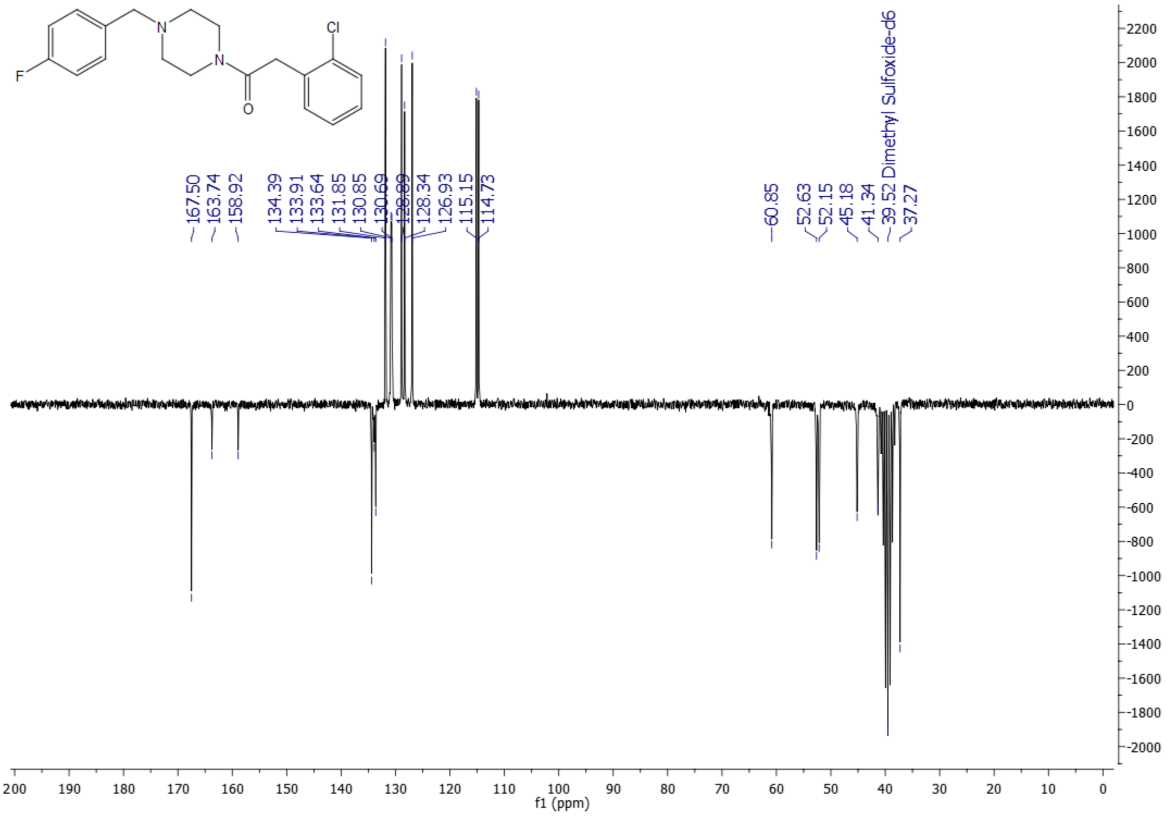

Figure S9: ^{13}C-NMR (DMSO-d6) spectrum of 1-[4-(4-Fluorobenzyl)piperazin-1-yl]-2-(2-chlorophenyl)ethan-1-one (**10**)

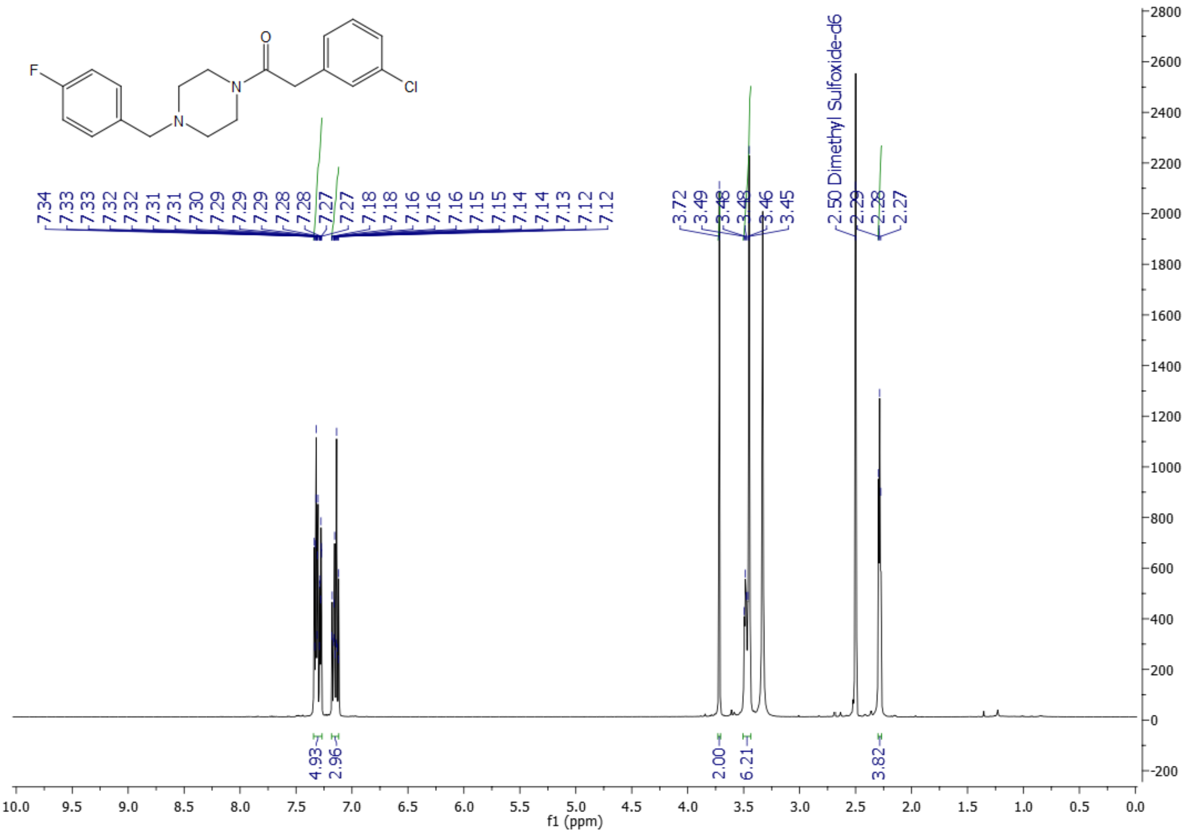

Figure S10: ^{1}H-NMR (DMSO-d6) spectrum of 1-[4-(4-Fluorobenzyl)piperazin-1-yl]-2-(3-chlorophenyl)ethan-1-one (**11**)

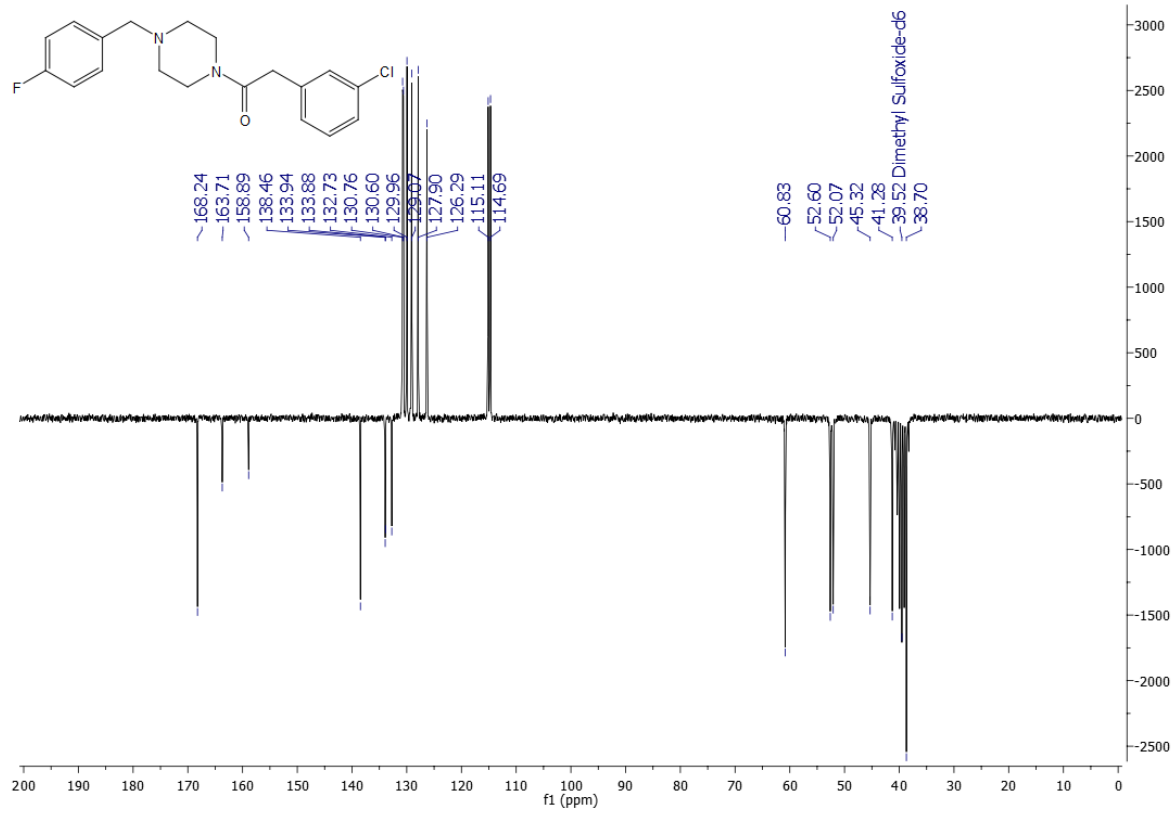

Figure S11: ^{13}C-NMR (DMSO-d6) spectrum of 1-[4-(4-Fluorobenzyl)piperazin-1-yl]-2-(3-chlorophenyl)ethan-1-one (**11**)

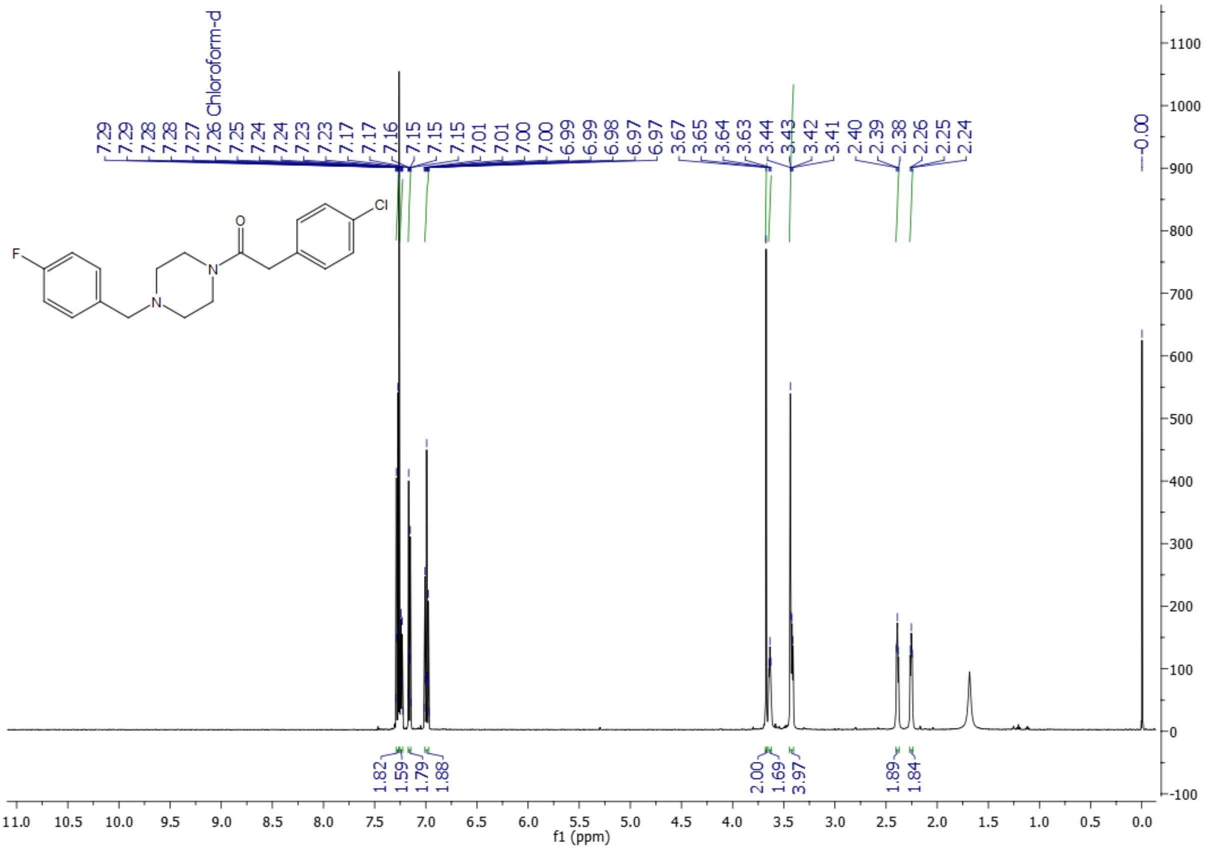

Figure S12: ^{1}H-NMR ($CDCl_3$) spectrum of 1-[4-(4-Fluorobenzyl)piperazin-1-yl]-2-(4-chlorophenyl)ethan-1-one (**12**)

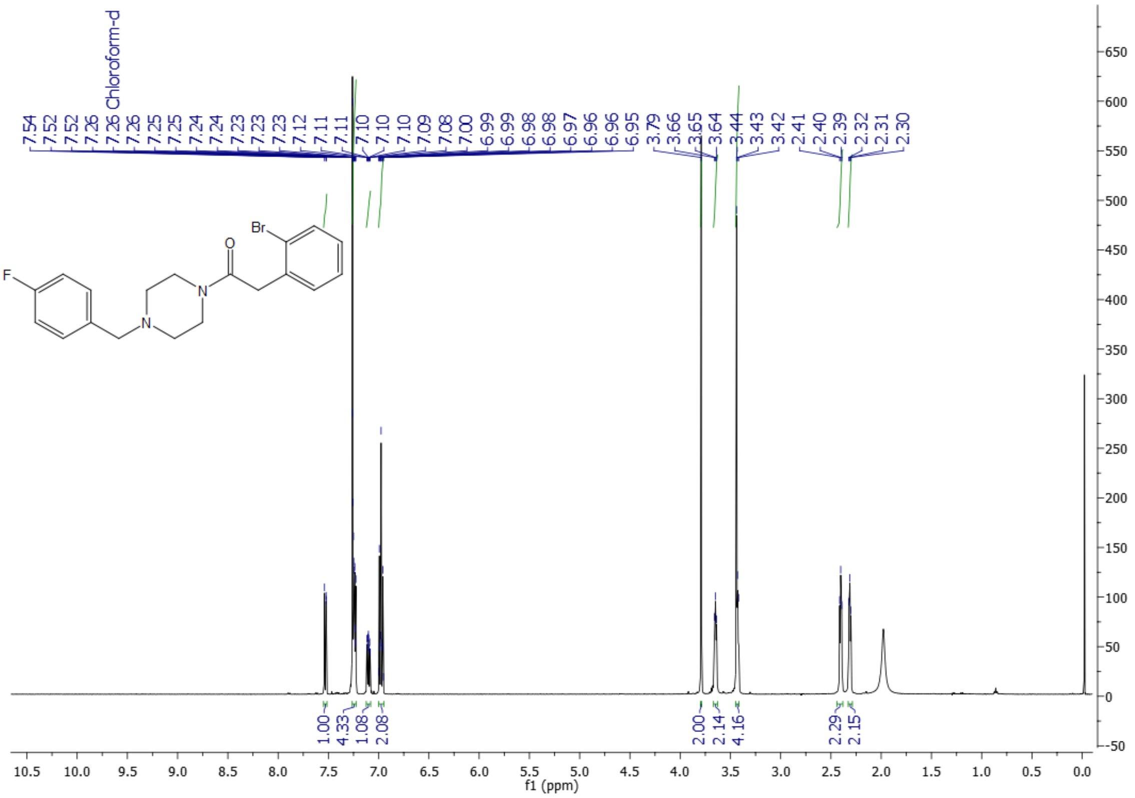

Figure S13: ^{1}H-NMR ($CDCl_3$) spectrum of 1-[4-(4-Fluorobenzyl)piperazin-1-yl]-2-(2-bromophenyl)ethan-1-one (**13**)

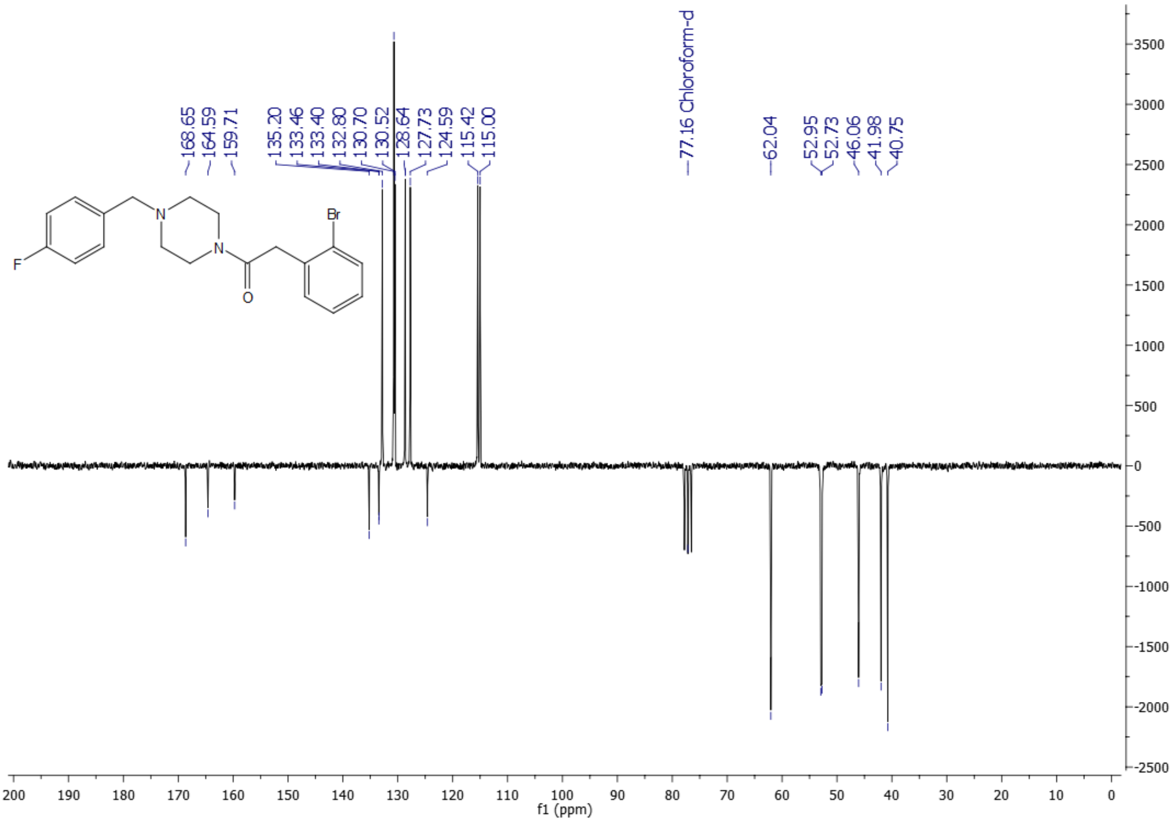

Figure S14: ^{13}C-NMR ($CDCl_3$) spectrum of 1-[4-(4-Fluorobenzyl)piperazin-1-yl]-2-(2-bromophenyl)ethan-1-one (**13**)

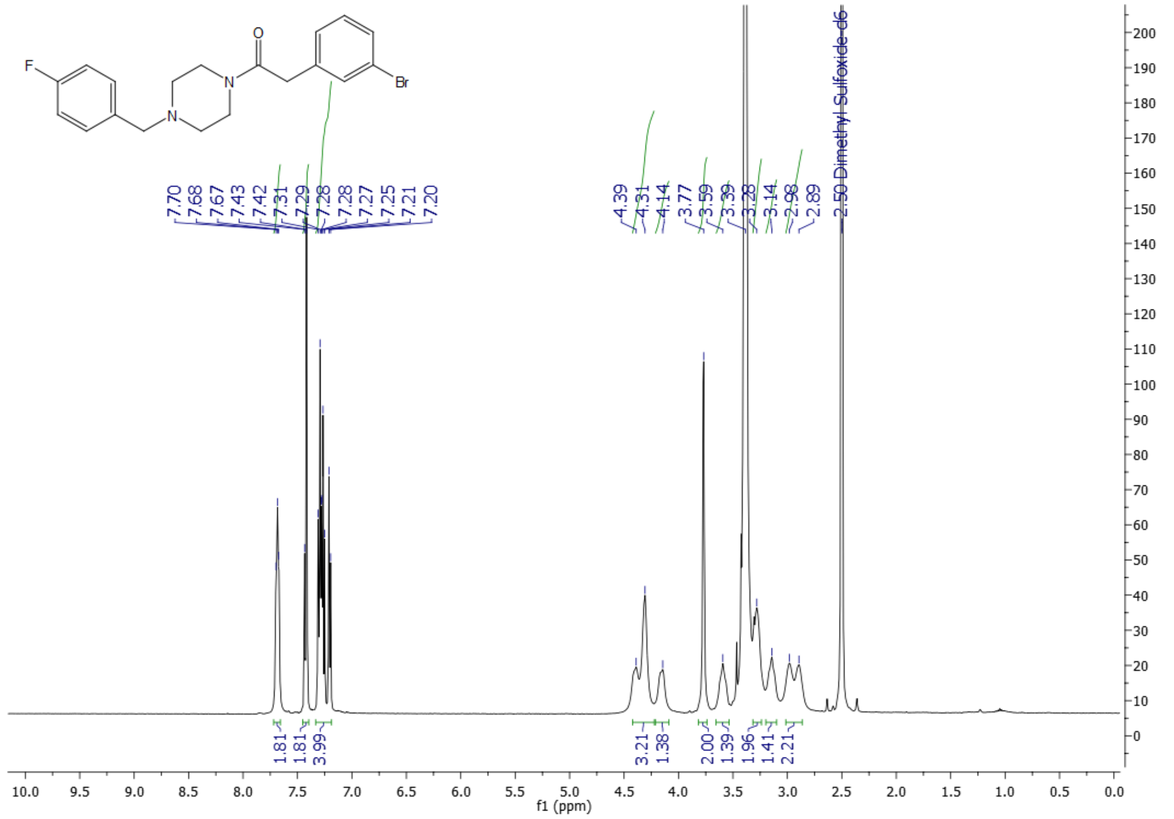

Figure S15: ^{1}H-NMR (DMSO-d6) spectrum of 1-[4-(4-Fluorobenzyl)piperazin-1-yl]-2-(3-bromophenyl)ethan-1-one (**14**)

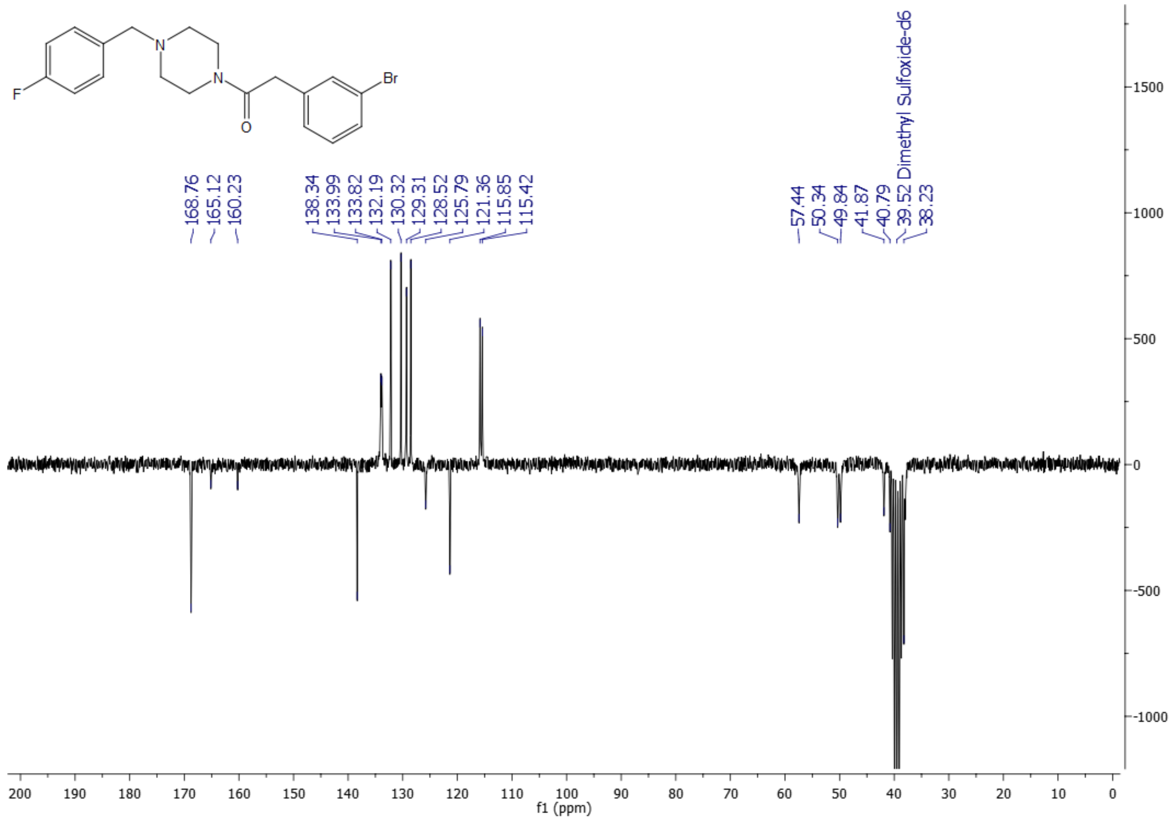

Figure S16: ^{13}C-NMR (DMSO-d6) spectrum of 1-[4-(4-Fluorobenzyl)piperazin-1-yl]-2-(3-bromophenyl)ethan-1-one (**14**)

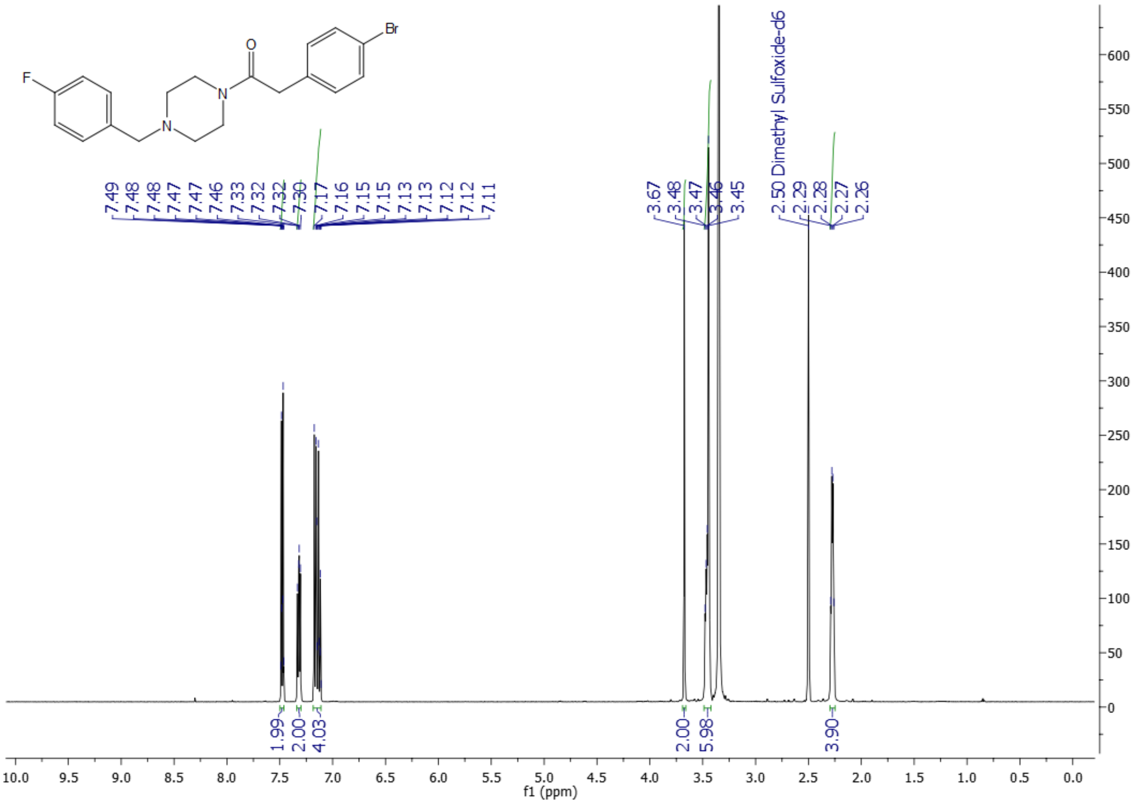

Figure S17: ^{1}H-NMR (DMSO-d6) spectrum of 1-[4-(4-Fluorobenzyl)piperazin-1-yl]-2-(4-bromophenyl)ethan-1-one (**15**)

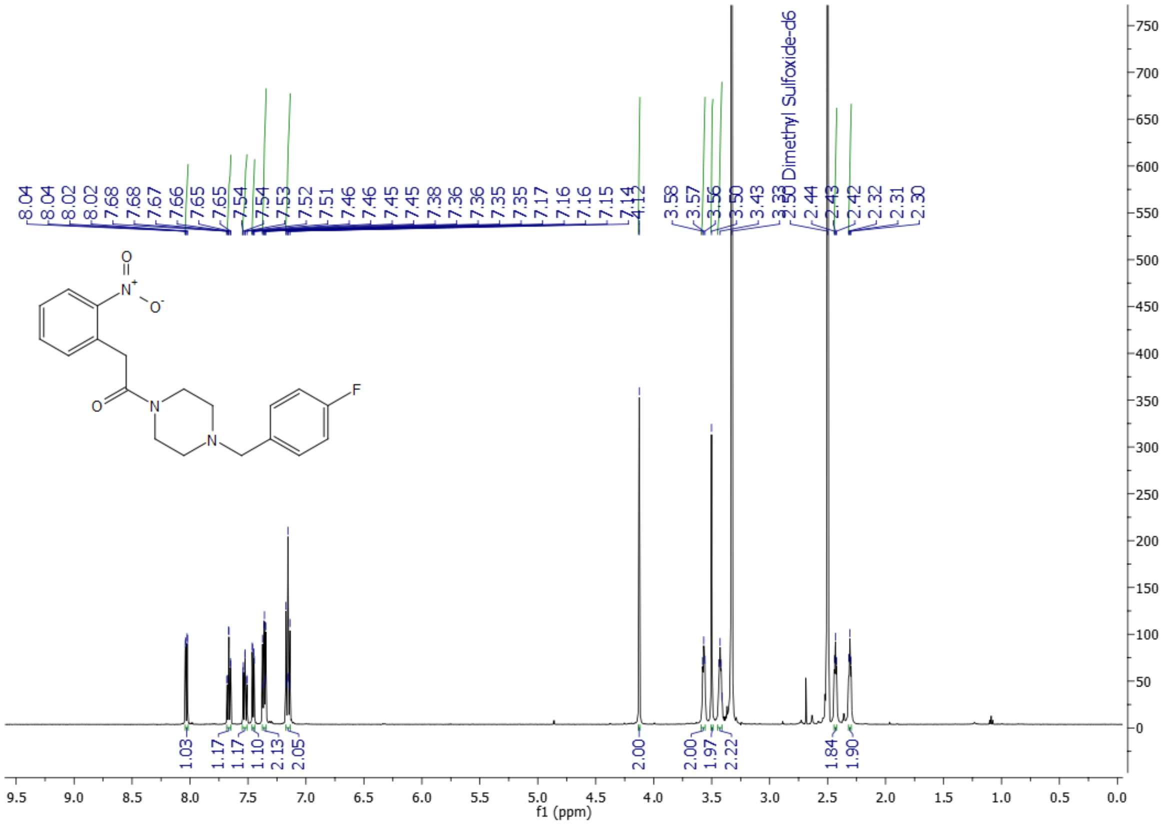

Figure S18: ^{1}H-NMR (DMSO-d6) spectrum of 1-[4-(4-Fluorobenzyl)piperazin-1-yl]-2-(2-nitrophenyl)ethan-1-one (**16**)

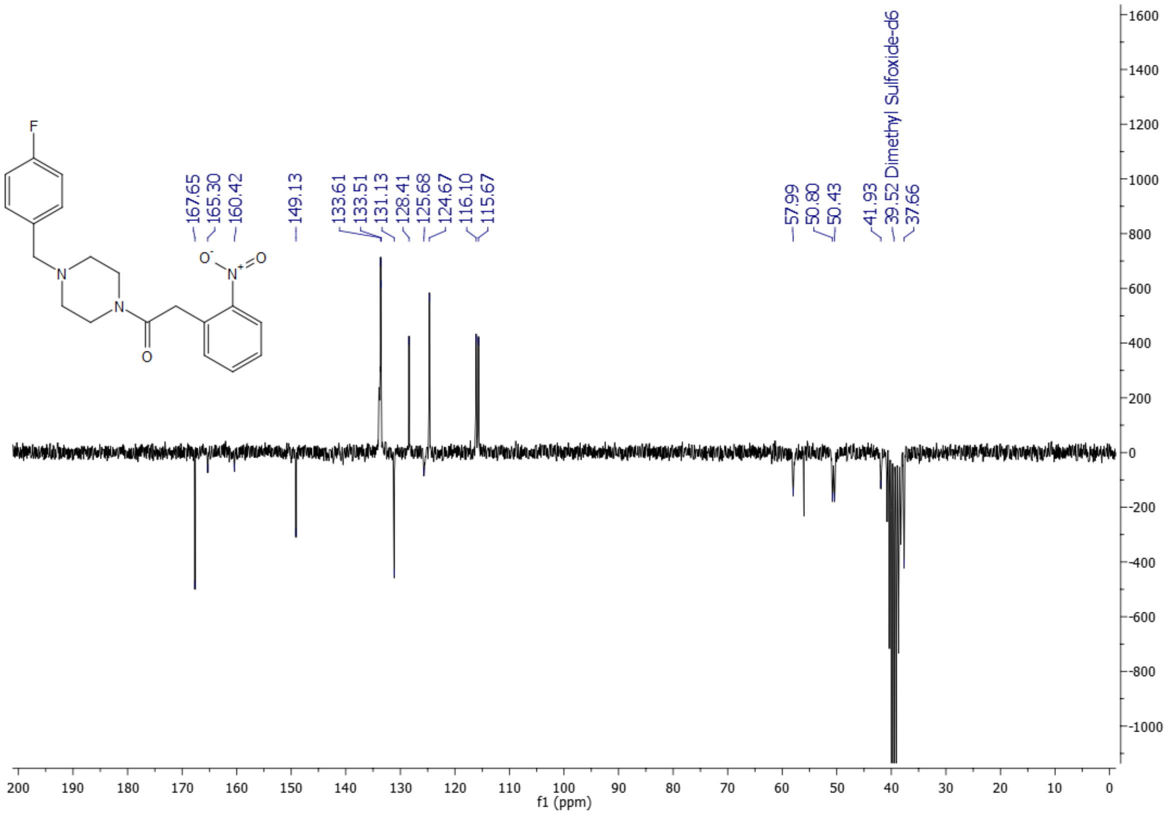

Figure S19: ^{13}C-NMR (DMSO-d6) spectrum of 1-[4-(4-Fluorobenzyl)piperazin-1-yl]-2-(2-nitrophenyl)ethan-1-one (**16**)

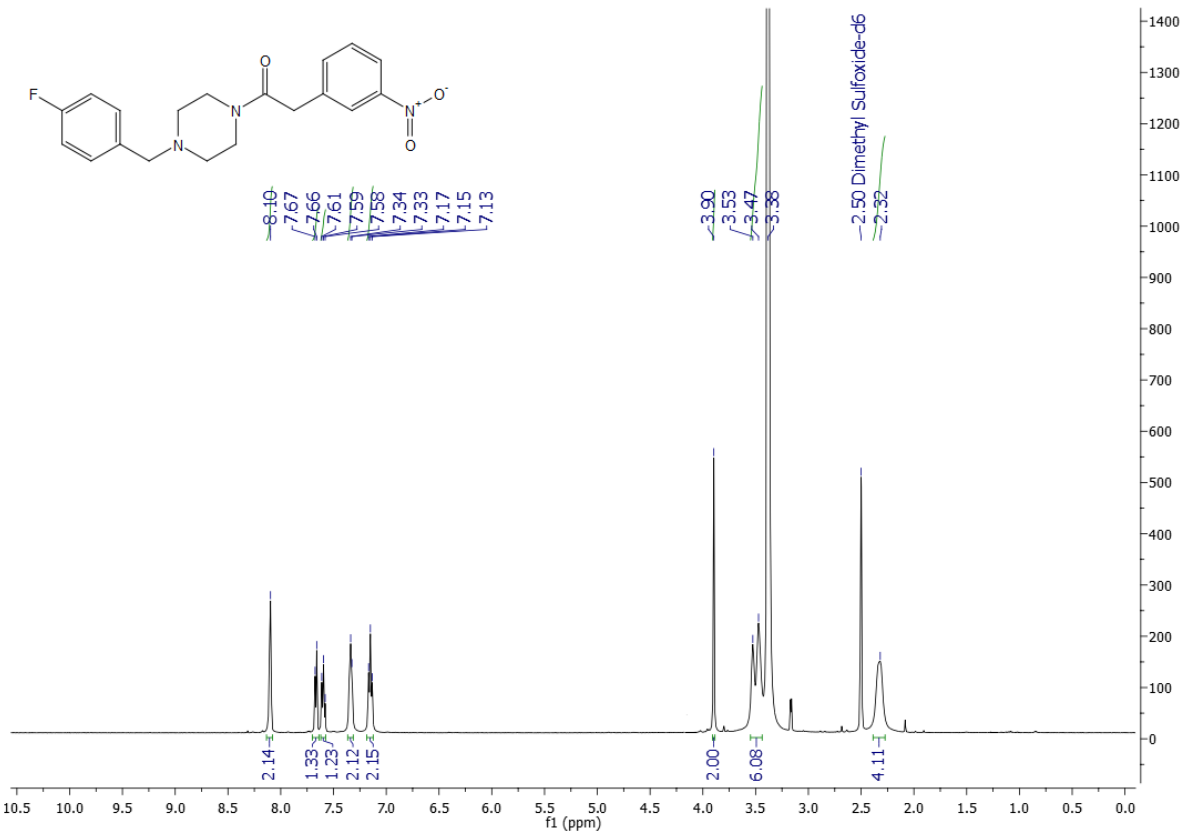

Figure S20: ^{1}H-NMR (DMSO-d6) spectrum of 1-[4-(4-Fluorobenzyl)piperazin-1-yl]-2-(3-nitrophenyl)ethan-1-one (**17**)

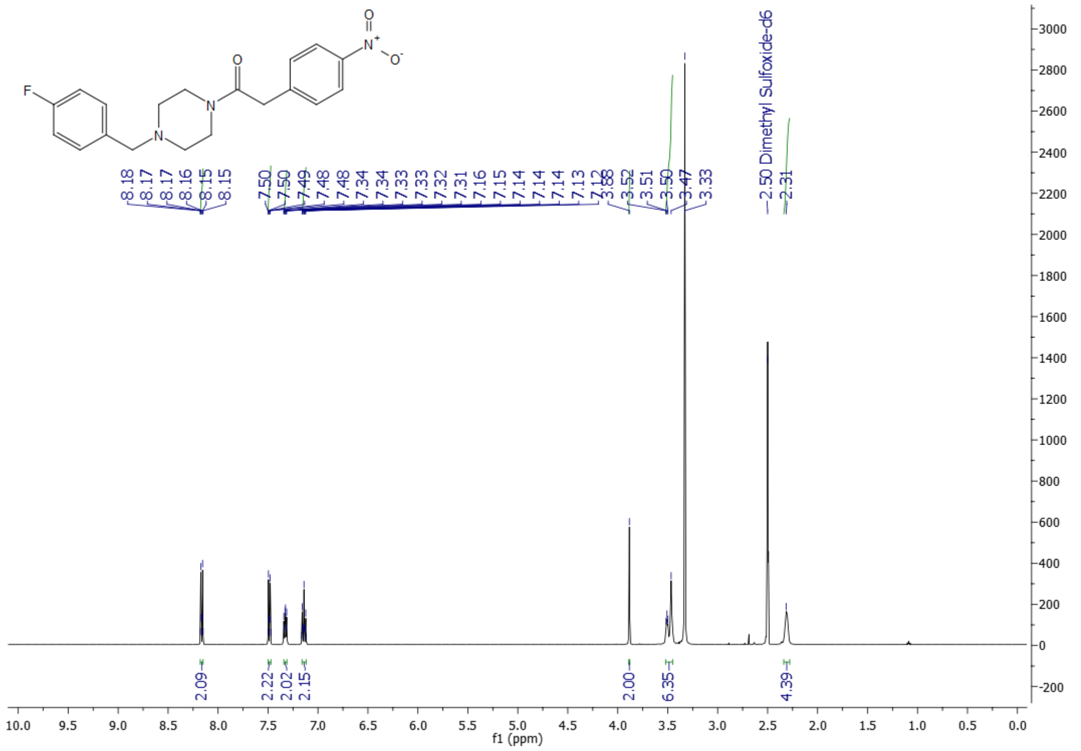

Figure S21: ^{1}H-NMR (DMSO-d6) spectrum of 1-[4-(4-Fluorobenzyl)piperazin-1-yl]-2-(4-nitrophenyl)ethan-1-one (**18**)

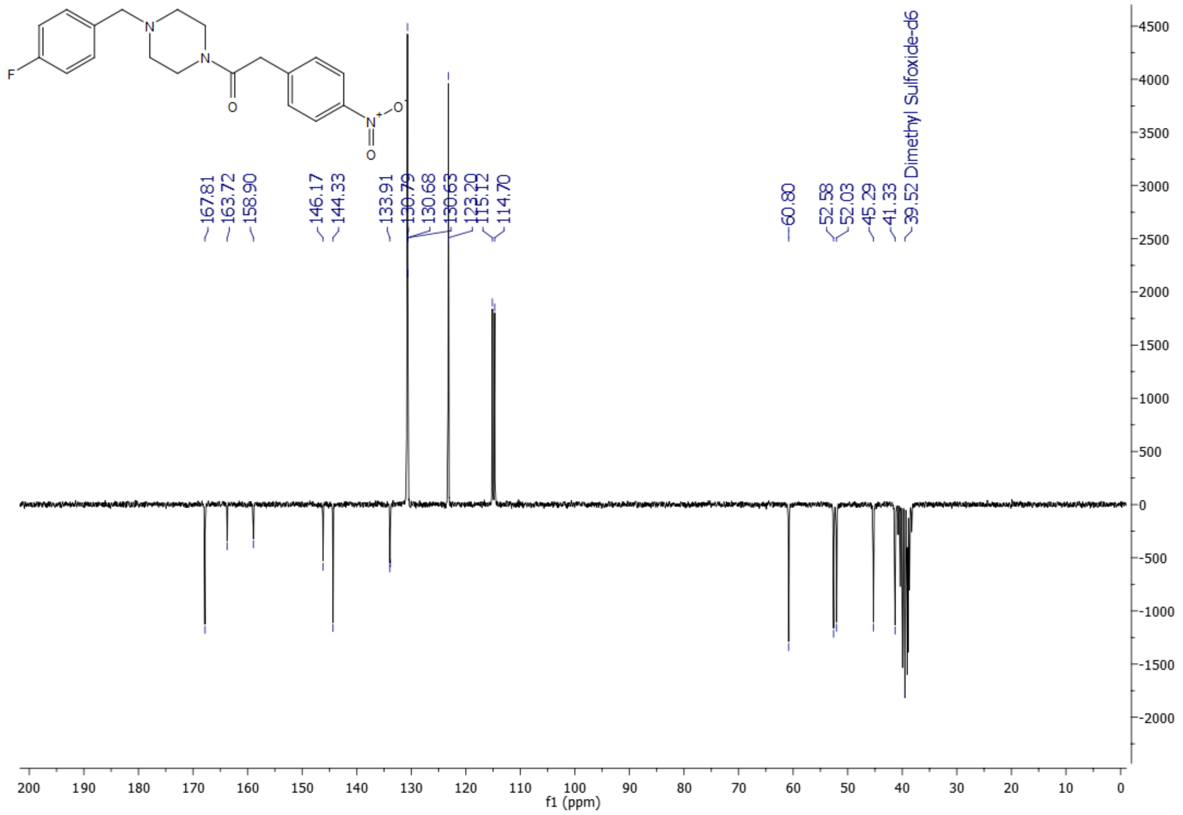

Figure S22: ^{13}C-NMR (DMSO-d6) spectrum of 1-[4-(4-Fluorobenzyl)piperazin-1-yl]-2-(4-nitrophenyl)ethan-1-one (**18**)

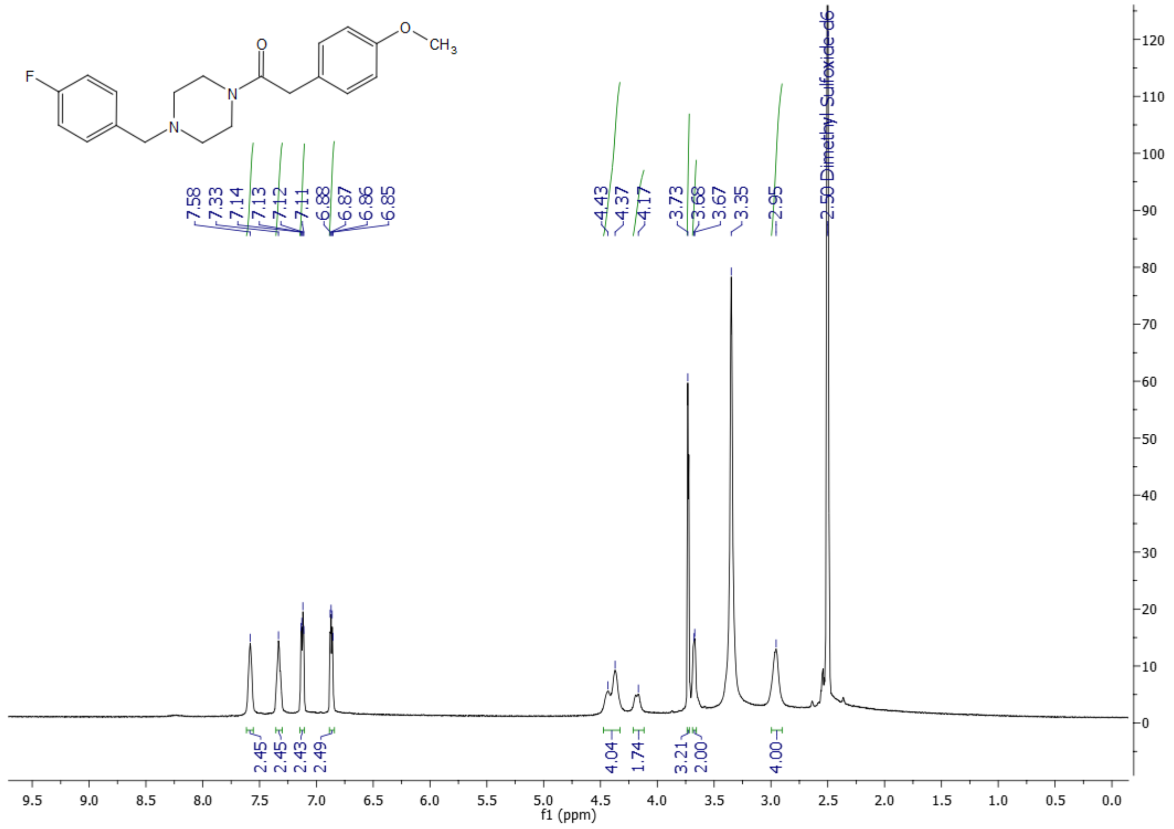

Figure S23: ^{1}H-NMR (DMSO-d6) spectrum of 1-[4-(4-Fluorobenzyl)piperazin-1-yl]-2-(4-methoxyphenyl)ethan-1-one (**19**)

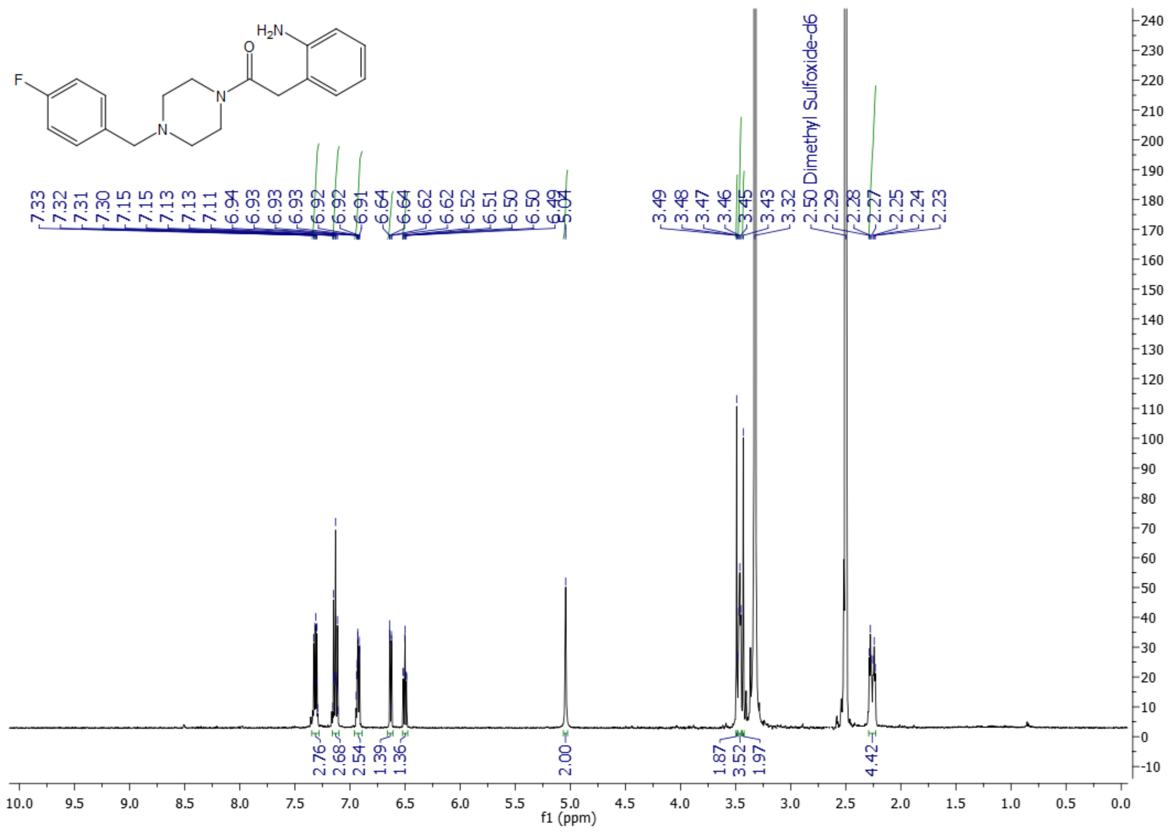

Figure S24: ^{1}H-NMR (DMSO-d6) spectrum of 1-[4-(4-Fluorobenzyl)piperazin-1-yl]-2-(2-aminophenyl)ethan-1-one (**20**)

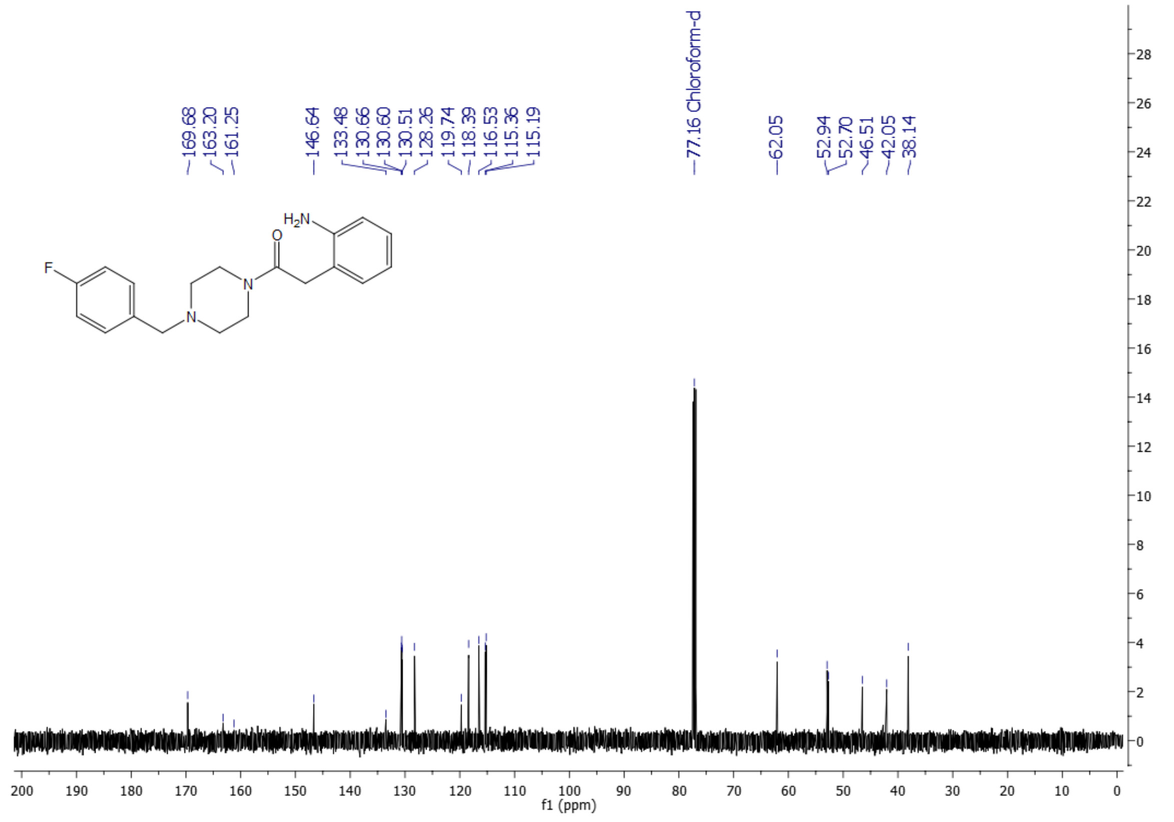

Figure S25: ^{13}C-NMR ($CDCl_3$) spectrum of 1-[4-(4-Fluorobenzyl)piperazin-1-yl]-2-(2-aminophenyl)ethan-1-one (**20**)

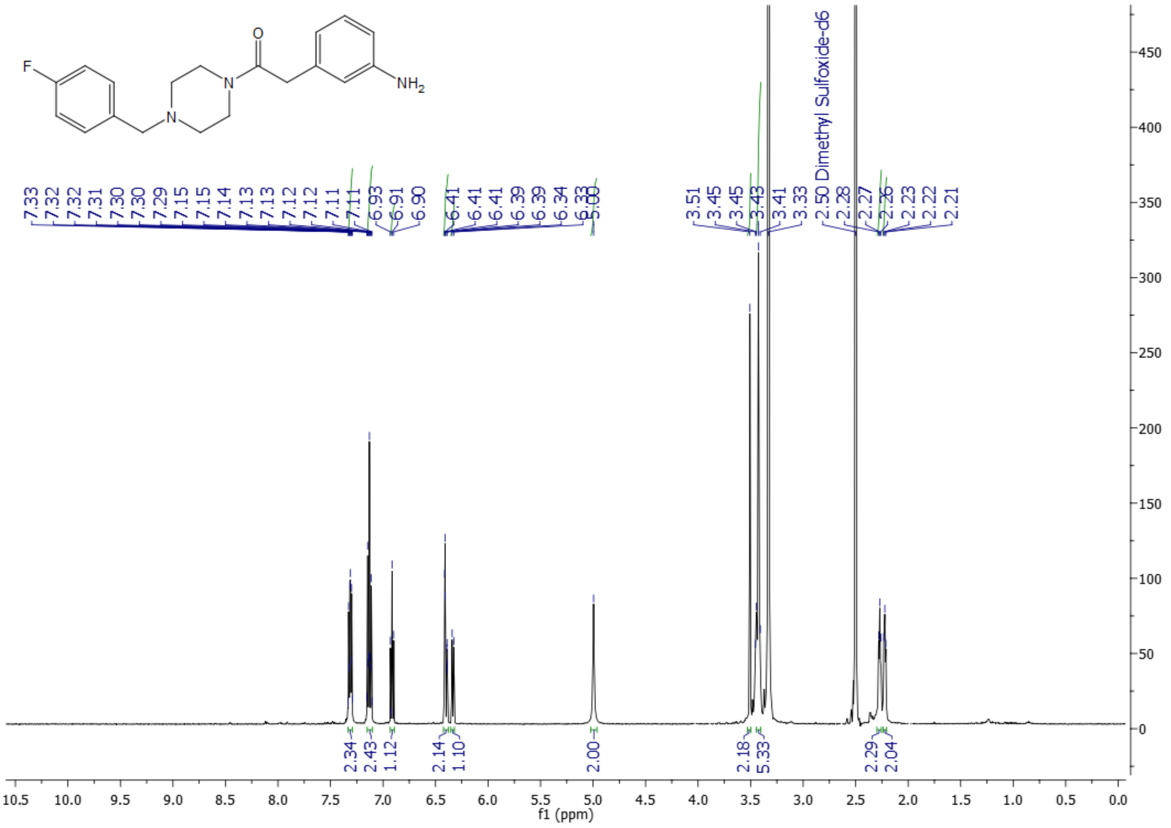

Figure S26: ^{1}H-NMR (DMSO-d6) spectrum of 1-[4-(4-Fluorobenzyl)piperazin-1-yl]-2-(3-aminophenyl)ethan-1-one (**21**)

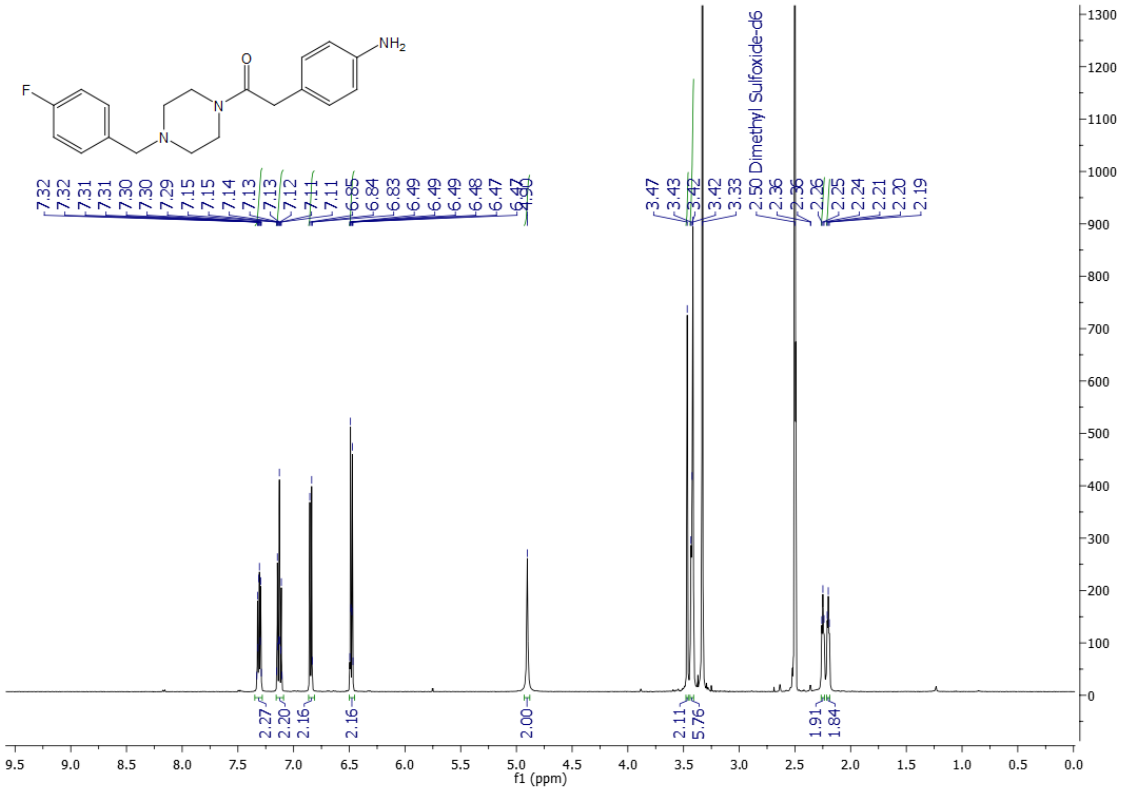

Figure S27: ^{1}H-NMR (DMSO-d6) spectrum of 1-[4-(4-Fluorobenzyl)piperazin-1-yl]-2-(4-aminophenyl)ethan-1-one (**22**)

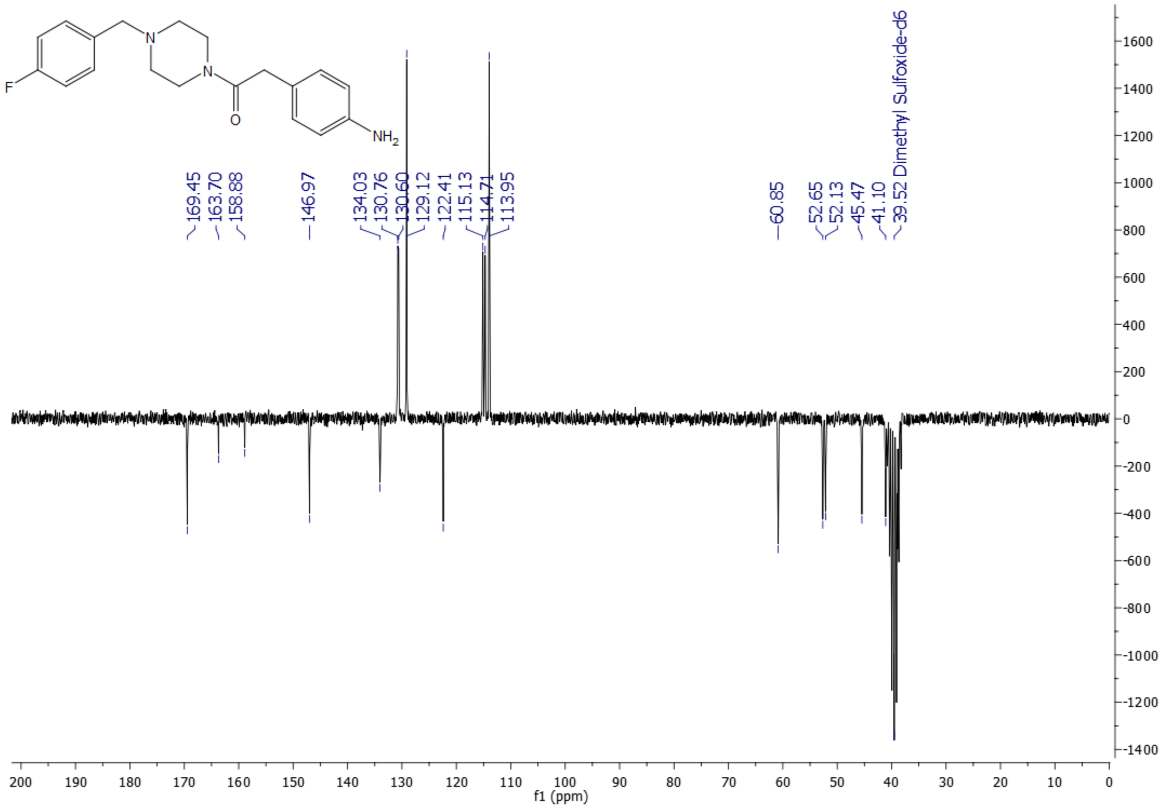

Figure S28: ^{13}C-NMR (DMSO-d6) spectrum of 1-[4-(4-Fluorobenzyl)piperazin-1-yl]-2-(4-aminophenyl)ethan-1-one (**22**)

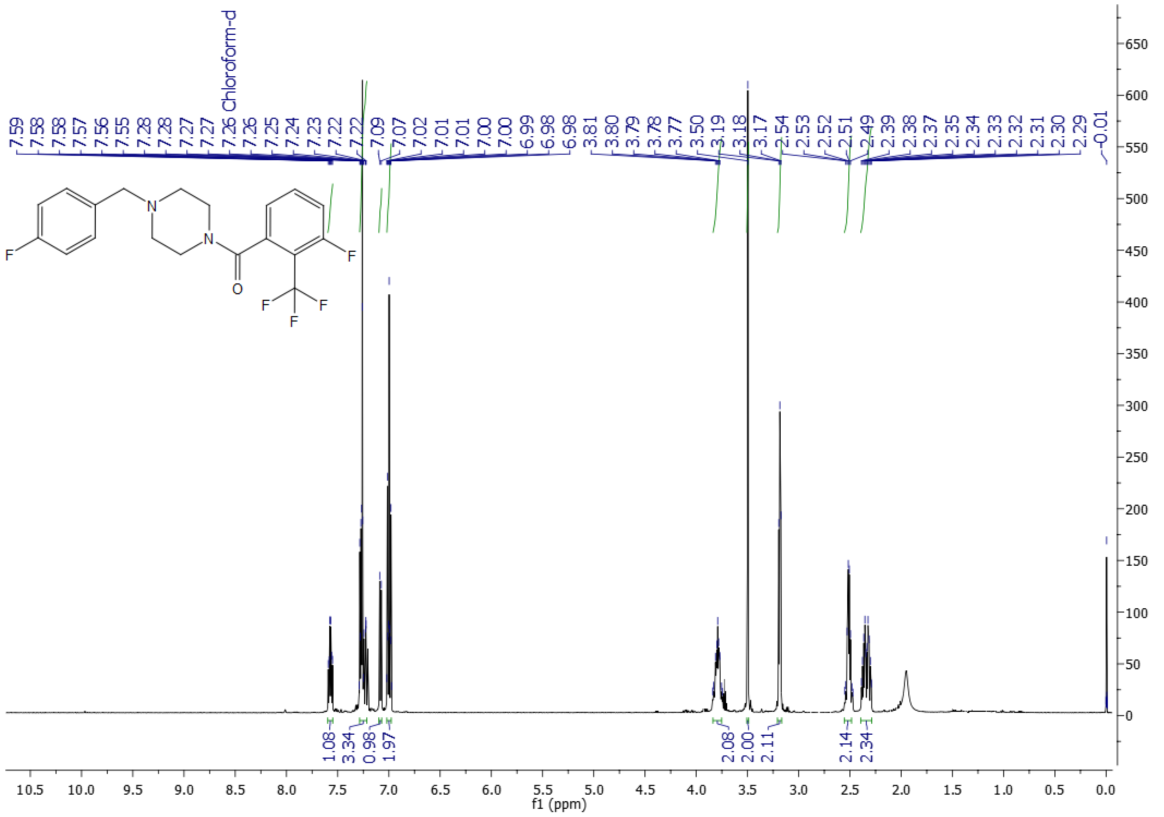

Figure S29: ^{1}H-NMR ($CDCl_3$) spectrum of [4-(4-Fluorobenzyl)piperazin-1-yl]-[3-fluoro-2-(trifluoromethyl)phenyl]methanone (**23**)

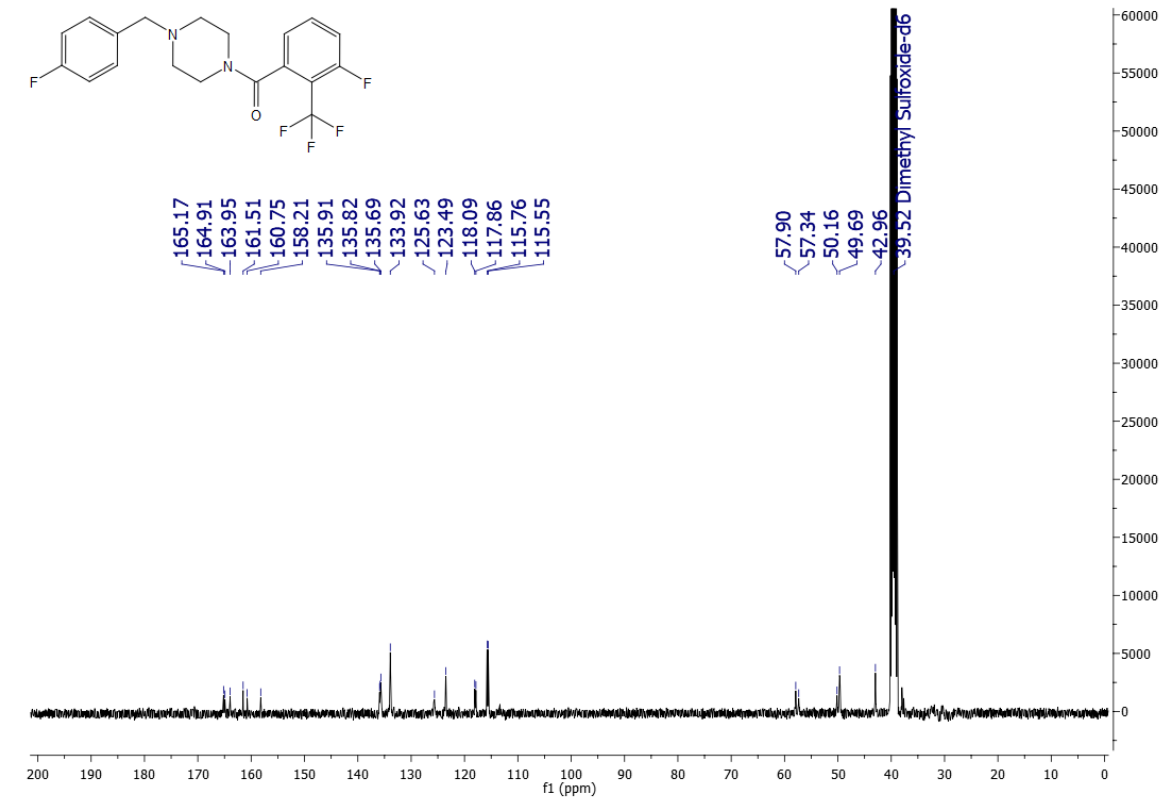

Figure S30: ^{13}C-NMR (DMSO-d6) spectrum of [4-(4-Fluorobenzyl)piperazin-1-yl]-[3-fluoro-2-(trifluoromethyl)phenyl]methanone (**23**)

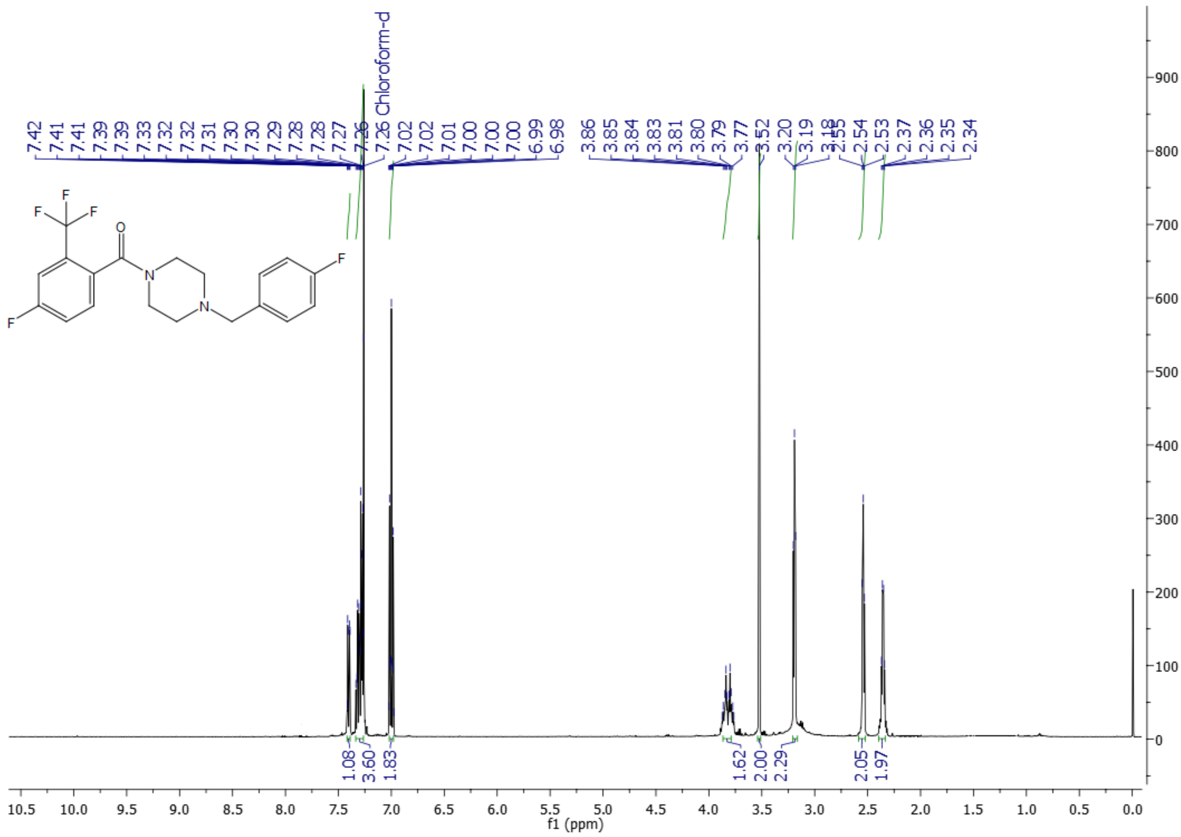

Figure S31: ^{1}H-NMR ($CDCl_3$) spectrum of 1-[4-(4-Fluorobenzyl)piperazin-1-yl]-[4-fluoro-2-(trifluoromethyl)phenyl]methanone (**24**)

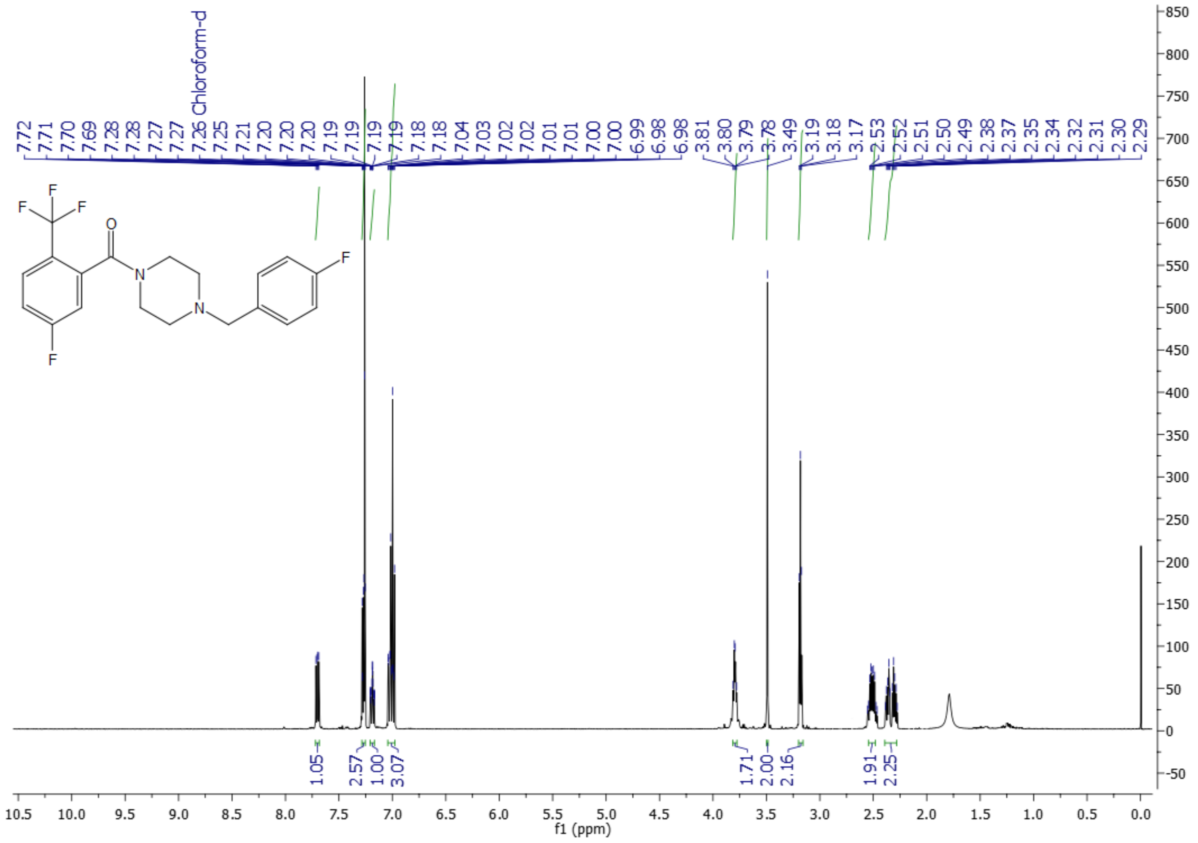

Figure S32: ^{1}H-NMR ($CDCl_3$) spectrum of 1-[4-(4-Fluorobenzyl)piperazin-1-yl]-[5-fluoro-2-(trifluoromethyl)phenyl]methanone (**25**)

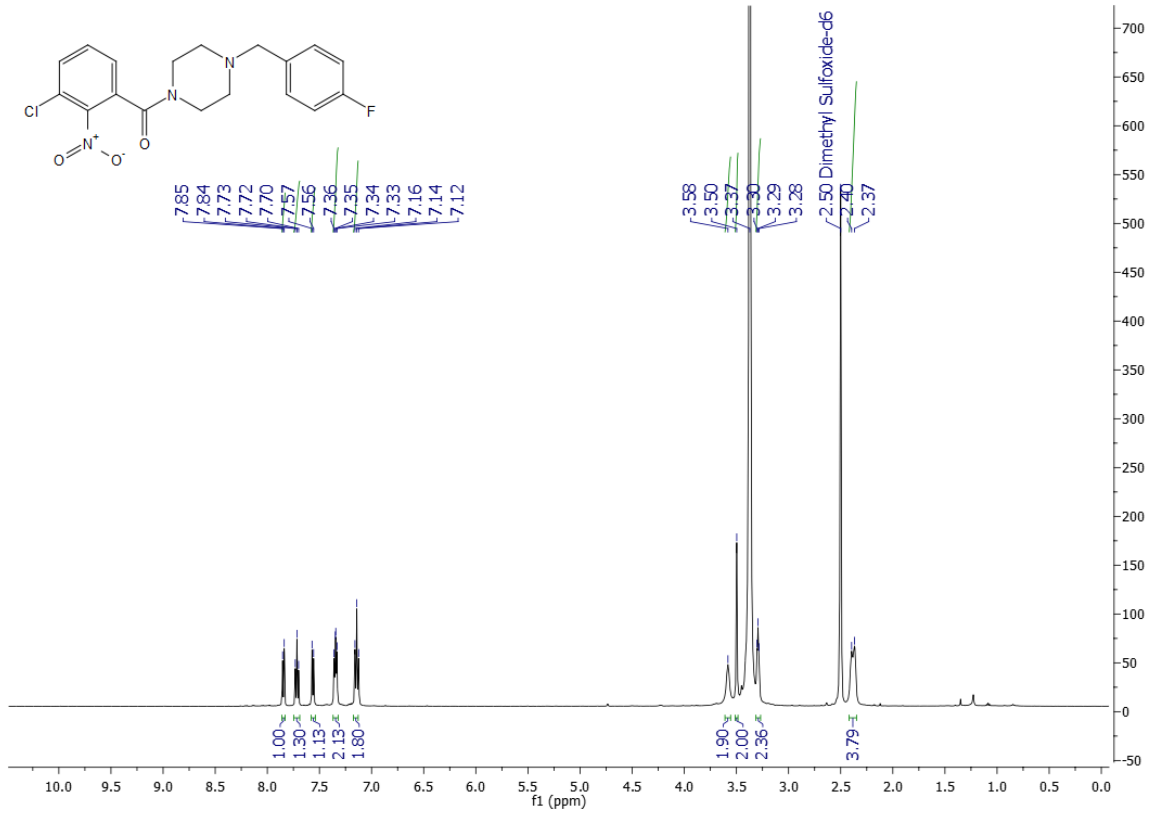

Figure S33: ^{1}H-NMR (DMSO-d6) spectrum of 1-[4-(4-Fluorobenzyl)piperazin-1-yl]-(3-chloro-2-nitro-phenyl)methanone (**26**)

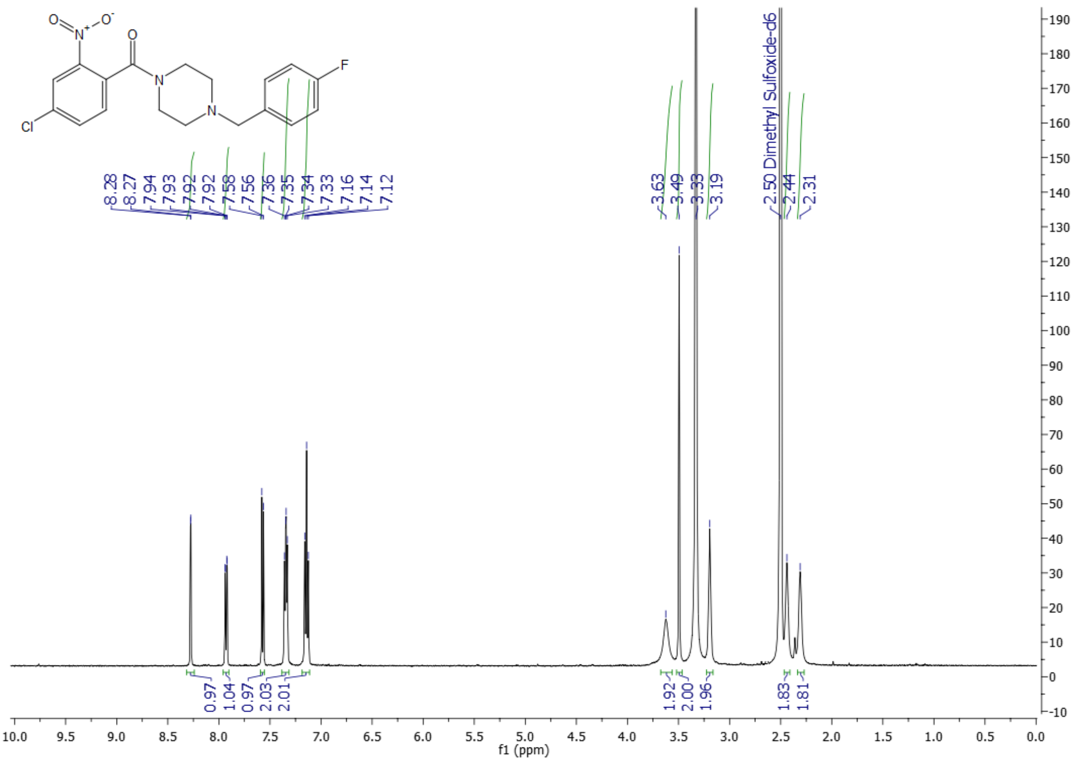

Figure S34: ^{1}H-NMR (DMSO-d6) spectrum of [4-(4-Fluorobenzyl)piperazin-1-yl]-(4-chloro-2-nitro-phenyl)methanone (**27**)

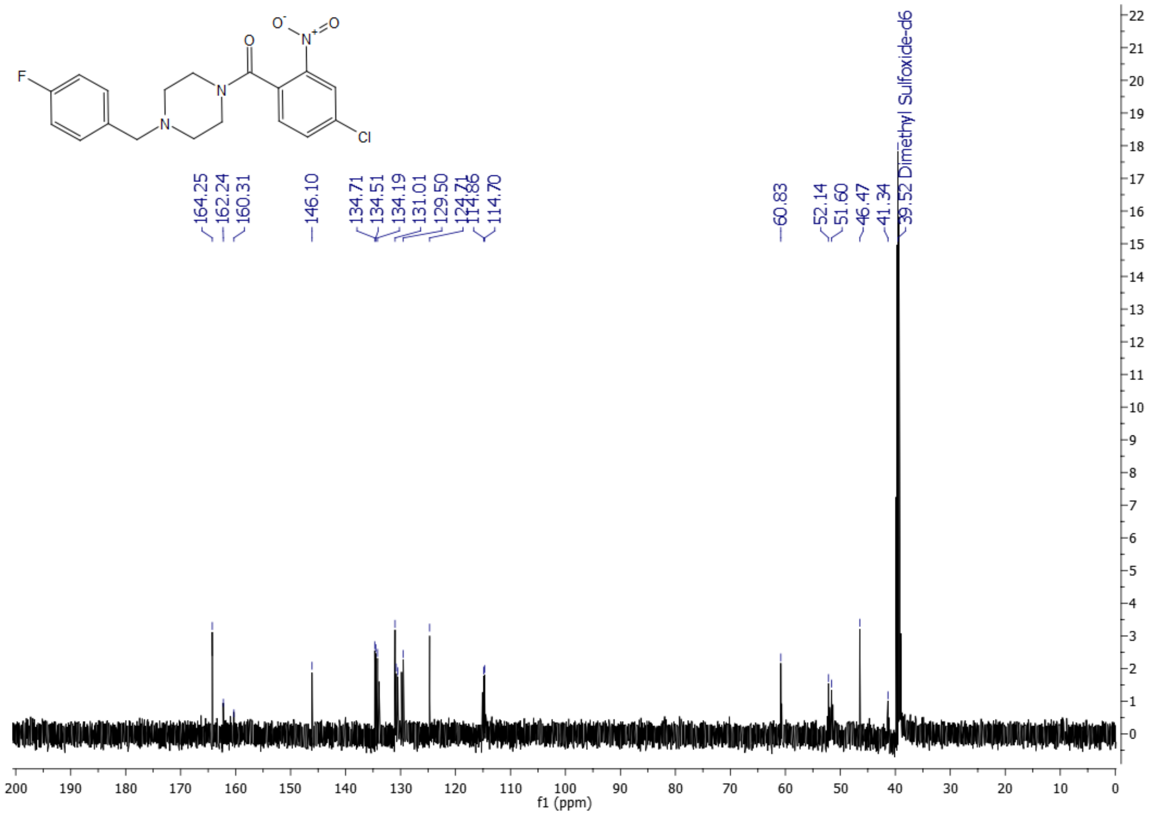

Figure S35: ^{13}C-NMR (DMSO-d6) spectrum of [4-(4-Fluorobenzyl)piperazin-1-yl]-(4-chloro-2-nitro-phenyl)methanone (**27**)

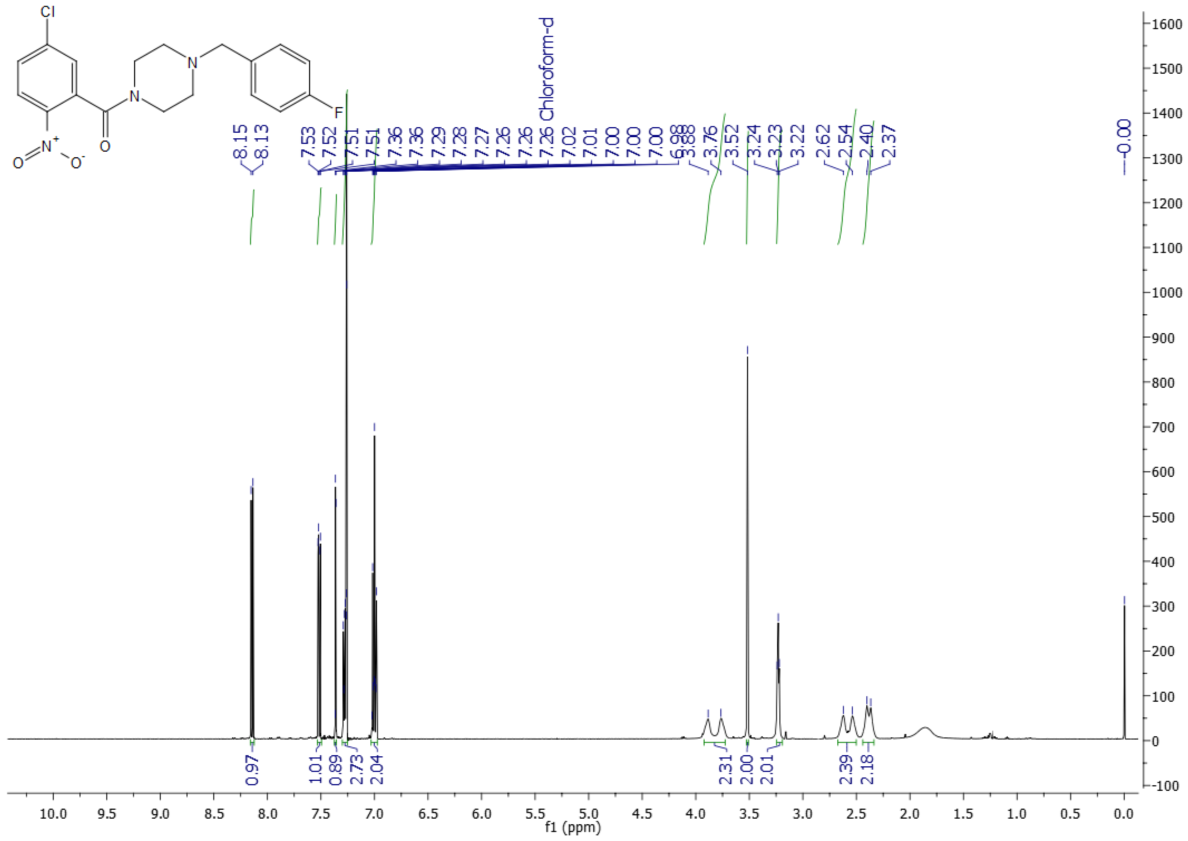

Figure S36: ¹H-NMR ($CDCl_3$) spectrum of 1-[4-(4-Fluorobenzyl)piperazin-1-yl]-(5-chloro-2-nitrophenyl)methanone (**28**)

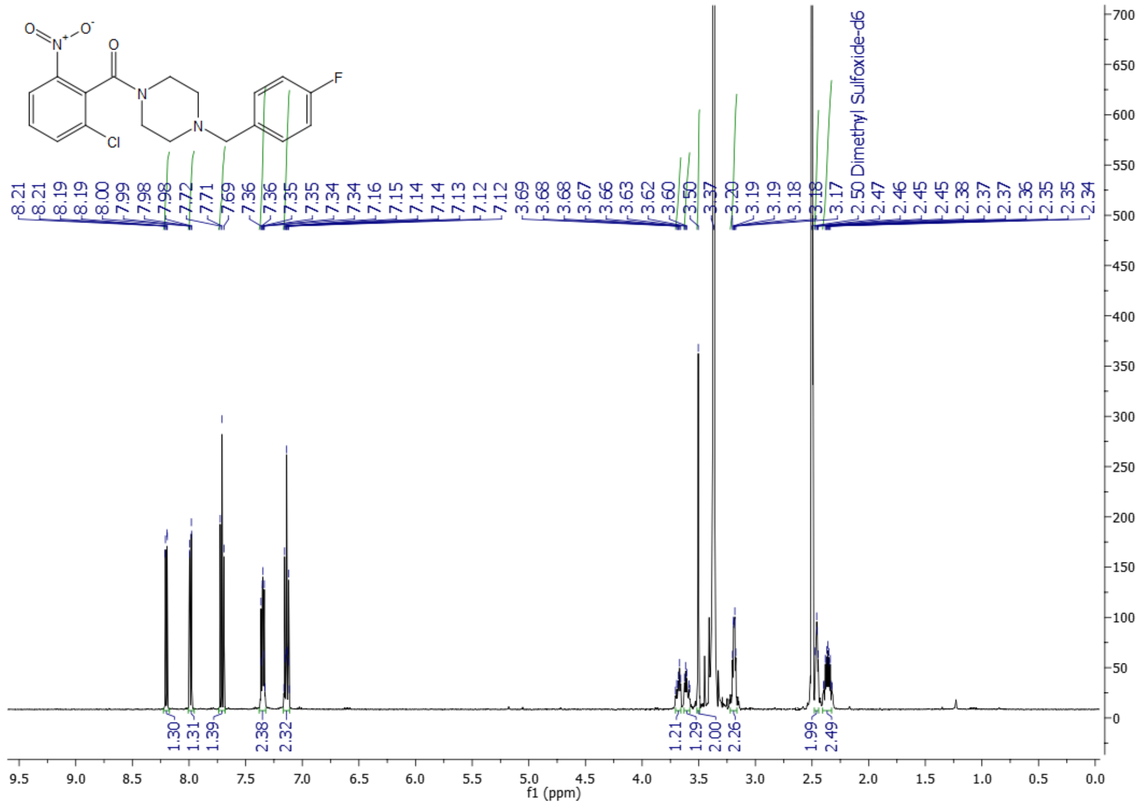

Figure S37: ¹H-NMR (DMSO-d6) spectrum of [4-(4-Fluorobenzyl)piperazin-1-yl]-(2-chloro-6-nitrophenyl)methanone (**29**)

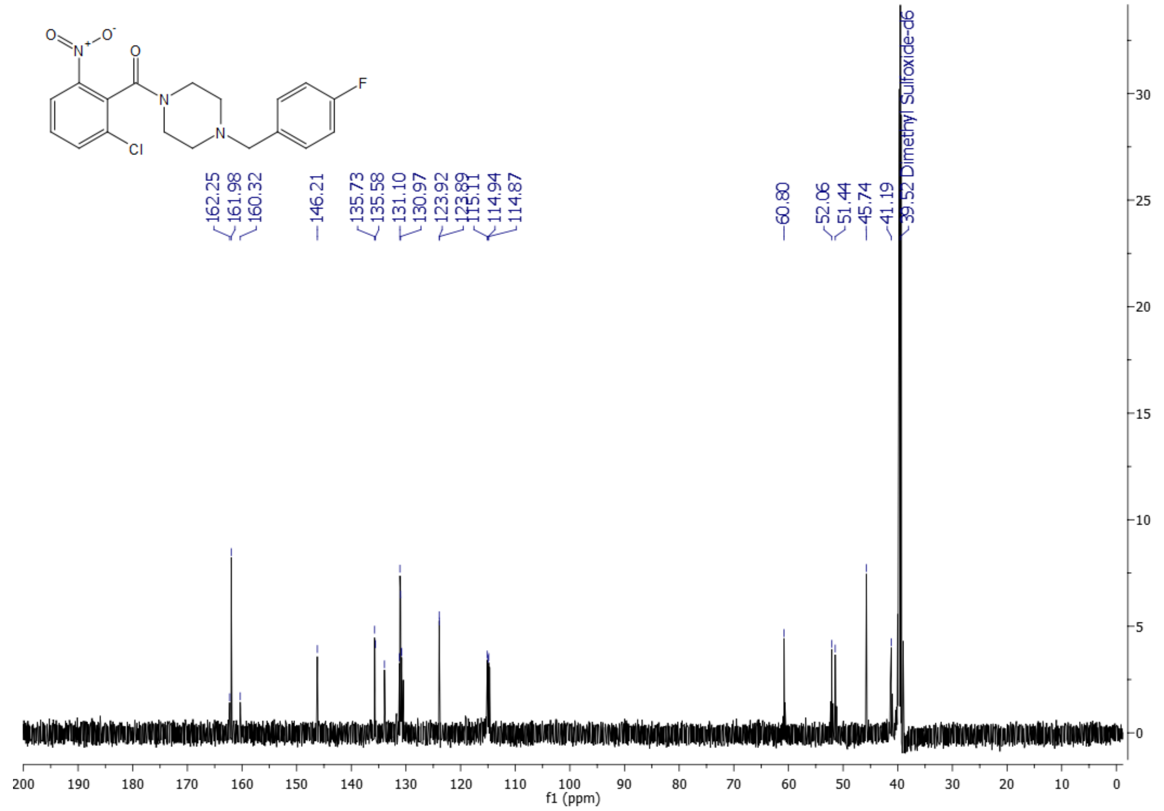

Figure S38: ^{13}C-NMR (DMSO-d6) spectrum of [4-(4-Fluorobenzyl)piperazin-1-yl]-(2-chloro-6-nitro-phenyl)methanone (**29**)

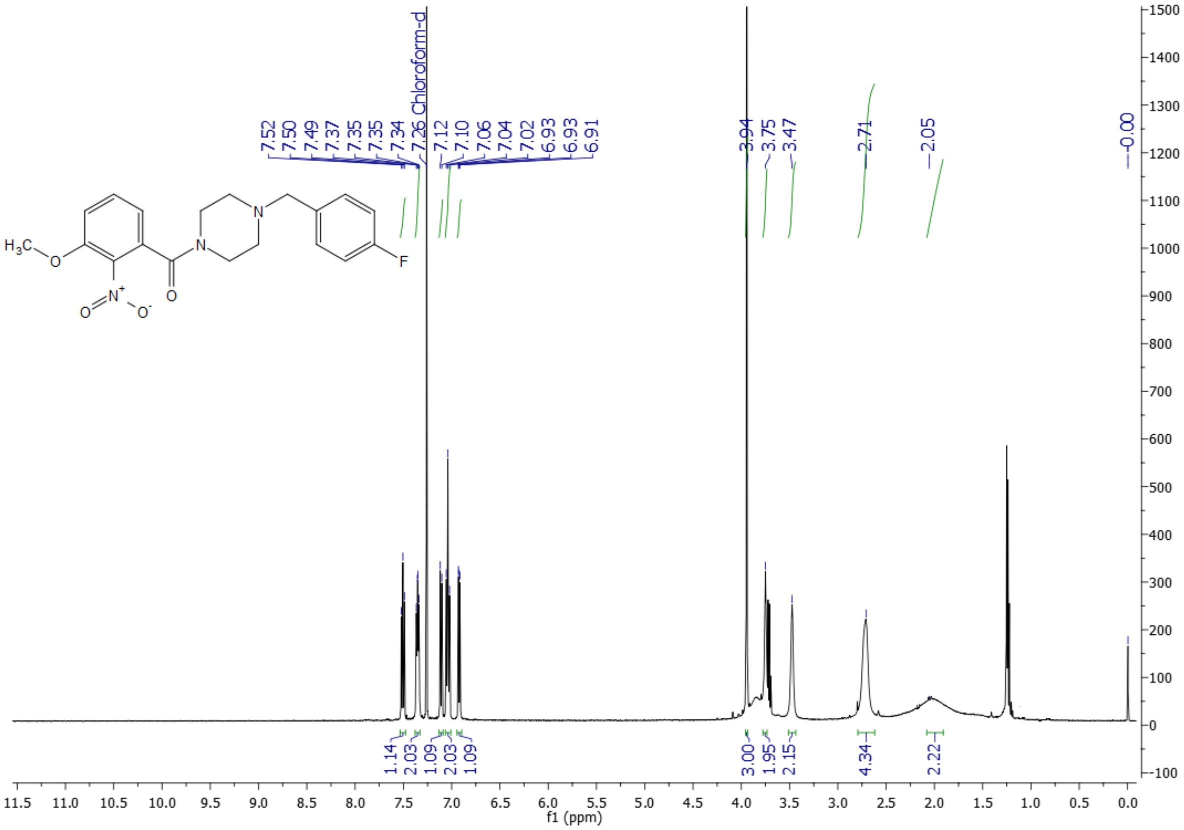

Figure S39: ^{1}H-NMR ($CDCl_3$) spectrum of 1-[4-(4-Fluorobenzyl)piperazin-1-yl]-(3-methoxy-2-nitro-phenyl)methanone (**30**)

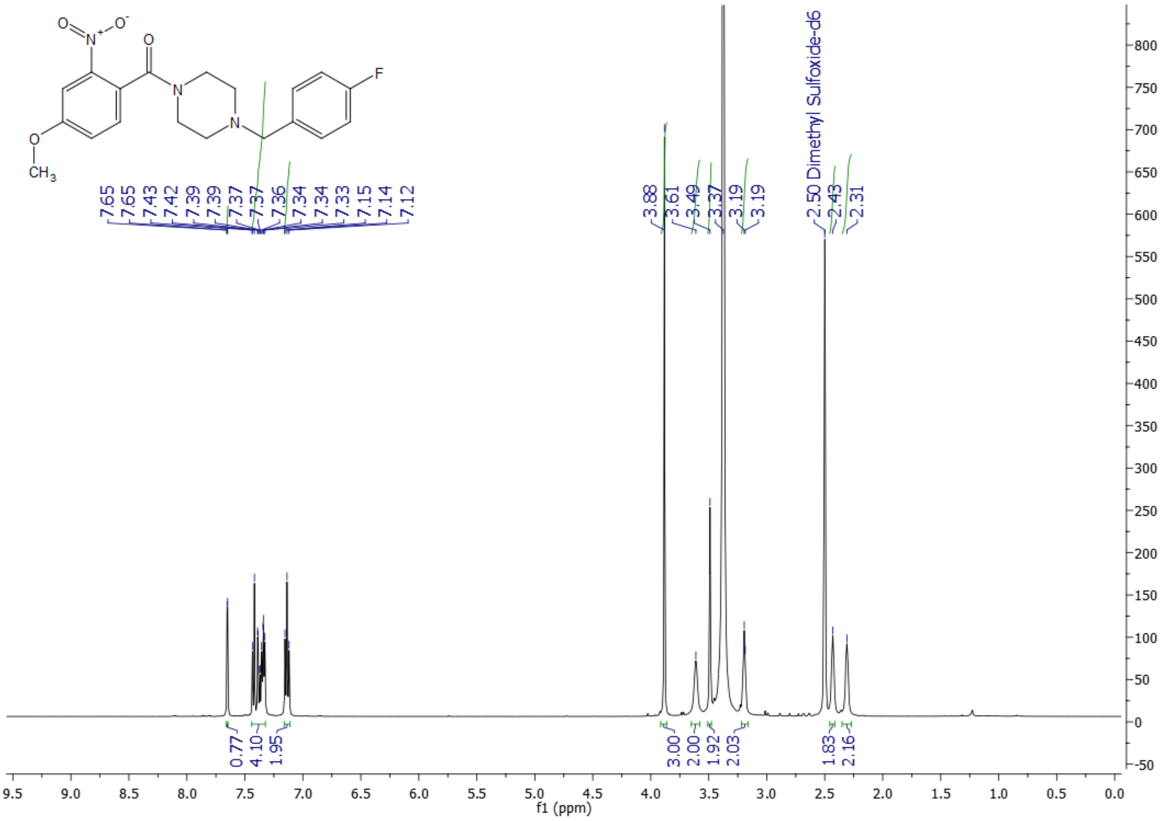

Figure S40: ^{1}H-NMR (DMSO-d6) spectrum of [4-(4-Fluorobenzyl)piperazin-1-yl]-(4-methoxy-2-nitro-phenyl)methanone (**31**)

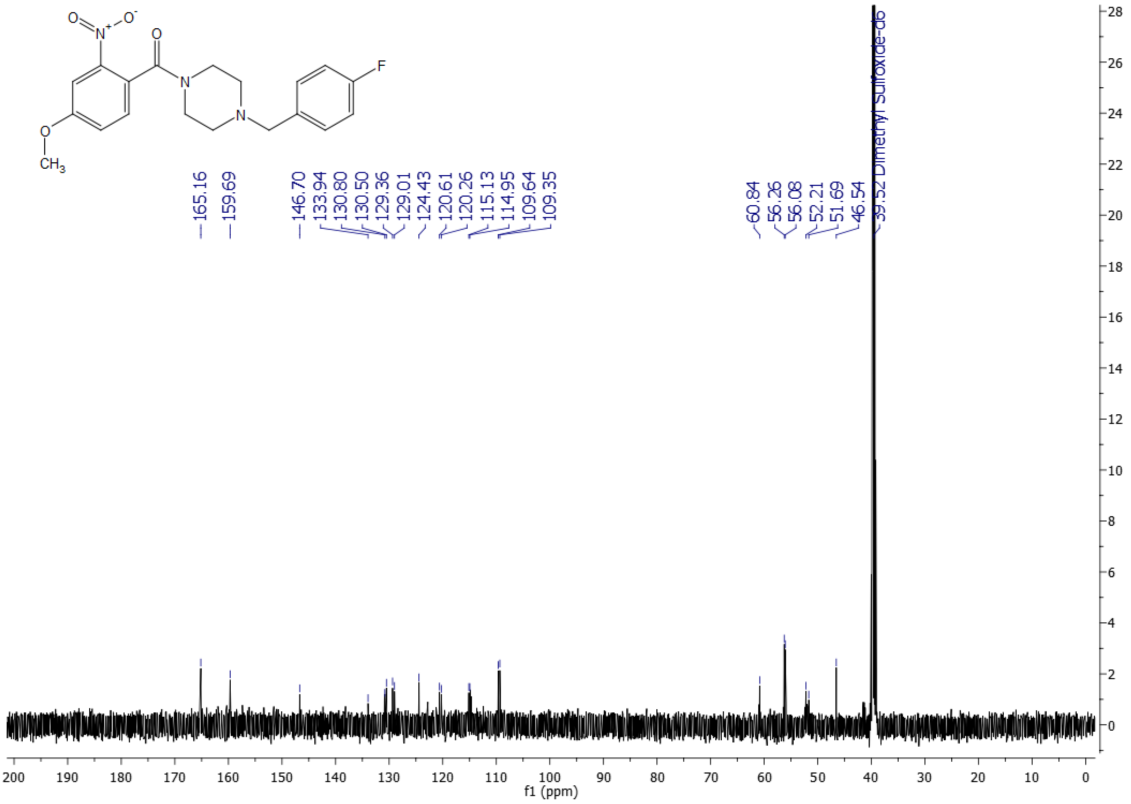

Figure S41: ^{13}C-NMR (DMSO-d6) spectrum of [4-(4-Fluorobenzyl)piperazin-1-yl]-(4-methoxy-2-nitro-phenyl)methanone (**31**)

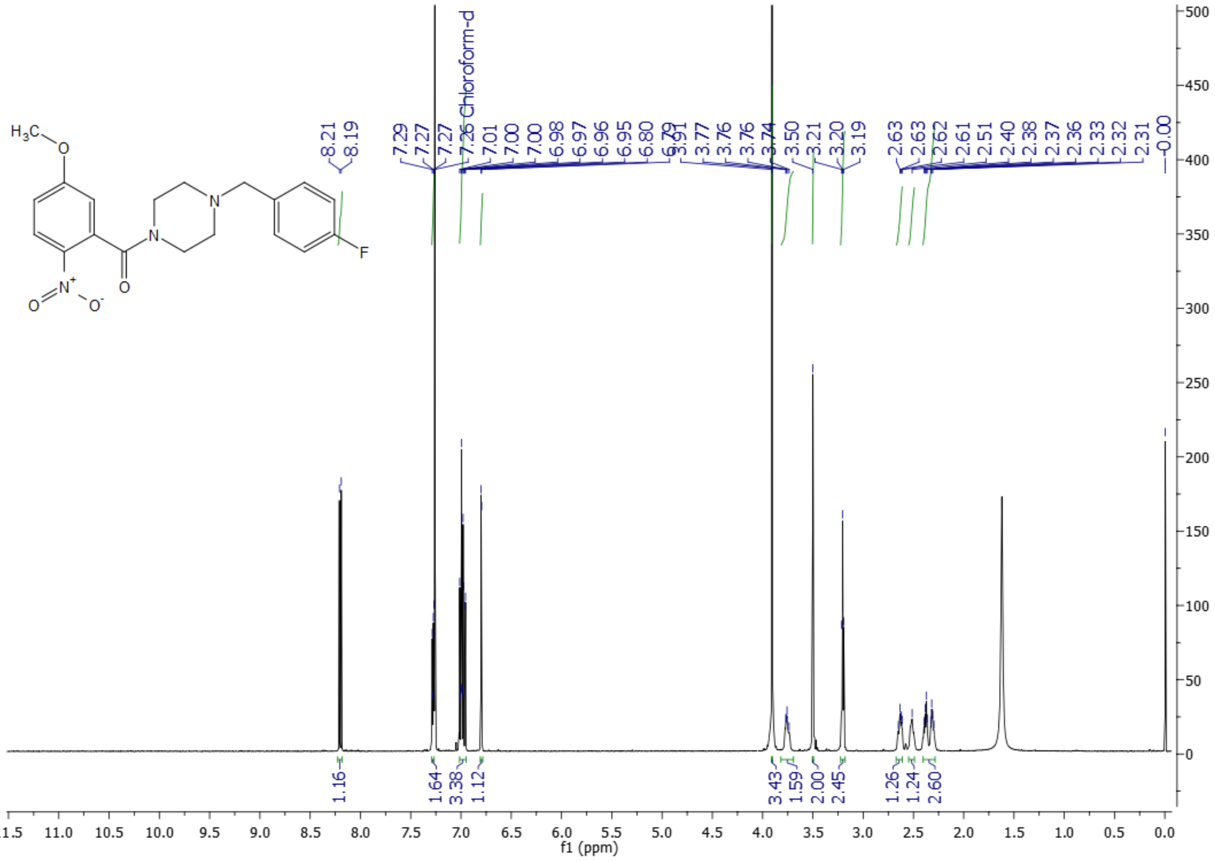

Figure S42: ^{1}H-NMR ($CDCl_3$) spectrum of 1-[4-(4-Fluorobenzyl)piperazin-1-yl]-(5-methoxy-2-nitrophenyl)methanone (**32**)

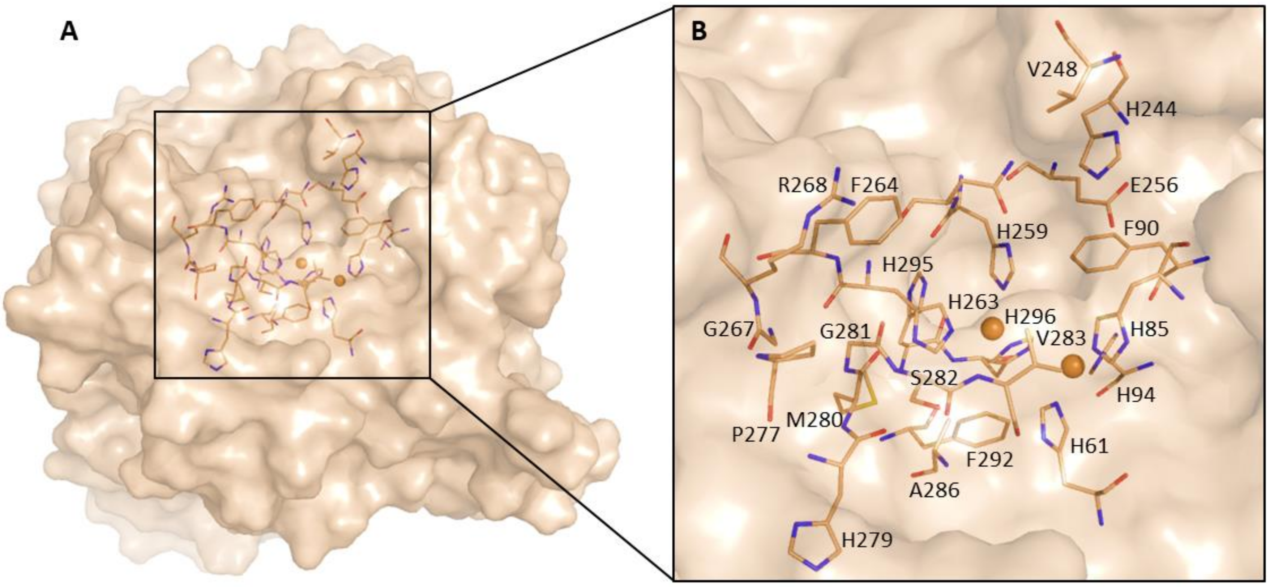

Figure S43. Representation of AbTYR binding site. A) AbTYR surface. The binding site defined for the docking calculation is lined by a black square. B) Close view of AbTYR binding site. The residues of the pocket are represented as wheat sticks.

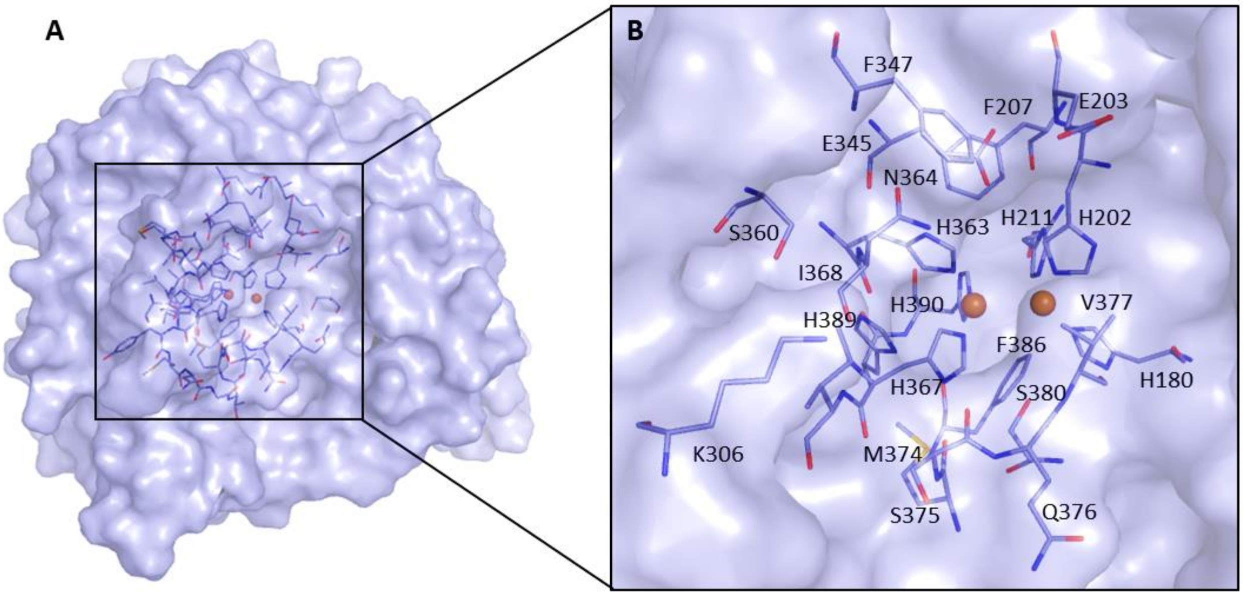

Figure S44. Representation of hTYR binding site. A) hTYR surface. The binding site defined for the docking calculation is lined by a black square. B) Close view of hTYR binding site. The residues of the pocket are represented as lightblue sticks.

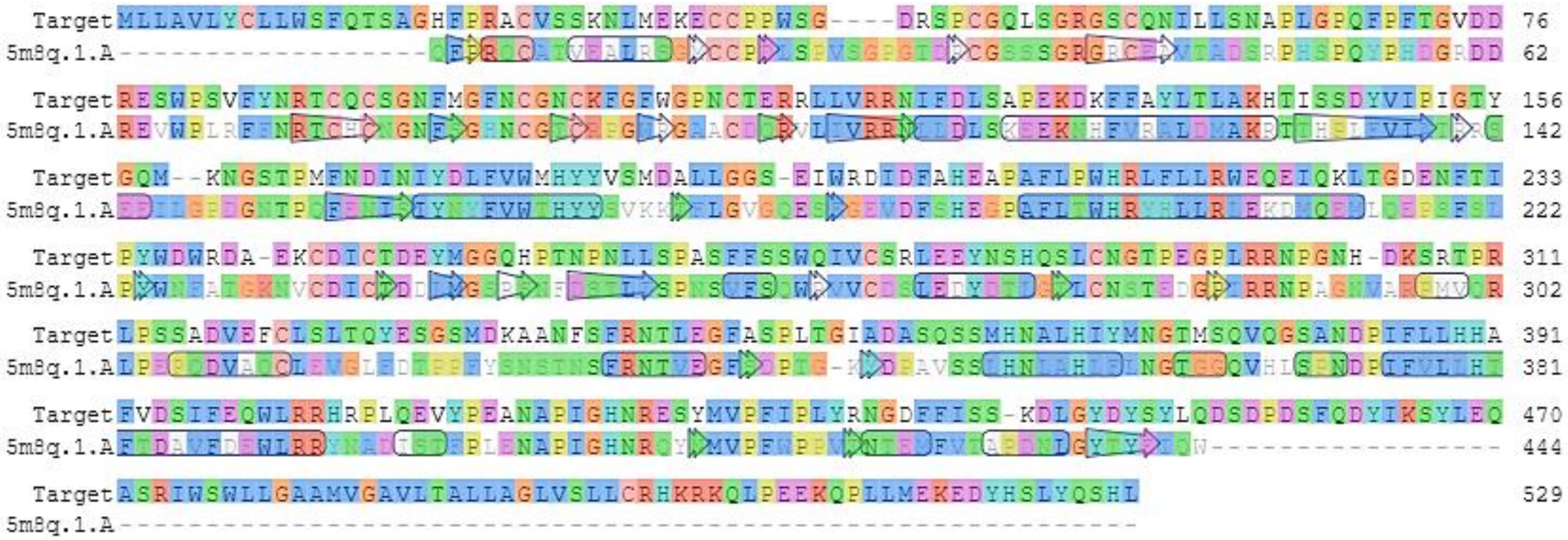

Figure S45: Sequence alignment of hTYR (target) and (TYRP-1) mutant (T391V-R374S-Y362F) 5m8q.

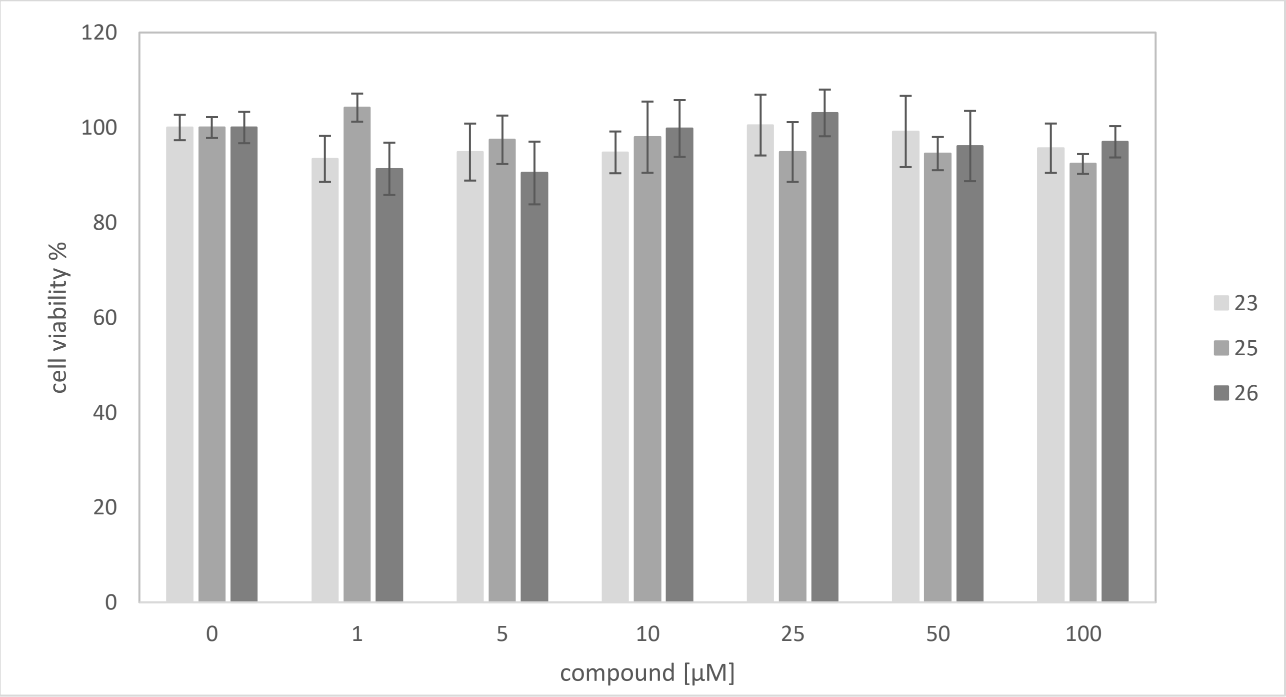

Figure S46: B16F10 melanoma cell viability after treatment with compounds **23**, **25** and **26**. The mean value and standard deviation were calculated from triplicate experiments.

Table S1: CAS numbers and smiles for compounds **7-32**

COMPOUND	CAS Number	SMILES
**7.**	**423743-29-7**	Fc1ccc(CN2CCN(C(Cc3ccccc3)=O)CC2)cc1
**8.**	**439848-20-1**	Fc1ccc(CN2CCN(CC2)C(=O)CCc3ccccc3)cc1
**9.**	**423739-67-7**	Fc1ccc(CN2CCN(CC2)C(=O)C(c3ccccc3)c4ccccc4)cc1
**10.**	**1387739-82-3**	Fc1ccc(CN2CCN(CC2)C(=O)Cc3ccccc3Cl)cc1
**11.**	**-**	Fc1ccc(CN2CCN(CC2)C(=O)Cc3cccc(Cl)c3)cc1
**12.**	**1329294-83-8**	Fc1ccc(CN2CCN(CC2)C(=O)Cc3ccc(Cl)cc3)cc1
**13.**	**1988172-38-8**	Fc1ccc(CN2CCN(CC2)C(=O)Cc3ccccc3Br)cc1
**14.**	**1985981-75-6**	Fc1ccc(CN2CCN(CC2)C(=O)Cc3cccc(Br)c3)cc1
**15.**	**1146917-07-8**	Fc1ccc(CN2CCN(CC2)C(=O)Cc3ccc(Br)cc3)cc1
**16.**	**-**	[O-][N+](=O)c1ccccc1CC(=O)N2CCN(Cc3ccc(F)cc3)CC2
**17.**	**-**	[O-][N+](=O)c1cccc(CC(=O)N2CCN(Cc3ccc(F)cc3)CC2)c1
**18.**	**-**	[O-][N+](=O)c1ccc(CC(=O)N2CCN(Cc3ccc(F)cc3)CC2)cc1
**19.**	**1796840-99-7**	COc1ccc(CC(=O)N2CCN(Cc3ccc(F)cc3)CC2)cc1
**20.**	**-**	Nc1ccccc1CC(=O)N2CCN(Cc3ccc(F)cc3)CC2
**21.**	**-**	Nc1cccc(CC(=O)N2CCN(Cc3ccc(F)cc3)CC2)c1
**22.**	**-**	Nc1ccc(CC(=O)N2CCN(Cc3ccc(F)cc3)CC2)cc1
**23.**	**-**	Fc1ccc(CN2CCN(CC2)C(=O)c3cccc(F)c3C(F)(F)F)cc1
**24.**	**-**	Fc1ccc(CN2CCN(CC2)C(=O)c3ccc(F)cc3C(F)(F)F)cc1
**25.**	**-**	Fc1ccc(CN2CCN(CC2)C(=O)c3cc(F)ccc3C(F)(F)F)cc1
**26.**	**-**	[O-][N+](=O)c1c(Cl)cccc1C(=O)N2CCN(Cc3ccc(F)cc3)CC2
**27.**	**2344352-87-8**	[O-][N+](=O)c1cc(Cl)ccc1C(=O)N2CCN(Cc3ccc(F)cc3)CC2
**28.**	**-**	[O-][N+](=O)c1ccc(Cl)cc1C(=O)N2CCN(Cc3ccc(F)cc3)CC2
**29.**	**-**	[O-][N+](=O)c1cccc(Cl)c1C(=O)N2CCN(Cc3ccc(F)cc3)CC2
**30.**	**-**	COc1cccc(C(=O)N2CCN(Cc3ccc(F)cc3)CC2)c1[N+](=O)[O-]
**31.**	**-**	COc1ccc(C(=O)N2CCN(Cc3ccc(F)cc3)CC2)c(c1)[N+](=O)[O-]
**32.**	**2345032-69-9**	COc1ccc(c(c1)C(=O)N2CCN(Cc3ccc(F)cc3)CC2)[N+](=O)[O-]

Table S2. Selected physicochemical parameters, lipophilicity, solubility and drug-likeness for compound **7-32** predicted by SwissADME(http://swissadme.ch/), and Molinspiration tools(https://molinspiration.com).

entry	MW (g/mol)*	TPSA*	TPSA**	iLogP*	miLogP**	Water solubility*	Lipinski*	Ghose*	PAINS*
**7**	312.38	23.55	23.55	3.21	2.92	Soluble	Yes<br>0 violation	Yes	0 alert
**8**	326.41	23.55	23.55	3.54	3.44	Soluble	Yes<br>0 violation	Yes	0 alert
**9**	388.48	23.55	23.55	3.79	4.31	Moderately soluble	Yes<br>0 violation	Yes	0 alert
**10**	346.83	23.55	23.55	3.44	3.55	Soluble	Yes<br>0 violation	Yes	0 alert
**11**	346.83	23.55	23.55	3.55	3.58	Soluble	Yes<br>0 violation	Yes	0 alert
**12**	346.83	23.55	23.55	3.52	3.60	Soluble	Yes<br>0 violation	Yes	0 alert
**13**	391.28	23.55	23.55	3.51	3.68	Soluble	Yes<br>0 violation	Yes	0 alert
**14**	391.28	23.55	23.55	3.64	3.71	Soluble	Yes<br>0 violation	Yes	0 alert
**15**	391.28	23.55	23.55	3.63	3.73	Soluble	Yes<br>0 violation	Yes	0 alert
**16**	357.38	69.37	69.37	2.83	2.83	Soluble	Yes<br>0 violation	Yes	0 alert
**17**	357.38	69.37	69.37	2.95	2.86	Soluble	Yes<br>0 violation	Yes	0 alert
**18**	357.38	69.37	69.37	2.92	2.88	Soluble	Yes<br>0 violation	Yes	0 alert
**19**	342.41	32.78	32.78	3.46	2.98	Soluble	Yes<br>0 violation	Yes	0 alert
**20**	327.40	49.57	49.57	2.92	2.36	Soluble	Yes<br>0 violation	Yes	0 alert
**21**	327.40	49.57	49.57	2.88	1.97	Soluble	Yes<br>0 violation	Yes	0 alert
**22**	327.40	49.57	49.57	2.78	2.00	Soluble	Yes<br>0 violation	Yes	1 alert:<br>anil_no_alk
**23**	384.34	23.55	23.55	3.27	3.32	Soluble	Yes<br>1 violation:<br>MLogP>4.15	Yes	0 alert
**24**	384.34	23.55	23.55	3.36	3.35	Soluble	Yes<br>1 violation:<br>MLogP>4.15	Yes	0 alert
**25**	384.34	23.55	23.55	3.34	3.35	Soluble	Yes<br>1 violation:<br>MLogP>4.15	Yes	0 alert

**26**	377.80	69.37	69.37	2.84	2.90	Moderately soluble	Yes<br>0 violation	Yes	0 alert
**27**	377.80	69.37	69.37	2.89	2.92	Moderately soluble	Yes<br>0 violation	Yes	0 alert
**28**	377.80	69.37	69.37	2.89	2.92	Moderately soluble	Yes<br>0 violation	Yes	0 alert
**29**	377.80	69.37	69.37	2.76	2.90	Moderately soluble	Yes<br>0 violation	Yes	0 alert
**30**	373.38	78.60	78.61	2.66	2.28	Soluble	Yes<br>0 violation	Yes	0 alert
**31**	373.38	78.60	78.61	2.65	2.30	Soluble	Yes<br>0 violation	Yes	0 alert
**32**	373.38	78.60	78.61	2.72	2.30	Soluble	Yes<br>0 violation	Yes	0 alert

***<u>SwissADME</u>** *Water solubility:* Soluble= *Log S (Ali) values between -4 and -2;* Moderately soluble= *Log S (Ali) values between -6 and -4.*

****<u>Molinspiration</u>**
